# The Dynamics of ${\mathrm{NO}}_{3}^{-}$ and ${\mathrm{NH}}_{4}^{+}$ Uptake in Duckweed Are Coordinated with the Expression of Major Nitrogen Assimilation Genes

**DOI:** 10.3390/plants11010011

**Published:** 2021-12-21

**Authors:** Yuzhen Zhou, Olena Kishchenko, Anton Stepanenko, Guimin Chen, Wei Wang, Jie Zhou, Chaozhi Pan, Nikolai Borisjuk

**Affiliations:** Jiangsu Key Laboratory for Eco-Agricultural Biotechnology around Hongze Lake, Jiangsu Collaborative Innovation Centre of Regional Modern Agriculture and Environmental Protection, Huaiyin Normal University, West Changjiang Road 111, Huai’an 223000, China; zyz@hytc.edu.cn (Y.Z.); o_kishchenko@hotmail.com (O.K.); stepanenko@hytc.edu.cn (A.S.); cgm@hytc.edu.cn (G.C.); ww376145775@163.com (W.W.); zhoujie554478400@163.com (J.Z.); pcz17878118183@163.com (C.P.)

**Keywords:** duckweed, *Spirodela polyrhiza*, nitrogen assimilation, nitrate reductase, nitrite reductase, glutamine synthetase, GOGAT, gene expression

## Abstract

Duckweed plants play important roles in aquatic ecosystems worldwide. They rapidly accumulate biomass and have potential uses in bioremediation of water polluted by fertilizer runoff or other chemicals. Here we studied the assimilation of two major sources of inorganic nitrogen, nitrate (${\mathrm{NO}}_{3}^{-}\;$) and ammonium (${\mathrm{NH}}_{4}^{+}$), in six duckweed species: *Spirodela polyrhiza*, *Landoltia punctata*, *Lemna aequinoctialis*, *Lemna turionifera*, *Lemna minor*, and *Wolffia globosa*. All six duckweed species preferred ${\mathrm{NH}}_{4}^{+}$ over ${\mathrm{NO}}_{3}^{-}$ and started using ${\mathrm{NO}}_{3}^{-}$ only when ${\mathrm{NH}}_{4}^{+}$ was depleted. Using the available genome sequence, we analyzed the molecular structure and expression of eight key nitrogen assimilation genes in *S. polyrhiza*. The expression of genes encoding nitrate reductase and nitrite reductase increased about 10-fold when ${\mathrm{NO}}_{3}^{-}$ was supplied and decreased when ${\mathrm{NH}}_{4}^{+}$ was supplied. ${\mathrm{NO}}_{3}^{-}$ and ${\mathrm{NH}}_{4}^{+}$ induced the glutamine synthetase (GS) genes *GS1;2* and the *GS2* by 2- to 5-fold, respectively, but repressed *GS1;1* and *GS1;3*. ${\mathrm{NH}}_{4}^{+}$ and ${\mathrm{NO}}_{3}^{-}$ upregulated the genes encoding ferredoxin- and NADH-dependent glutamate synthases (Fd-GOGAT and NADH-GOGAT). A survey of nitrogen assimilation gene promoters suggested complex regulation, with major roles for NRE-like and GAATC/GATTC *cis*-elements, TATA-based enhancers, ${\left(\mathrm{GA}/\mathrm{CT}\right)}_{n}$ repeats, and G-quadruplex structures. These results will inform efforts to improve bioremediation and nitrogen use efficiency.

## 1. Introduction

The application of nitrogen (N) fertilizers produced substantial crop yield increases, but N fertilizers also cause serious environmental problems [1]. Plants only absorb about 50% of the N fertilizer applied in agriculture [2]; the remainder is mainly lost to the environment, leading to soil acidification, air pollution (ammonia and nitrogen oxides), and water eutrophication (mainly nitrate (${\mathrm{NO}}_{3}^{-}$) and ammonium (${\mathrm{NH}}_{4}^{+}$)) [3]. Agriculture is responsible for 59% of the current environmental N discharge, with the remaining 41% contributed by domestic and industrial waste [4]. Aquaculture and livestock wastewater also contribute to the eutrophication of water reservoirs [5]. Water eutrophication is a global concern, and a major environmental problem for water resource management. This is especially true in China, which has increased food crop production remarkably during recent decades, largely due to the extensive application of N fertilizers. In 2020, China accounted for over 30% of the 160 megatons of N fertilizer applied worldwide [6]. The resulting runoff has led to some regions substantially exceeding the surface-water quality standard of 1 mg N/L. Remedying these problems requires transformative changes to boost N recycling; implementing these changes was recently estimated to cost China $18–29 billion per year [4].

Biological wastewater treatment using aquatic plants is a feasible, eco-friendly, and cost-effective approach [7,8,9]. For example, wetlands have been constructed worldwide to improve water quality for domestic reuse, irrigation, and environmental protection; the United States Department of Agriculture (USDA) alone has spent more than US $4.2 billion on wetland restoration and protection, especially through the Conservation Reserve Program and the Wetland Reserve Program [10,11].

Floating aquatic macrophytes, including duckweeds (Lemnaceae), represented by 37 worldwide distributed species [12,13,14], have great potential for uses in sustainable wastewater recovery [1,15]. Duckweeds’ applications rely on their capacity to efficiently take up the various contaminants responsible for eutrophication [16]. For example, about 98.0% of N and phosphorous (P) were absorbed in duckweed-populated wastewater reservoirs, with a simultaneous increase in dissolved oxygen [17,18]. Moreover, their exceptionally high propagation rates lead to fast accumulation of biomass rich in starch and protein and therefore, duckweed plants are considered a valuable feedstock for the production of biofuels [19], for livestock feed, and for human consumption [20].

Plant biomass accumulation is strongly associated with N utilization, and duckweed plants are extremely efficient at assimilating N. For example, duckweed nitrogen use efficiency (NUE) reached more than 68 kg biomass/kg N under N limitation due to N remobilization and recycling by the ubiquitin-proteasome system and autophagy [21]. However, despite intensive investigation of various duckweed species for remediation of wastewater and biomass production [22,23,24,25], studies of nutrient assimilation by duckweed species and the molecular mechanisms underlying duckweed’s remarkable NUE remain limited to a few recent studies [26,27]. By contrast, the major enzymes and molecular aspects of N assimilation have been uncovered in other plant species, primarily *Arabidopsis thaliana* and rice (*Oryza sativa*) [28].

N mostly enters into plant tissues in inorganic form (${\mathrm{NO}}_{3}^{-}$ and ${\mathrm{NH}}_{4}^{+}$) by absorption from soil facilitated by nitrate transporters (NRTs) and ammonium transporters (AMTs) [29,30]. Inorganic N can be incorporated into cellular organic compounds only in the form of ${\mathrm{NH}}_{4}^{+}$; therefore, ${\mathrm{NO}}_{3}^{-}$ is first reduced by cytosolic nitrate reductase (NR) to nitrite (${\mathrm{NO}}_{2}^{-}$), which is then imported into the plastid, where it is further reduced by nitrite reductase (NiR) to ${\mathrm{NH}}_{4}^{+}$. The ${\mathrm{NH}}_{4}^{+}$, whether taken directly from the environment or converted from ${\mathrm{NO}}_{3}^{-}$, is assimilated by glutamine synthetase (GS) into glutamine, which provides N for virtually all cellular N-containing components directly or via glutamate (Figure S1).

Higher plants contain several GS isoenzymes, which are located in the cytosol (GS1) and in the plastids (GS2) and are encoded by a small multigene family [31]. Cytosolic GS1 plays a major role in primary ${\mathrm{NH}}_{4}^{+}$ assimilation in roots and in re-assimilation of the ${\mathrm{NH}}_{4}^{+}$ generated during protein degradation and amino acid catabolism; chloroplast GS2 is involved in assimilation of the ${\mathrm{NH}}_{4}^{+}$ released during photorespiration or reduction of the ${\mathrm{NO}}_{2}^{-}$ generated by ${\mathrm{NO}}_{3}^{-}$ conversion. Glutamine-2-oxoglutarate aminotransferase (GOGAT) acts in tandem with GS2 to synthesize glutamate via the GS-GOGAT cycle. Plants have two different types of GOGAT enzymes: Fd-GOGAT (EC 1.4.7.1), which uses ferredoxin (Fd) as an electron donor, and NADH-GOGAT (EC 1.4.1.14), which uses NADH.

One important aspect of N assimilation is the plant’s preference for ${\mathrm{NH}}_{4}^{+}$ over ${\mathrm{NO}}_{3}^{-}$ as the source of N [32], a question that has attracted substantial attention because of its practical application in terms of the form of N supplied in fertilizer [2,29]. Most plants prefer ${\mathrm{NO}}_{3}^{-}$ to ${\mathrm{NH}}_{4}^{+}$, although ${\mathrm{NO}}_{3}^{-}$ uptake requires more energy than ${\mathrm{NH}}_{4}^{+}$, as absorption of ${\mathrm{NO}}_{3}^{-}$ works against a steep electrochemical gradient and ${\mathrm{NO}}_{3}^{-}$ must be reduced to ${\mathrm{NH}}_{4}^{+}$ in the plant [33]. Moreover, ${\mathrm{NH}}_{4}^{+}$ often triggers toxicity, manifested in leaves as chlorosis and a reduction of growth, but the threshold at which the symptoms become visible differs widely by species [34,35]. However, some species, such as rice [33], demonstrate a preference for ${\mathrm{NH}}_{4}^{+}$. A similar bias for ${\mathrm{NH}}_{4}^{+}$ over ${\mathrm{NO}}_{3}^{-}$ was shown for at least one duckweed species, dotted duckweed (*Landoltia punctata*) [36], which is also very tolerant to ${\mathrm{NH}}_{4}^{+}$ stress [27].

Here, we explored the utilization of ${\mathrm{NO}}_{3}^{-}$ and ${\mathrm{NH}}_{4}^{+}$ in six duckweed species representing four genera: *Spirodela* (*S. polyrhiza*), *Landoltia* (*L. punctata*), *Lemna* (*L. aequinoctialis*, *L. turionifera*, *L. minor*), and *Wolffia* (*W. globosa*). Taking advantage of the available genome sequence of great duckweed (*S. polyrhiza*) [37], we characterized the structure and expression profiles of the genes coding for eight key enzymes in N assimilation in *S. polyrhiza* grown in media supplied with ${\mathrm{NO}}_{3}^{-}$, ${\mathrm{NH}}_{4}^{+}$, or a combination.

## 2. Results

### 2.1. Identity of the Analyzed Species

**Figure 1:**
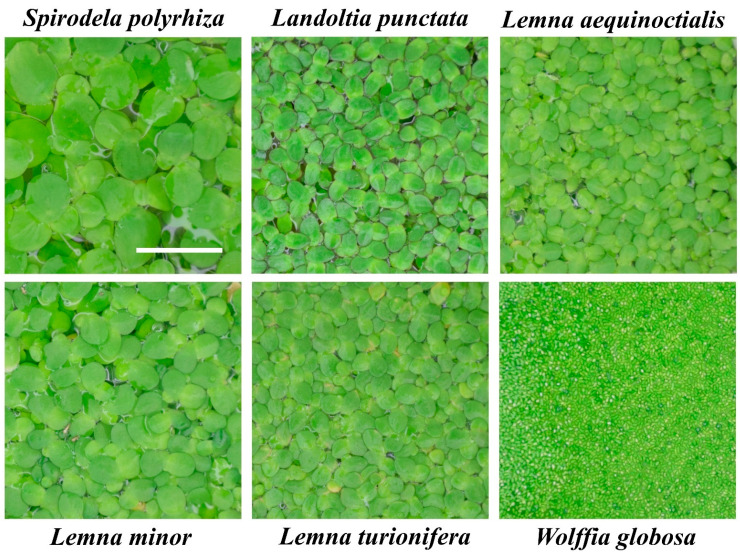
Images of the duckweed species used in the study at the exponential growth stage. All pictures were taken at the same magnification, bar corresponds to 1 cm. GenBank accession numbers for the *atpF*–*atpH* (ATP) and *psbK*–*psbL* (PSB) barcodes are: *S. polyrhiza* (NB5548), ATP MZ436185, PSB MZ436186; *L. punctata* (NB0031), ATP MZ436177, PSB MZ436178; *L. aequinoctialis* (NB0007), ATP MZ436181, PSB—MZ436182; *L. minor* (NB0020), ATP MZ436176; *L. turionifera* (NB0013), ATP MZ436179, PSB MZ436180; *W. globosa* (NB0015), ATP MZ436183, PSB MZ436184.

The duckweeds used in this study include five species isolated in Eastern China (*Spirodela polyrhiza*, *Landoltia punctata*, *Lemna aequinoctialis*, *L. turionifera*, and *Wolffia globosa*) and *Lemna minor*, collected in Kazakhstan. Prior to the N assimilation experiments, the identity of all species grown in vitro from a single frond was confirmed by barcoding through sequencing the *atpF*–*atpH* (ATP) and *psbK*–*psbL* (PSB) intergenic spacers [38] and using BLAST searches against the NCBI sequence collection [39]. The obtained ATP and PSB sequences were deposited in GenBank with the sequence accession numbers listed in Figure 1.

### 2.2. All Six Duckweed Species Demonstrate a Preference for $NH_{4}^{+}$ over NO_3_

To estimate duckweed growth responses to ${\mathrm{NO}}_{3}^{-}$ and ${\mathrm{NH}}_{4}^{+}$, all six duckweed species were cultivated under identical temperature and light conditions in 200 mL of liquid SH media. After a period of N starvation, the plants were supplied with 5 mM ${\mathrm{NO}}_{3}^{-}$, 5 mM ${\mathrm{NH}}_{4}^{+}$, or both (2.5 mM ${\mathrm{NO}}_{3}^{-}$ and 2.5 mM ${\mathrm{NH}}_{4}^{+}$) and cultured for 12 days.

All six duckweed species grew well, showing no signs of chlorosis, when 5 mM ${\mathrm{NO}}_{3}^{-}$ was used as the sole N source, even though the medium pH went up to 6.4–6.9 during the 12-day cultivation. However, when 5 mM ${\mathrm{NH}}_{4}^{+}$ was used as the sole N source, the duckweed plants showed noticeable growth defects at the late cultivation stages (Figure S2), and the medium pH dropped to 3.8–4.6 by the 4th day of cultivation (Figure S3). The first signs of chlorosis appeared on day 6 for *L. turionifera* and *L. minor*, day 8 for *S. polyrhiza* and *L. aequinoctialis*, and day 10 for *L. punctata* and *W. globosa.* On day 12, *L.* *turionifera* appeared to be the most damaged among the duckweed species, while *L. punctata* had the least number of fronds with chlorosis. The observed growth defects were less severe when the duckweeds were grown in medium with both ${\mathrm{NO}}_{3}^{-}$ and ${\mathrm{NH}}_{4}^{+}$ (Figure S2).

To determine if the severe growth defects observed during the advanced cultivation stages on the duckweed plants grown in 5 mM ${\mathrm{NH}}_{4}^{+}$ as the sole N source were due to the low pH, we adjusted the medium to the original pH of about 5.5 every other day in a second experiment. This pH correction, which more closely mirrored natural conditions in big, well-buffered water reservoirs, maintained duckweed growth for more than 2 weeks without any signs of chlorosis or depigmentation independent of the applied N source. This suggested that the growth defects and chlorosis were due to the low pH and not to the N supply.

**Figure 2:**
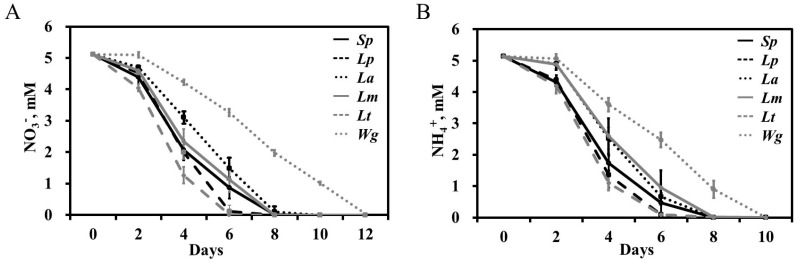
Dynamics of N uptake by six duckweed species grown in medium supplied with 5 mM ${\mathrm{NO}}_{3}^{-}$ (**A**) or ${\mathrm{NH}}_{4}^{+}$ (**B**). The y-axis shows the concentration of ${\mathrm{NO}}_{3}^{-}$ or ${\mathrm{NH}}_{4}^{+}$ remaining in the medium. *Sp*, *S. polyrhiza* (NB5548); *Lp*, *L. punctata* (NB0031); *La*, *L. aequinoctialis* (NB0007); *Lm*, *L. minor* (NB0020); *Lt*, *L. turionifera* (NB0013); *Wg*, *W. globosa* (NB0015).

We observed almost identical dynamics of N consumption by the duckweed species grown for 12 days in medium containing 5 mM of ${\mathrm{NO}}_{3}^{-}$ or ${\mathrm{NH}}_{4}^{+}$ as the sole N source (Figure 2). Five species (excluding *W. globosa*), exhausted the N in the medium by day 8 independent of the source, with *S. polyrhiza*, *L. punctata*, and *L. turionifera* showing the most rapid consumption.

**Figure 3:**
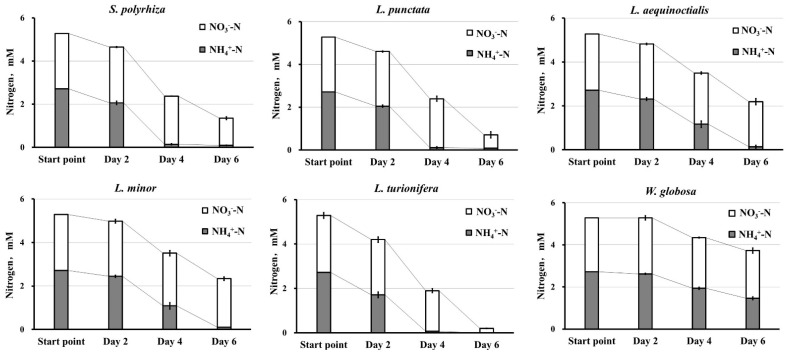
Relative dynamics of nitrate and ammonium uptake by duckweed species during six days of cultivation in medium supplied with equal amounts (2.5 mM) of ${\mathrm{NO}}_{3}^{-}$ and ${\mathrm{NH}}_{4}^{+}$.

When grown in medium supplied with both ${\mathrm{NH}}_{4}^{+}$ and ${\mathrm{NO}}_{3}^{-}$, the most common situation in the natural environment, all duckweed species demonstrated a clear preference for ${\mathrm{NH}}_{4}^{+}$, with three species (*S. polyrhiza*, *L. punctata*, and *L. turionifera*) consuming almost all of the available ${\mathrm{NH}}_{4}^{+}$ during the first four days of cultivation (Figure 3). The duckweeds started to utilize ${\mathrm{NO}}_{3}^{-}$ only when the concentration of ${\mathrm{NH}}_{4}^{+}$ dropped below 0.5 mg/L (0.04 mM).

### 2.3. Key Genes for N Assimilation in the Genome of S. polyrhiza

We evaluated the key N assimilation genes encoding NR, NiR, GS, NADH-GOGAT, and Fd-GOGAT, which have been identified as the major players in the assimilation of inorganic N in many plant species (Figure S1), using *S. polyrhiza* as the representative species due to the availability of a well-characterized whole-genome sequence [37,40,41,42]. To validate the sequences available in the GenBank, we re-sequenced the cDNA clones prepared for the four *GS* genes, *NR*, *NiR*, *NADH-GOGAT* and *SpFd-GOGAT* for the *S. polyrhiza* ecotype NB5548 used in this study (the corresponding sequence accession IDs are: *SpGS1;1*-MZ605906, *SpGS1;2*-MZ605907, *SpGS1;3*-MZ605908, *SpGS2*-MZ605909, *SpNR-*OL421561, *SpNiR-*OL421562, *SpNADH-GOGAT-*OL421563, *SpFd-GOGAT*-MZ605910).

**Figure 4:**
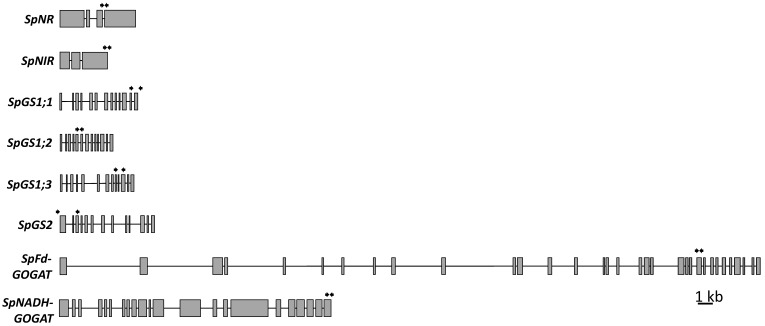
Structures of key *S. polyrhiza* genes involved in N assimilation. Exons are represented by grey boxes and lines represent introns. Arrows indicate the locations of primer binding sites used for gene expression analysis by RT-qPCR (primer sequences are listed in Table S2).

BLAST searches of the *S. polyrhiza* ecotype Sp9509 genome [37], available on the NCBI website (taxid: 29656, GCA_900492545.1), with rice protein queries revealed single genes coding for SpNR, SpNiR, SpNADH-GOGAT, and SpFd-GOGAT and four genes encoding GSs: SpGS1;1, SpGS1;2, and SpGS1;3 (which function in the cytoplasm) and SpGS2 (which is transported into chloroplasts). The exon/intron structures of the gene sequences deduced by their similarities with the corresponding rice sequences are represented in Figure 4.

#### 2.3.1. Nitrate and Nitrite Reductases

NR and NiR are encoded by single genes located on chromosome 18 of *S. polyrhiza*. For comparison, most terrestrial diploid plants have 2–3 *NR* genes and one *NiR* gene. *SpNR* is composed of four exons and three introns, typical for this plant lineage, whereas *SpNiR* has three exons and two introns (Figure 4 and Figure S4) with exons 3 and 4 that are common in other plants fused into a single exon 3 in the genome of *S. polyrhiza* (Figure S6).

Multiple alignments of SpNR and SpNiR polypeptides with NR and NiR from other species showed a high level of protein conservation among flowering plants along the whole protein sequence, with the exception of a highly diverse N-terminal region (Figures S5A and S7A). The N-terminus of NiR proteins corresponds to the transit peptide for chloroplast targeting (Figure S7A). In the phylogenetic tree, *S. polyrhiza* NiR grouped with the monophyletic clade of NiRs from monocots, whereas SpNR was more closely related to the dicotyledonous plant NR clade (Figures S5B and S7B), which probably reflects the formation of the duckweed lineage around the time when the dicots and monocots diverged [13].

#### 2.3.2. Glutamine Synthetases

Glutamine synthetases (GSs) are a family of enzymes involved in the primary incorporation of inorganic N in the form of ${\mathrm{NH}}_{4}^{+}$ (absorbed directly, produced by ${\mathrm{NO}}_{3}^{-}$ conversion, or resulting from degradation of intracellular proteins and other organic compounds) into an organic form of glutamine. There are two major GS enzyme classes encoded in plant nuclear genomes: GS1, which is localized and functions in the cytoplasm, and GS2, which is transported to chloroplasts. Most plants have a small family of three to five genes encoding cytosolic GS1 isoforms and a single gene for GS2 [43,44].

The *GS* genes, searched from the two available *S. polyrhiza* genomes using the rice protein sequence of OsGS1;2 as a query, and showing no sequence variability between *S. polyrhiza* ecotypes 9509 and 7498, were classified as *SpGS1;1*, *SpGS1;2*, *SpGS1;3*, and *SpGS2* based on sequence similarities with the corresponding *GS* genes from rice, barley (*Hordeum vulgare*), and sorghum. All analyzed duckweed *GS* genes were composed of 13 exons and 12 introns, with no size variation of exons 1 through 12 (74, 40, 104, 49, 107, 88, 129, 75, 54, 38, 160, 61 bp, respectively) for *SpGS1;1-1;3* but some variation in the intron lengths. Compared to the three *SpGS1* genes, *SpGS2* is a bit larger in size due to longer introns, and longer exons 1 and 13, which contain a chloroplast signal peptide and short variable C-terminal extension peptides (Figure 4 and Figure S8), the last one considered important for enzyme activity and do not take part in the import process to plastids [45].

Alignments of the amino acid sequences deduced from genomic DNA sequences of European and American ecotypes 9509 and 7894 and cDNA of Chinese ecotype NB5548, showed a very high degree of similarity between GSs in duckweed and other plants representing both monocot and dicot species (Figure S9A). The phylogenetic examination demonstrated that duckweed cytosolic GS1s and chloroplast GS2 form two sister groups (Figure S9B), consistent with previous studies of other plant taxa [46,47]. The separation of *GS1* and *GS2* is considered to have occurred due to a gene duplication that preceded the divergence of monocots and dicots. The degree of sequence conservation of *GS* genes can be used as a molecular clock in gene evolution studies [48]. SpGS2, SpGS1;1, and SpGS1;2 did not cluster with the respective GS sequences from monocots or dicots, while SpGS1;3 shared the highest sequence similarity with NnGS1;3 from lotus (*Nelumbo nucifera*), an aquatic dicot plant.

#### 2.3.3. Fd-GOGAT and NADH-GOGAT

GOGATs and GS2 form a GS/GOGAT cycle in plant chloroplasts, where GS catalyzes the formation of Gln from Glu and ${\mathrm{NH}}_{4}^{+}$, and Fd-GOGAT and NADH-GOGAT catalyze the transfer of an amide group from Gln to 2-oxoglutarate to produce two molecules of Glu (Figure S1). The genome of *S. polyrhiza* possesses a single gene for *Fd-GOGAT* and one for *NADH-GOGAT* (Figure 4). Similar to other characterized plant *Fd-GOGAT* genes, the duckweed homologue is composed of 33 exons and 32 introns with a total gene length of 29,677 bp. *SpNADH-GOGAT*, similar to its homologs from wheat (*Triticum aestivum*) [49] and rice [50], contains 22 exons and 21 introns with a total length of 11,391 bp (Figure 4, Figures S10 and S12). The presence of long introns is characteristic of *Fd-GOGAT* genes in many species; for example, lotus *NnFd-GOGAT* has 33 exons reaching almost 200 kb in size, while the *Fd-GOGAT* genes usually are of more than 330 kb in conifers [51].

Mature GOGAT proteins demonstrate high sequence conservation (Figures S11A and S13A). According to the phylogenetic tree shown in Figures S11B and S13B, both Fd-GOGAT and NADH-GOGAT of *S. polyrhiza* grouped with GOGAT proteins from dicots.

### 2.4. Expression of Key S. polyrhiza Genes Involved in N Assimilation

**Figure 5:**
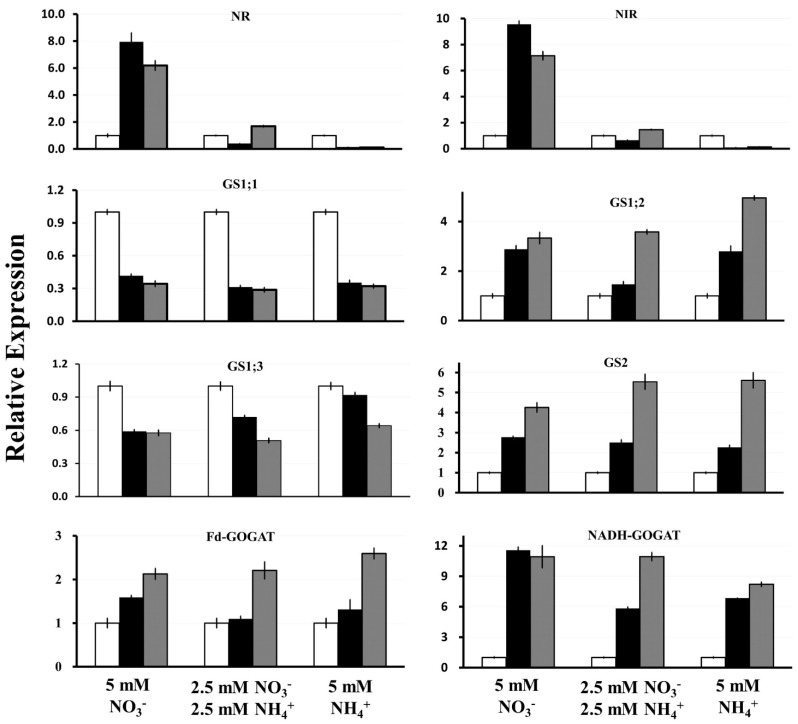
Relative expression of *S. polyrhiza NR*, *NiR*, *GS*, *Fd*-, and *NADH*-*GOGAT* genes in response to different forms of inorganic N as estimated by RT-qPCR. Gene expression levels are in relative units. Open bar: start point (day 0); black bar: day 2; gray bar: day 4. Error bars show ± SD of 3 replicates (*p* < 0.05).

We measured gene expression based on the dynamics of N uptake observed in our experiment, with the most active N consumption occurring during the first 4 days of growth after the N source was added. Therefore, *S. polyrhiza* samples for RNA isolation were taken simultaneously with medium sampling for measurement of N as represented in Figure 2 and Figure 3. All RT-qPCR reactions were performed in triplicate, normalized against the expression of two household genes (*β-actin* and *histone H3*), and related to the gene expression levels at the starting starvation point (day 0) (Figure 5).

The expression of *NR* and *NiR* was strongly induced in duckweed cultivated in medium with ${\mathrm{NO}}_{3}^{-}$ as the only N source. The highest expression was recorded for both genes at day 2, with an 8- and 9-fold increase for *NR* and *NiR*, respectively. The expression of *NR* and *NiR* then decreased slightly at day 4 following the drop in available ${\mathrm{NO}}_{3}^{-}$. When both ${\mathrm{NO}}_{3}^{-}$ and ${\mathrm{NH}}_{4}^{+}$ were supplied in the media, the relative expression of *NR* and *NiR* decreased about 2.5- and 1.5-fold on day 2, respectively, then the expression of both genes increased about 1.5-fold on day 4, indicating the start of their induction. Moreover, the relative expression of *NR* and *NiR* similarly decreased about 7- and 10-fold on day 2 and day 4, respectively, when ${\mathrm{NH}}_{4}^{+}$ was used as the sole N source.

The relative expression of *GS1;2* and *GS2* gradually increased, with a more pronounced increase in samples grown in the presence of ${\mathrm{NH}}_{4}^{+}$, reaching about 5-fold higher expression on day 4 for both genes compared to day 0. By contrast, the expression of *GS1;1* and *GS1;3* was suppressed by the addition of ${\mathrm{NO}}_{3}^{-}$ and/or ${\mathrm{NH}}_{4}^{+}$.

*Fd-GOGAT* and *NADH-GOGAT* were induced by either ${\mathrm{NO}}_{3}^{-}$ or ${\mathrm{NH}}_{4}^{+}$. The relative expression level of *Fd-GOGAT* increased 1.6- and 2.1-fold on day 2 and day 4, respectively, when 5 mM ${\mathrm{NO}}_{3}^{-}$ was used as the sole N source, and increased 1.3- and 2.6-fold on day 2 and day 4, respectively, when 5 mM ${\mathrm{NH}}_{4}^{+}$ was used as the N source. A similar expression pattern was observed when the fronds were grown in the medium containing both ${\mathrm{NO}}_{3}^{-}$ and ${\mathrm{NH}}_{4}^{+}$.

The relative expression of *NADH-GOGAT* increased 11.6- and 10.9-fold on day 2 and day 4, respectively, when 5 mM ${\mathrm{NO}}_{3}^{-}$ was used as the sole N source. Its expression increased 6.9- and 8.2-fold on day 2 and day 4, respectively, when 5 mM ${\mathrm{NH}}_{4}^{+}$ was used as the only N source, and increased 5.8- and 10.9-fold on day 2 and day 4, respectively, when both ${\mathrm{NO}}_{3}^{-}$ and ${\mathrm{NH}}_{4}^{+}$ were used.

### 2.5. Survey for Possible N-Responsive Promoter Cis-Elements in the N Assimilation Genes

**Figure 6:**
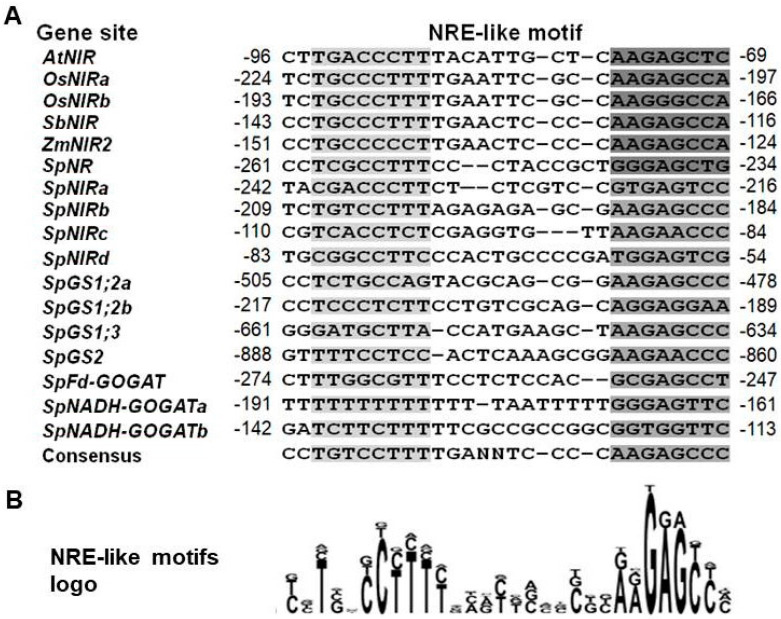
NRE-like sequences in promoters of *S. polyrhiza* N assimilation genes aligned with corresponding motifs in other plants. (**A**) Alignments of the NRE-like sequences identified in promoters of: *AtNiR* (AT2G15620, *Arabidopsis thaliana*); *OsNiR* (two motifs: *OsNiRa* and *OsNiRb*, LOC4326014, *Oryza sativa*); *SbNiR* (LOC8075200, *Sorghum bicolor*); *ZmNiR2* (LOC542264, *Zea mays*). Promoter sequences of the *SpNR*, *SpNiR*, *SpGS1;2*, *SpGS1;3*, *SpGS2*, *SpFd-GOGAT*, and *SpNADH-GOGAT* genes are from the genome of *S. polyrhiza* ecotype Sp9509, available in online databases (NCBI taxid: 29656; GCA_900492545.1). Numbers in front and at the end of the nucleotide motifs indicate their position relative to the gene translation start site. (**B**) The NRE-like motif logo displays the consensus sequences generated by CLC Main Workbench 7.6.1.

To gain further insight into the transcriptional regulation of N assimilation genes in *S. polyrhiza*, we analyzed the gene promoter regions for the presence of possible regulatory *cis*-elements. The survey revealed the presence of *cis*-elements similar to the nitrate-responsive elements (NRE), first described for the *NiR* promoter in *A. thaliana* [52] and later characterized for many other ${\mathrm{NO}}_{3}^{-}$—regulated genes [53,54,55], within the 1-kb DNA region upstream of the first ATG codon of all analyzed duckweed genes (Figure S14). While the NRE-like elements found in the promoters of *SpNR*, *SpNiR*, *SpGS1.1*, *SpGS1.2*, *SpGS1.3*, *SpGS2*, *SpNADH-GOGAT*, and *SpFd-GOGAT* showed some divergence from the canonical *A. thaliana* bipartite pseudo-palindromic sequence GACcCTT-N(10)-AAGagtcc, most of them aligned relatively well with the corresponding NREs found in *A. thaliana*, rice, sorghum, and maize (*Zea mays*) (Figure 6).

Moreover, *SpNiR* harbors four NRE-like copies positioned within the 242-bp promoter region upstream of the translation start site; *SpGS1.2* and *SpNADH-GOGAT*, which along with *SpNiR* demonstrated the highest upregulation by ${\mathrm{NO}}_{3}^{-}$ among the studied *S. polyrhiza* genes, possess three NRE-like elements each (Figure S14). Correlation between the number of NREs and the increase of nitrate-inducible expression was recently confirmed using synthetic promoters, which demonstrated that increasing the number of NREs in the promoter of rice *OsNiR*, which naturally has two NRE-like elements [56], led to a significant enhancement of N assimilation [57]. The NRE-like elements in *SpNADH-GOGAT* showed significant divergence from the canonic bipartite NRE sequence (Figure 6B), and their functionality remains to be tested.

Another relatively well-characterized molecular system for fine-tuning gene expression in response to the N supply is based on Nitrate-Inducible GARP-Type Transcriptional Repressor-1 (NIGT1) family proteins, first identified as transcriptional repressors in rice [58], and later studied in more detail in *A. thaliana* [59,60]. NIGT1 proteins demonstrate dual modes of promoter sequence recognition, binding to two types of *cis*-elements, GAATC or its reverse complement sequence GATTC, and GAATATTC [54,61]. A search for these elements in promoters of the duckweed N assimilation genes did not reveal the GAATATTC element, whereas multiple sites matching the GAATC/GATTC sequences were found in the promoters of *SpGS1.1* (4), *SpGS1.2* (6), *SpGS2* (4), and *SpNADH-GOGAT* (2), representing a potential opportunity for negative regulation by the SpNIGT1 homolog upon supply of ${\mathrm{NO}}_{3}^{-}$. None of these *cis*-elements were found in the promoters of *SpNiR* and *SpGS1.3*, while *SpNR* and *SpFd-GOGAT* both contain a single copy of GAATC/GATTC (Table S3 and Figure S14).

The presence of GAGA and/or complementary CTCT stretches, and G4-quadruplex structures, both of which are implicated in general regulation of gene transcription [62,63], is a prominent feature of the duckweed promoters analyzed in this study. All promoters of N assimilation genes, except for *SpGS1.1*, contained repetitive GAGA or CTCT stretches between −1070 and −330 nucleotides upstream of the ATG start site, with the *SpFd-GOGAT* promoter containing an exceptionally long GAGA region of 306 bp (positions −864 to −559) and a 22-nucleotide stretch of TCTC at −351 to −329 bp (Figure S14). Additionally, all analyzed promoters exhibited numerous TATA-like motifs, which represent not only important elements of a core promoter in many plant genes [64,65], but also may act as general transcriptional enhancers [66].

G-quadruplexes (G4) are secondary nucleotide structures found in guanidine-rich regions, which are implicated in various cellular processes in eukaryotic organisms [67]. The G4 structures formed along DNA or RNA strands by tetrads of guanine bases joined together via nonconventional hydrogen bonds are often located in gene promoters, introns, or 5′-untranslated regions (UTRs) and play important roles in regulation of gene functions [68,69]. The *pqsfinder* G4 prediction online tool [70] preset for the recommended scanning window of 100 bp [71] revealed characteristic patterns of G4-motif distribution in the analyzed duckweed promoters. In particular, *SpNR* and *SpNiR* promoters showed a strong G4 peak located at the same position between −265 and −290 bp relative to the translation start site, but on opposite DNA strands. This was the only G4 structure detected in the *SpNiR* promoter sequence, whereas the *SpNR* promoter had four more G4-motifs further upstream of the ATG start site (Figure S15). The promoters of *SpGS1.1*, *SpGS1.2*, and *SpGS1.3* each had one or two relatively strong G4 structures composed of more than four G4 stems situated at both DNA strands and in different locations along the 1-kb promoter sequence, while *SpGS2* possessed two relatively weak structures of three G4 units each. The *SpFd-GOGAT* and *SpNADF-GOGAT* promoters had a characteristic G4 peak directly adjacent to the gene translation start site, the 5′-UTR, which is the most common position of the G4 structures in genes of many monocot plants such as maize [72], rice [73], wheat [74], and barley [71].

## 3. Discussion

### 3.1. Duckweeds’ Preference for $NH_{4}^{+}$ as a Source of N

The duckweed species investigated in this study (Figure 1) are the ones most commonly used for wastewater remediation [16,18,22,75]. All six species demonstrated an obvious preference for ${\mathrm{NH}}_{4}^{+}$ over ${\mathrm{NO}}_{3}^{-}$ when given the choice between the two under our experimental growth conditions. A similar N source preference was previously shown for at least two representative species of duckweed, *Lemna gibba* [76] and *Landoltia punctata* [36], which are also among the most tolerant of ${\mathrm{NH}}_{4}^{+}$ stress [27]. In our experiments the consumption of ${\mathrm{NO}}_{3}^{-}$ by the duckweeds did not start until the ${\mathrm{NH}}_{4}^{+}$ was exhausted (Figure 3).

From the standpoint of cell metabolic economics, ${\mathrm{NH}}_{4}^{+}$ is the obvious choice of N as it is the only N source that can be used for building various organic compounds, and ${\mathrm{NO}}_{3}^{-}$ must be converted into ${\mathrm{NH}}_{4}^{+}$ to provide N for cellular metabolism. However, the majority of plant species prefer ${\mathrm{NO}}_{3}^{-}$ because ${\mathrm{NH}}_{4}^{+}$ causes toxicity at certain concentrations, resulting in leaf chlorosis and a reduction of growth [34,35]. Duckweed also manifests symptoms of ${\mathrm{NH}}_{4}^{+}$ stress [77], but its threshold is higher compared to other plants, which is probably one of the reasons why duckweed shows incredible adaptability and high growth rates.

### 3.2. NR and NiR Are Co-Ordinately Expressed, Stimulated by $NO_{3}^{-}$ and Suppressed by $NH_{4}^{+}$

In accordance with their functional link in the step-by-step conversion of ${\mathrm{NO}}_{3}^{-}$ into ${\mathrm{NH}}_{4}^{+}$, *SpNR* and *SpNiR* demonstrated very similar, almost identical, expression patterns in response to ${\mathrm{NH}}_{4}^{+}$ and ${\mathrm{NO}}_{3}^{-}$ (Figure 5). The expression of both genes was clearly stimulated by addition of ${\mathrm{NO}}_{3}^{-}$ as the sole N source after a period of starvation. By contrast, ${\mathrm{NH}}_{4}^{+}$ seemed to suppress *SpNR* and *SpNiR* expression, especially when it was supplied as the sole N source at a relatively high concentration of 5 mM. On the one hand, the stimulation of *NR* and *NiR* by ${\mathrm{NO}}_{3}^{-}$ has been well documented in many plant species [56,78]. On the other hand, to the best of our knowledge, we have demonstrated suppression of these genes in flowering plants at the transcriptional level by ${\mathrm{NH}}_{4}^{+}$ for the first time, while the inhibition of ${\mathrm{NO}}_{3}^{-}$ uptake by ${\mathrm{NH}}_{4}^{+}$ was previously shown for *Lemna* species [79], barley [80,81], and rice [82].

However, a similar expression switch of *NR* and *NiR* (upregulated by ${\mathrm{NO}}_{3}^{-}$ and downregulated by ${\mathrm{NH}}_{4}^{+}$) was described for a wide range of algae, and has been successfully used to establish inducible systems for transgene expression [83,84,85].

### 3.3. SpGS1;2 and SpGS2 Are Regulated in a Very Similar Manner, Which Is Different from SpGS1;1 and SpGS1;3

Our expression data showed drastic differences in the expression patterns between *SpGS1;1* and *SpGS1;3* and *SpGS1;2* and *SpGS2* (Figure 5). While the expression of *SpGS1;2* and *SpGS2* increased following addition of ${\mathrm{NO}}_{3}^{-}$ and/or ${\mathrm{NH}}_{4}^{+}$, the expression of *SpGS1;1* and *SpGS1;3* decreased. This difference might be partially explained by the functional specializations of the GS isoenzymes [47,86].

*GS1;2* transcripts are abundant in almost all plant tissues [87,88], especially as the dominant GS isoform in roots of monocot crops such as barley [89] and rice [50], where it is considered to play a pivotal role in the primary assimilation of ${\mathrm{NH}}_{4}^{+}$. In green tissues, such as leaves and stems, GS1;2 complements GS2, which is a dominant enzyme in assimilating the ${\mathrm{NH}}_{4}^{+}$ produced by photorespiration [90]. These genes are both upregulated by N, with a recent finding showing almost no difference in *GS2* expression patterns in response to ${\mathrm{NH}}_{4}^{+}$ and ${\mathrm{NO}}_{3}^{-}$ in tea tree (*Camellia sinensis*) [91]. Moreover, GS1;2 and GS2 are essential for NUE, healthy development, and accumulation of vegetative biomass, as well as stress responses [86,92]. In duckweed, in which the uptake and assimilation functions of a root and a leaf are often combined in a single assembly of a frond, *GS1;2* and *GS2* likely play a central role in the plant’s high growth rate and biomass accumulation.

In contrast to *SpGS1;2* and *SpGS2*, addition of fresh nutrient medium to an N-starved duckweed culture (start point in Figure 5) resulted in renewed vegetative growth and drastic downregulation of *SpGS1;1* and *SpGS1;3.* This is in agreement with a number of previous studies suggesting that cytosolic GS1;1 mainly functions in ${\mathrm{NH}}_{4}^{+}$ remobilization from protein breakdown during starvation and/or senescence [93,94]. The cytosolic isoforms of GS in wheat (*TaGS1.1*) [31], barley (*HvGS1;1*) [89], and oilseed rape (*Brassica napus*; *BnaGLN1.1* and *BnaGLN1.4*) [95] are all upregulated in senescing leaves. In concert with GS1.3, GS1.1 is a major contributor to N supply during seed filling [31,89,96,97]. For example, a rice mutant lacking *OsGS1;1* showed a severe reduction in grain yield [96]; wheat with all three homologs of *TaGS1.1* knocked down by clustered regularly interspaced short palindromic repeats (CRISPR)/CRISPR-associated protein 9 (Cas9) had reduced N translocation efficiency and grain filling, fewer grains per spike, and significantly reduced yield compared to the wild type [98]. To the contrary, transgenic introduction of an extra copy of native *HvGS1;1* led to higher grain yields and NUE in barley [99]. Similarly, overexpression of *ZmGln1;3* in maize resulted in an increase in kernel number, ultimately leading to a higher yield in transgenic plants compared with controls [100].

Taking these observations together, it might be concluded that, similar to other plants [101], the contribution of *SpGS1;1* and *SpGS1;3* to primary N assimilation by duckweed is much lower than that of *SpGS1;2* and *SpGS2.* Accordingly, we suggest that *SpGS1;1* and *SpGS1;3* may play a role in filling of the turion with storage nutrients. Preliminary support for this assumption can be found in RNA-seq data related to turion formation in *S. polyrhiza* [102], showing 5-fold upregulation of *SpGS1;3* and simultaneous 5-fold downregulation of *SpGS1;2.*

### 3.4. Fd-GOGAT and NADH-GOGAT Have a Complex Exon-Intron Structure, and Are Upregulated by $NO_{3}^{-}$ and $NH_{4}^{+}$

Approximately 95% of the ${\mathrm{NH}}_{4}^{+}$ produced/assimilated in plant tissues is utilized through the GS-GOGAT cycle, facilitated by coordinated actions of glutamine synthetase and glutamine synthase (GOGAT), represented by Fd- and NADH-dependent glutamine:2-oxoglutarate amidotransferases (Fd-GOGAT and NADH-GOGAT) [103,104]. Our RT-qPCR data (Figure 5) demonstrated clear stimulation of *SpNADH-GOGAT* and *SpFd-GOGAT* expression by N added after starvation, where the relative expression of *Fd-GOGAT* increased about 2-fold, and the relative expression of *NADH-GOGAT* increased by more than 10-fold. While the GOGAT isozymes are both implicated in N assimilation processes, such as primary assimilation and in photorespiration [105,106], remobilization in senescing organs [50,107], and grain development [49,108], our data hint that SpNADH-GOGAT is responsible for primarily N assimilation whereas SpFd-GOGAT is more active in N remobilization.

### 3.5. Distinctive Promoter Elements in the N Assimilation Genes in S. polyrhiza

A survey of the structural organization of promoter sequences of the analyzed genes revealed some common features, which might shed light on the role of N in gene regulation. The most intriguing finding is probably the signatures of NRE-like elements revealed in all characterized *S. polyrhiza* promoters. While some of these motifs show noticeable divergence from the canonic bipartite NRE, especially the 5′-halves of the NREs in the *SpNADH-GOGAT* promoter (Figure 6 and Figure S14), their functionality remains to be tested.

The binding of (GA/CT)_n_ repeats by a family of GAGA-binding transcription factors (GAFs) was first discovered and thoroughly characterized in *Drosophila*, where GAFs regulate numerous developmental genes in cooperation with chromatin remodeling factors [109,110]. The genomes of both *Spirodela* species, *S. polyrhiza* and *S. intermedia*, are unusually enriched in (GA/CT)_n_ repeats as documented by whole-genome surveys [37,111]. However, although numerous transcription factors with GAGA-binding properties have been identified in a number of plant species [62,112], the function of GAF-regulated transcription in plants remains largely unknown [63]. Also, in the analyzed plants, G4 motifs were enriched in the first gene exons and first introns [71]. No such enrichments were found in the analyzed duckweed genes except for *SpGS1;1*, where a strong G4 structure was identified in the antisense strand of the first intron. Therefore, our analysis identified significant enrichment of G4 structures primarily in the promoter regions of the duckweed genes involved in N assimilation.

Overall, the array of potential regulatory DNA elements revealed in the *S. polyrhiza* promoters (summarized in Table S3) suggests complex regulation of the N assimilation genes with major roles attributed to the concerted actions involving multiple NRE-like and GAATC/GATTC *cis*-elements, TATA-based enhancers, (GA/CT)_n_ repeats, and G-quadruplex structures, while the details of their individual roles and interaction will need to be uncovered in future research.

## 4. Materials and Methods

### 4.1. Plant Materials

The ecotypes used in this study were selected from the duckweed live in vitro collection recently established in the School of Life Sciences at Huaiyin Normal University, Huai’an, China (*S. polyrhiza* (collection ID: NB5548), *L. punctata* (NB0031), *L. turionifera* (NB0013), and *W. globosa* (NB0015)) were collected from small ponds and lakes at different locations between Huai’an city and Hongze lake in eastern China; *L. aequinoctialis* (NB0007) originated from the lake in a park next to People’s Square in Shanghai; and *L. minor* (NB0020) was sampled in Nursultan, Kazakhstan. To propagate the samples under sterile conditions and form a stock of live material for further experiments, the collected duckweed samples were surface sterilized in a solution containing 0.5% sodium hypochlorite and 0.1% benzalkonium bromide, washed with autoclaved water, and single fronds were put on solid agar medium supplemented with SH salts [113]. The identity of the species was confirmed by double barcoding using primers specific for chloroplast DNA intergenic spacers *atpF*–*atpH* (ATP) and *psbK*–*psbL* (PSB) following the protocol described by Borisjuk et al. (2015) [38].

### 4.2. Duckweed Cultivation Parameters and Determination of N Uptake

To accumulate biomass, duckweed plants grown on solid agar medium were initially transferred into sterile liquid SH medium supplemented with 5 g/L sucrose and cultivated at 23 ± 1 °C with a photon flux density of 50–60 μmol·m^−2^ s^−1^ provided by cool white fluorescent bulbs in a 16-h light/8-h dark cycle. After four weeks of growth, the accumulated fronds were weighed and 0.5-g portions were inoculated into open 500-mL paper containers containing 200 mL of basic SH medium (no sugar) supplemented with three different formulations of N. The first medium was supplemented with ${\mathrm{NO}}_{3}^{-}$ as the sole N source (5 mM ${\mathrm{KNO}}_{3}$, with 5 mM ${\mathrm{KH}}_{2}{\mathrm{PO}}_{4}$ replacing ${\mathrm{NH}}_{4}H_{2}{\mathrm{PO}}_{4}$ of the standard SH medium). The second formulation contained ${\mathrm{NH}}_{4}^{+}$ (5 mM ${\mathrm{NH}}_{4}H_{2}{\mathrm{PO}}_{4}$ and 5 mM $K_{2}{\mathrm{SO}}_{4}$ for the potassium salt), and the third formulation contained a mixture of ${\mathrm{NO}}_{3}^{-}$ and ${\mathrm{NH}}_{4}^{+}\;$(2.5 mM ${\mathrm{KNO}}_{3}$, 2.5 mM ${\mathrm{KH}}_{2}{\mathrm{PO}}_{4}$, 2.5 mM ${\mathrm{NH}}_{4}H_{2}{\mathrm{PO}}_{4}$, and 2.5 mM $K_{2}{\mathrm{SO}}_{4}$). The medium pH was originally adjusted to 5.5, and the cultivation medium was sampled to measure the acidity every two days. The cultivation medium was adjusted to the original pH of 5.5 every other day with HCl or NaOH during the 12-day experiments to measure N consumption.

The total nitrogen (TN) concentration was determined using standard alkaline potassium persulfate digestion followed by UV spectrophotometry as previously described [18]. The ${\mathrm{NO}}_{3}^{-}$ concentration in the growth media was measured spectrophotometrically as the difference in absorption between 220 and 275 nm [114]. The ${\mathrm{NH}}_{4}^{+}$ concentration in the growth media was measured calorimetrically using the Nessler method [115].

### 4.3. Characterization of Major Duckweed Genes Related to N Assimilation

To access the duckweed orthologues of genes encoding critical enzymes involved in N assimilation, i.e., nitrate reductase (NR), nitrite reductase (NiR), glutamine synthetase (GS), NADH-dependent glutamate synthase (GLT, traditionally referred to as NADH-GOGAT), and ferredoxin-dependent glutamate synthase (Fd-GLT, also known as Fd-GOGAT), we searched the available duckweed genome sequences using the reference rice protein sequences OsNR (XP_015622710), OsNiR (XP_015641702), OsGS1;2 (XP_015631679.1), OsNADH-GOGAT (XP_015649242.1), and OsFd-GOGAT (XP_015646712.1) as the initial queries in tBLASTn searches.

The genes coding for SpNR, SpNiR, SpGS1;1, SpGS1;2, SpGS1;3, SpGS2, SpFd-GOGAT and SpNADH-GOGAT were validated by sequencing the PCR-amplified gene regions using cDNA prepared from local *S. polyrhiza*, ecotype NB5548, mRNA as a template. The PCR fragments were amplified with gene-specific primers designed according to the in silico sequence information available at NCBI (taxid: 29656, GCA_900492545.1) for *S. polyrhiza*, ecotype 9509 [37], cloned into the pMD19-T (Takara, China) vector following the manufacturer’s instructions, and sent for custom sequencing to the Sangon Biotech (Shanghai, China). The specific primers used for gene amplification are listed in Table S1. The gene sequence assembly was carried out using the CLC Main Workbench 7.6.1.

The intron/exon structure of the duckweed genes was deduced based on similarities with the homologous genes of rice, *A. thaliana*, lotus (*Nelumbo nucifera*), and sorghum (*Sorghum bicolor*), available in GenBank [116], following the general rules of exon/intron prediction [117].

### 4.4. Phylogenetic Analysis

Mature protein sequences were compared with a selection of monocot and dicot sequences available in public databases [116,118,119,120]. Multiple alignments were generated in the CLC Main Workbench 7.6.1. Phylogenetic reconstructions were performed using the function “build” of ETE3 v3.1.1 [121]. The maximum-likelihood phylogenetic trees were constructed using RaxML v8.1.20 with model PROTGAMMAJTT and default parameters with branch supports of SH-like values [122].

### 4.5. Gene Expression Analysis by RT-qPCR

The transcript levels of eight target genes (*NR*, *NiR*, *GS1;1*, *GS1;2*, *GS1;3*, *GS2*, *Fd-GOGAT*, and *NADH-GOGAT*) were measured using reverse transcription quantitative PCR (RT-qPCR) with gene-specific primers designed based on the deduced exon sequences (Table S2, Figure 4). For gene expression analysis, total RNA was extracted from 100 μg of fresh *S. polyrhiza* fronds collected at 0, 48, and 96 h from the start of the experiment according to the protocol described in Box et al. (2011) [123]. The quality of isolated total RNA was estimated with a NanoDrop One C spectrophotometer (Thermo Fisher Scientific, USA) and 1% agarose gel electrophoresis. After DNAase treatment, 600 ng of total RNA was reverse transcribed using the Reverse Transcriptase cDNA synthesis kit (Takara, China), following the manufacturer’s manual.

The qPCR reactions were performed using CFX Connect Real-Time detection system (Bio-Rad, Hercules, CA, USA) using the UltraSybr Mixture (High Rox) supplied by CWBio (Taizhou, China). The cycling conditions were as follows: initial denaturation at 95 °C for 10 min followed by 40 cycles of 30 s at 94 °C, and 20 s at the annealing temperature of the respective primers. The SYBR Green I fluorescence was monitored consecutively after the annealing step. The quality of products was checked by a thermal denaturation cycle. Only results providing a single peak were considered. The coefficient amplification efficiency for each pair of primers was determined by 10-fold serial dilutions. The level of relative expression was calculated by the 2^−ΔΔCt^ method [124]. Expression data for the target genes were normalized using the average expression of two *S. polyrhiza* ecotype NB5548 housekeeping genes, *histone H3* and *β-actin* (corresponding accession numbers, MZ605911 and MZ605912), according to the geNorm protocol [125]. Three replicates were performed for all samples. All data were analyzed using the program BIO-RAD CFX Manager 3.1 (Bio-Rad, USA) and Microsoft Excel 2016 software.

## 5. Conclusions

The present study demonstrated that duckweeds efficiently assimilate ${\mathrm{NO}}_{3}^{-}$ and ${\mathrm{NH}}_{4}^{+}$, the main components of agricultural fertilizers and major contaminants of water reservoirs, with the specific regulation of key N-assimilation genes, in terms of changes in their expression levels in response to a supply of ${\mathrm{NO}}_{3}^{-}$ and ${\mathrm{NH}}_{4}^{+}$. The activation of genes by ${\mathrm{NO}}_{3}^{-}$ or ${\mathrm{NH}}_{4}^{+}$ as the sole N source, along with characterization of the promoter elements, details important features of N assimilation by plants.

The obtained data also provides valuable information for improvement of nitrogen assimilation efficiency, NUE, and phytoremediation of wastewater, potentially by up- or down-regulating certain genes [126], modulating the gene copy number [127], or using gene editing [128].

## Data Availability

GenBank accession numbers for the *atpF*–*atpH* (ATP) and *psbK*–*psbL* (PSB) barcodes are: *S. polyrhiza* (NB5548), ATP MZ436185, PSB MZ436186; *L. punctata* (NB0031), ATP MZ436177, PSB MZ436178; *L. aequinoctialis* (NB0007), ATP MZ436181, PSB—MZ436182; *L. minor* (NB0020), ATP MZ436176; *L. turionifera* (NB0013), ATP MZ436179, PSB MZ436180; *W. globosa* (NB0015), ATP MZ436183, PSB MZ436184. GenBank accession numbers for *S. polyrhiza* (NB5548) genes are: *SpGS1;1*-MZ605906, *SpGS1;2*-MZ605907, *SpGS1;3*-MZ605908, *SpGS2*-MZ605909, *SpNR-*OL421561, *SpNiR-* OL421562, *SpNADH-GOGAT-*OL421563, *SpFd-GOGAT*-MZ605910, *histone H3-*MZ605911, and *β-actin-*MZ605912.

## Supplementary Materials

The following supporting information can be downloaded at: https://www.mdpi.com/article/10.3390/plants11010011/s1, Figure S1: Simplified diagram of Nitrogen assimilation in plants, Figure S2: Photographic documentation of six duckweed species cultivated during 12 days on media supplied with 5 mM ${\mathrm{NO}}_{3}^{-}$, 5 mM ${\mathrm{NH}}_{4}^{+}$ as sole N source or 2.5 mM ${\mathrm{NO}}_{3}^{-}$ and 2.5 mM ${\mathrm{NH}}_{4}^{+}$, Figure S3: Comparative dynamics of pH changes during the course of duckweed cultivation over 12 days period in the medium supplied with different sources of nitrogen, Figure S4: Comparison of the exon-intron structures between *NR* gene of *S. polyrhiza* and the homologues of some representative plant species, Figure S5: Sequence alignment of NR protein from *S. polyrhiza* with other representative species and the resulting proteins phylogenetic tree, Figure S6: Comparison of the exon-intron structures between *NIR* genes of *S. polyrhiza* and other representative plant species, Figure S7: Sequence alignment of NIR protein from *S. polyrhiza* with other representative species and the resulted phylogenetic tree, Figure S8: Comparison of the exon-intron structures between *GS* genes of *S. polyrhiza* and other representative plant species, Figure S9: Sequence alignment of GS proteins from *S.polyrhiza* with other representative species and the resulted phylogenetic tree, Figure S10: Diagrammatic representation of the structure of *Fd-GOGAT* genes, Figure S11: Sequence alignment of Fd-GOGAT protein from *S.polyrhiza* with other representative species and the resulted phylogenetic tree, Figure S12: Diagrammatic representation of the structure of *NADH-GOGAT* genes, Figure S13: Sequence alignment NADH-GOGAT protein from *S.polyrhiza* with other representative species and the resulted phylogenetic tree, Figure S14: Nucleotide sequences representing 1 kb promoter regions upstream of the starting ATG codon of six nitrogen assimilation genes of *S. polyrhiza* with marked locations of NRE-like *cis*-elements, GAGA and TCTC stretches and TATA-boxes, Figure S15: Patterns of G-quadruplex structures predicted for promoters of key *S. polyrhiza* genes involved in nitrogen assimilation; Table S1: List of RT-qPCR primers used for evaluating expression of the nitrogen assimilation genes in *S. polyrhiza* ecotype NB5548, Table S2: Distribution of potential regulatory DNA *cis*-elements along 1 kb promoter region upstream of the translation start of eight *S. polyrhiza* genes related to N assimilation, Table S3: List of primer used for cloning selected nitrogen assimilation genes of *S.polyrhiza* ecotype NB5548.
