# Supplementary material for: The Dynamics of NO3− and NH4+ Uptake in Duckweed Are Coordinated with the Expression of Major Nitrogen Assimilation Genes
Source: Plants (Basel). 2021 Dec 21;11(1):11. doi: 10.3390/plants11010011 (PMC8747334; doi:10.3390/plants11010011)
Supplement: Supplementary file 1 [file plants-11-00011-s001.zip › plants-1479805-supplementary.pdf]

Table S1. List of RT-qPCR primers used for evaluating expression of the Nitrogen assimilation genes in *S. polyrhiza* ecotype NB5548.

Gene	Primer name	Forward	Primer name	Reverse	PCR product, length in bp
NR	nr-f2	CTTCTGGTCCGTCGATCTC	nr-r3	CCAGCAGTTGTTCATCATTC	136
NIR	nir-f4	GAGGGAGGATGACGATGAAG	nir-r4	GTGAAGCAGAAGCGACGAG	108
GS2	GS2-f1	CATCGCGGAGTACATTTGG	GS2-r1	CACTTCACTGTCTTCTCCTG	154
GS1;1	GS1-6-5	GAGCAAAATGGGAAAGGG	GS1-6-4	GCTGCTGGTGATGATATTGTG	134
GS1;2	GS1-F5	TTCTCGTCATGTGCGACTCC	GS1-R7	CTCCTGCTCCAGTCCATACC	131
GS1;3	GS1-5 qF11	GGCGTCCTCCTGTCGTTT	GS1-5 qR11	CTTCTGATCTCGTCCATGC	119
Fd-GOGAT	g8-f4	ATACATTGCAAGACTGAGG	g8-r5	TGGTGAAGGTCATAGATAAGC	114
NADH-GOGAT	ng-f3	CTTGAAGGACGATGGCAGC	ng-r4	TATGAGTTCTTCCACACTAGG C	131
Histon3	H3dwqF1	CGCAAGTACCAGAAGAGCAC	H3dwqR1	CGAAGAGACCCACGAGGTAG	160
Actin	ActindwF1	TGTTTTCCCAAGTATCGTC	ActinR5	TCCCAGTTGGTGACGATT	153

Table S2. Distribution of potential regulatory DNA *cis*-elements along 1kb promoter region upstream of the translation start of eight *S. polyrhiza* genes related to N assimilation. The numbers for NRE-like elements and $(GA/CT)_n$ repeats indicate their position related to the first gene ATG. Numbers for GAATC/GATTC, TATA-like and 4G elements show frequencies of those elements.

Promoter / *cis*-element	NRE-like	GAATC/GATTC	TATA-like	$(GA/CT)_n$	4G
NR	-41 to -72<br>-234 to -259	1	1	-710 to -796	5
NiR	-54 to -83<br>-84 to -108<br>-184 to -209<br>-216 to -240	ND	1	-796 to -808	1
GS1;1	-134 to -159	4	2	ND	2
1;2	-189 to -217<br>-477 to -503	6	1	-1010 to -1068	4
GS1;3	-634 to -659	ND	1	-368 to -400	3
GS2	-89 to -117<br>-124 to -148<br>-860 to -888	2	1	-541 to -585<br>-700 to -728	2
Fd-GOGAT	-247 to -271<br>-351 to -376	1	3	-329 to -351<br>-559 to -864	1
NADH-GOGAT	-86 to -109<br>-113 to -140<br>-161 to -191	2	5	-658 to -714	6

Table S3. List of primer used for cloning selected Nitrogen assimilation genes of *S.polyrhiza* ecotype NB5548.

Gene	Primer name	Forward	Primer name	Reverse	PCR product length in bp
NR	SpNRcl F1	TGTTCATCCATCTGTCGGG	NR-R3	CCAGCAGTTGTTCATCATTC	1454
NR	NRF2	CTTCTGGTCCGTCGATCTC	SpNRcl R1	AATGGAAGGGAGGGACTGG	1450
NIR	SpNIRcl F1	ATCACCCGCCGACCTCGTA	SpNIRcl R2	GCCACCAGCCCTCTCATCA	1445
NIR	SpNIRcl F2	GTGACGACCAGCAAGCAGA	SpNIRcl R1	CACCGTGAAGCAGAAGCGAC	1395
GS2	GS2-7-1	GTTGCAGAGCGGAGGAGAAGA G	GS2-7-2	ATGAAATAACAGACTGGGCGGA G	1392
GS1;1	GS1-6-2	CGCCGACATCAACACCTTCC	GS1-6-3	AGGATAAACTGGAGATGGGG	718
GS1;1	GS1-6-1	CTGAATCTTCCTTCCCCTCTC	GS1-6-4	GCTGCTGGTGATGATATTGTG	1134
GS1;2	GS1-2-1	CTTCTGACTTTTGGGTGCGTCC	GS1-2-2	GAAGGGGGGAGGACTTACTAGA G	733
GS1;2	GS1-2-1	CTTCTGACTTTTGGGTGCGTCC	GS1-2-4	GGAATGAGAAGGAACCCAGGA GG	1360
GS1;3	GS1-5-4	CATGGCTCTTCTCAACGAC	GS1-5-2	CTTCTTCTTCTCTCTCCTAGGG	1088
Fd-GOGAT	g8-23f	GGCTATCTGTCAATCTAGGG	g8-23r	ACTCCGCTCTTAAATCCACC	526
Fd-GOGAT	gogat8-f2	TCTCCTCCTCCTCCTCTTCC	gogat8-r1	ACTAGATGGTGAGAAGGAGG	5224
NADH-GOGAT	SpNADH-GOGAT F1	ACATACGCCTTCCAGTTT	SpNADH-GOGAT R1	ATGATATTAGAGCTGGTCC	1428
NADH-GOGAT	SpNADH-GOGAT F2	CCTGAAAGAAAGGCTCTATAC	SpNADH-GOGAT R2	CCCTAGCAAGAATCTCAAAA	1417
NADH-GOGAT	SpNADH-GOGAT F3	TTGGTCTTTCTTCTGAGGT	SpNADH-GOGAT R3	TATTGAGCTGCTTCTGGT	1459
NADH-GOGAT	SpNADH-GOGAT F4	GAACAAGTAGTCAACAGCA	SpNADH-GOGAT R4	GGAAGTTATTCGTCTCAAG	1357
NADH-GOGAT	SpNADH-GOGAT F5	CAAGATCCCAGAATTCAAC	SpNADH-GOGAT R5	GTCGCCATTCTCTTTATGA	1399

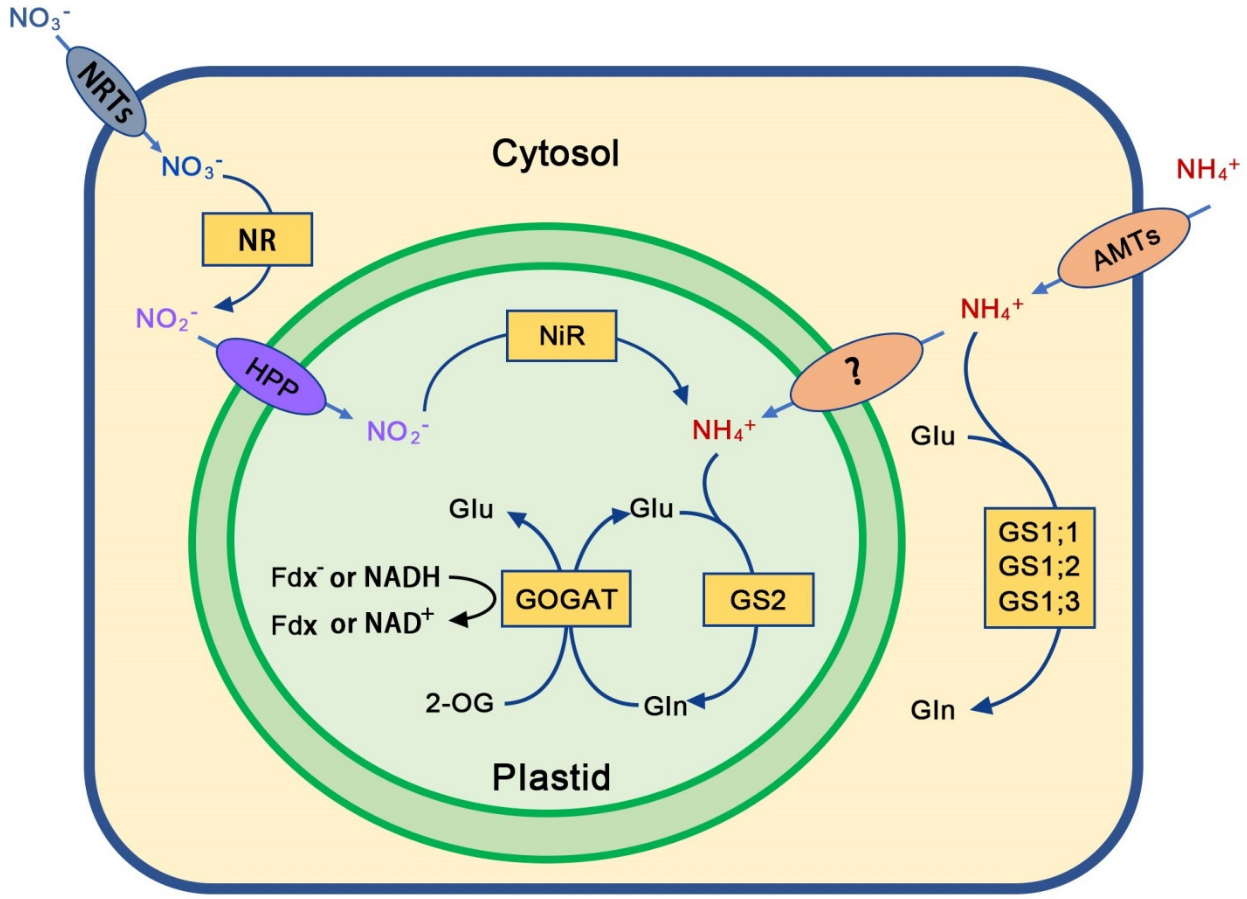

Figure S1. Simplified diagram of Nitrogen assimilation in plants. **NRTs,** nitrate transporters; **AMTs**, ammonium transporters; **HPP,** histidin-prolin-prolin containing protein; **NiR**, nitrite reductase; **NR**, nitrate reductase; **GS1;1, GS1;2, GS1;3,** tree isoforms of major cytosolic glutamine synthetases; **GS2,** chloroplast glutamine synthetase; **Fd-GOGAT**, ferredoxin-dependent glutamate-oxoglutarate-aminotransferase; **NADH-GOGAT**, NADH-dependent glutamate-oxoglutarate-aminotransferase; **?** – unknown transporter; **2-OG**, 2-oxoglutarate; **Glu**, glutamate; **Gln**, glutamine;

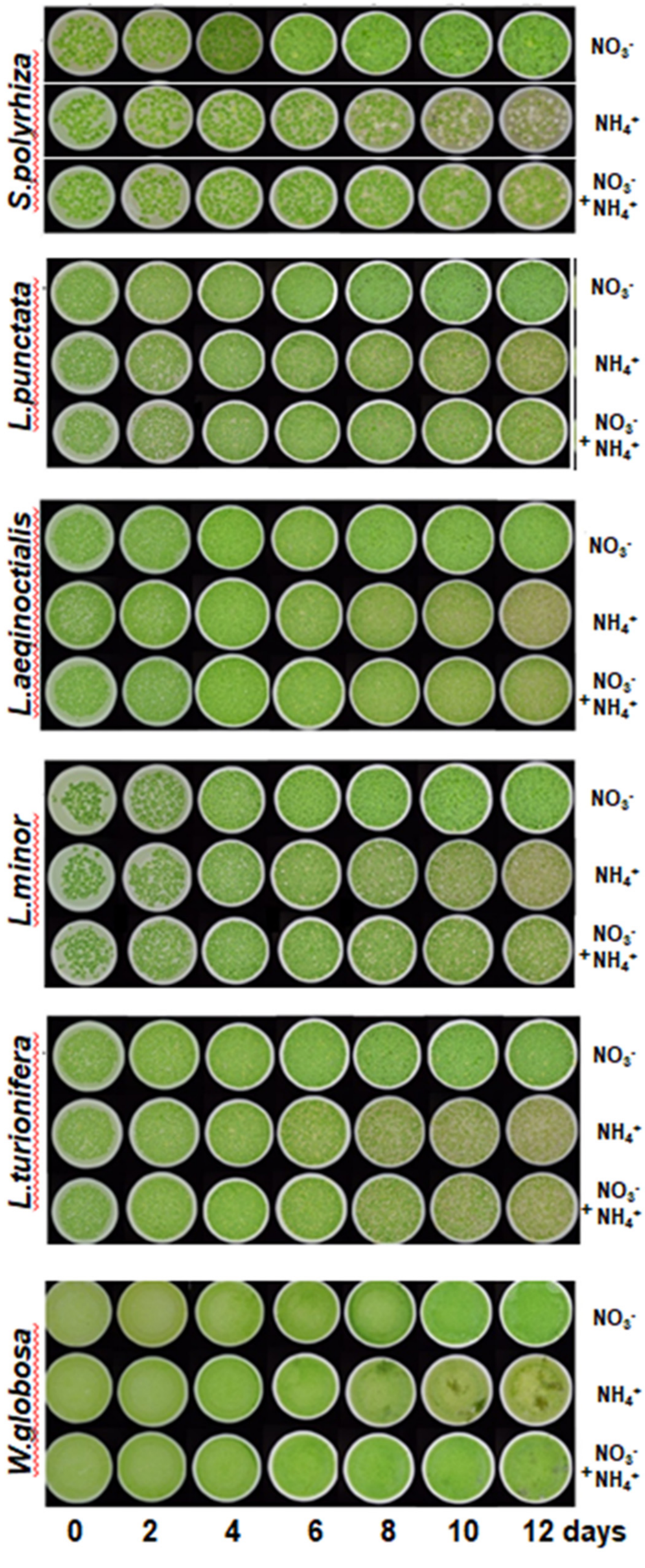

Figure S2. Photographic documentation of six duckweed species cultivated during 12 days on media supplied with 5 mM NO_3^-, 5 mM NH_4^+ as sole N source or 2.5 mM NO_3^- and 2.5 mM NH_4^+.
0 – start point, 2, 4, 6, 8, 10, 12 days after inoculation.

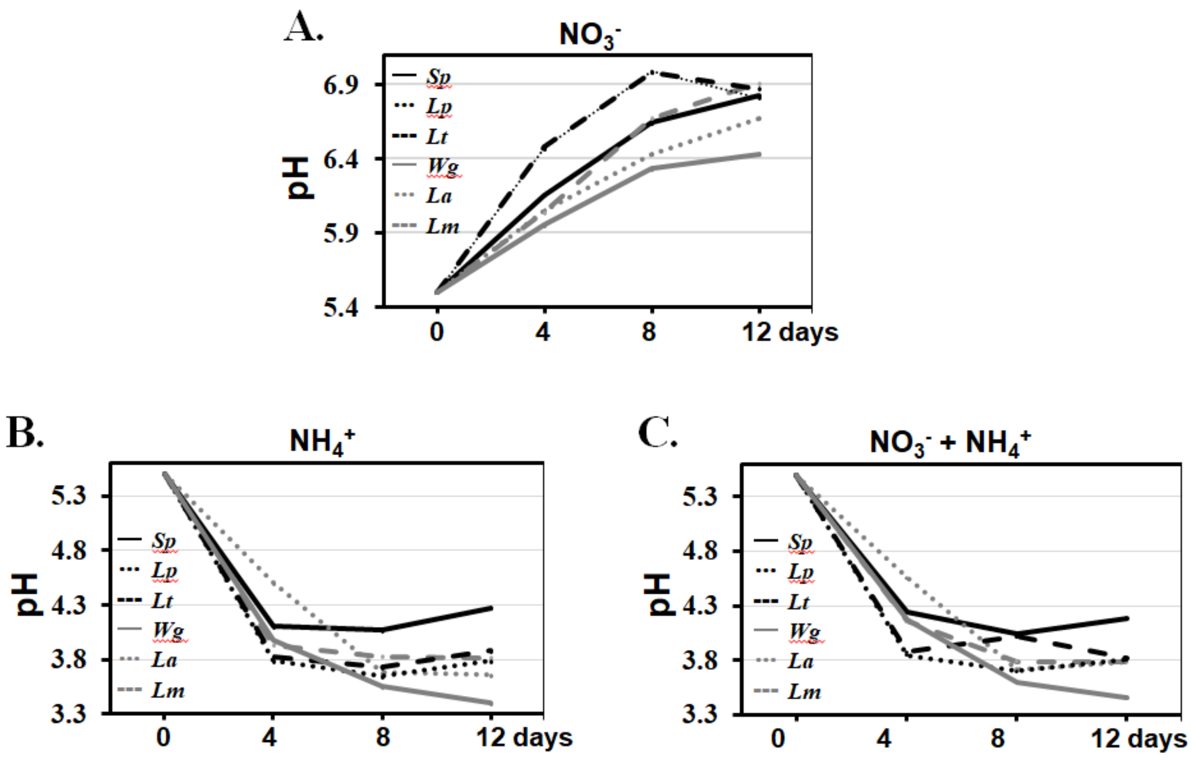

Figure S3. Comparative dynamics of pH changes during the course of duckweed cultivation over 12 days period in the medium supplied with different sources of Nitrogen. (**A)** medium supplied with 5 mM NO_3^-, (**B)** medium supplied with 5 mM NH_4^+; (**C)** medium supplied with 2.5 mM NO_3^- and 2.5 mM NH_4^+ (mix).

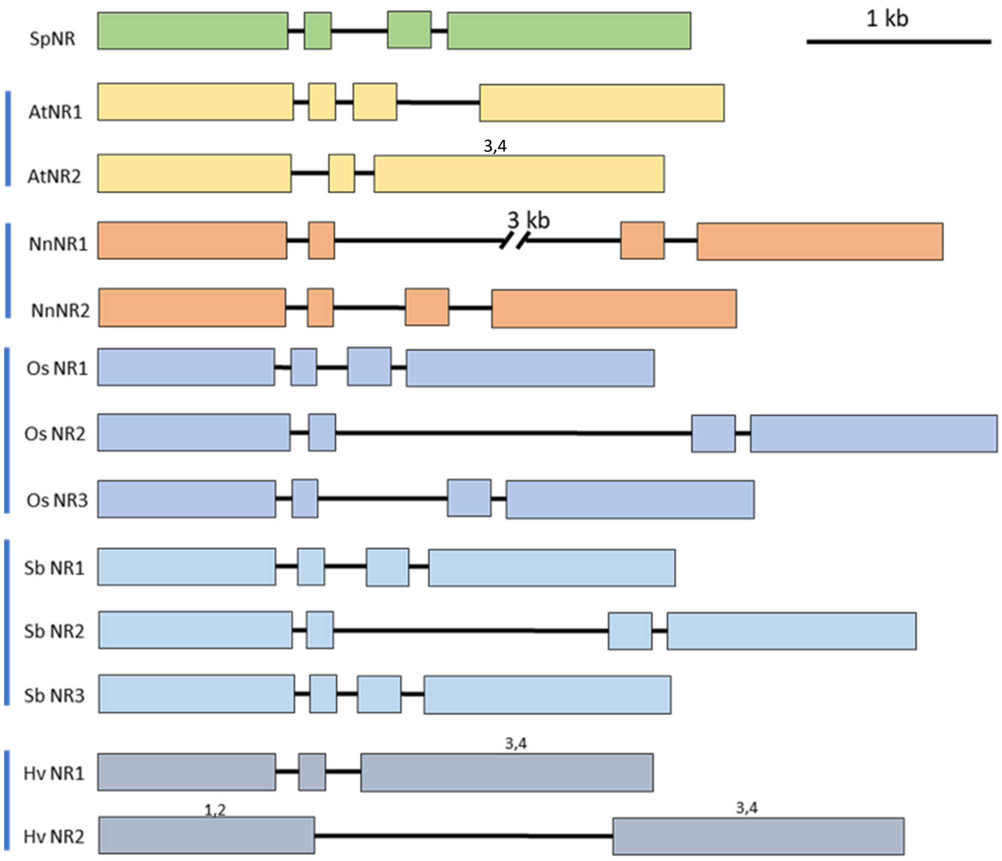

Figure S4. Comparison of the exon-intron structures between *NR* gene of *S. polyrhiza* and the homologues of some representative plant species: *AtNR1* (*A. thaliana,* At1g77760); *AtNR2* (*A. thaliana* At1g37130); *NnNR1* (*N. nucifera,* LOC104589753); *NnNR2* (*N. nucifera,* LOC104589330); *OsNR1* (*O. sativa,* LOC4330867); *OsNR2* (*O. sativa,* LOC4345795); *OsNR3* (*O. sativa,* LOC4345798); *SbNR1* (*S. bicolor,* LOC8078943); *SbNR2* (*S. bicolor,* LOC8083522); *SbNR3* (*S. bicolor,* LOC8055422), *SpNR* (*S. polyrhiza* CP019094, the 18th chromosome, position from 2346072 to 2349117). Coding sequences are colored boxes. Introns are black lines. The bar corresponds to 1kb of DNA sequence.

A

```
                    20                    40                    60                    80
SpNR      MVTSVDNRRF GHLEKGGGKV A--------- ---------- ------SPFV SGAGDSFPNG GAASFRRRHH ------HHP- 48
OsNR3     MAASVQPRQ- FGHLE-PGSA PVCGAASSNG AKAYPP-ANG IPRRADSPVR GCGFP----- --PLVSPPSR --K-PPSDGS 67
OsNR2     MAASVQPRQ- FGHLE-PGSA PVRGAASSNG AKAYPP-ANG IPRRADSPVR GCGFP----- --PLVSPPPR --K-PPSDGS 67
SbNR2     MAA-VEPRQ- FGRLE-PGVA -------TNG AKAYPPPASH LPRRADSPVR GCGFP----- --PLVSPPPR --SNKPDDAS 61
HvNR2     MAASVEPRQP FGRLDAPATA PTARAPGSNG ---------- IRRRADSPVR GCGFP----- --SLISPP-R --KGRVAEEE 60
BdNR2     MAASVEPRQ- FGRLETPVRA ----GAGTNG ---------- IRRRADSPVR GCGFS----- --PLISPP-R --KSCVHEEA 55
SbNR3     MSTCVEPPTH LANLDPGAAA QRLPYPALPG --------DD VLRR--SSVS GSSFV----- --DVAAPVSS DGKKADDDAS 63
OsNR1     MAASVEYK-- LAPHPWASNA P--------- ---------- -------SSN ---L------ --DLF----P SGGGKRRSGS 37
SbNR1     MAASVDRH-- LAPHPWPGNA P--------- ---------- -------PKS ---F------ --DMFRSGGP GGGGKRRAGS 41
HvNR1     MAASVEYNRQ VSAHPWPTNA Q--------- ---------- -------P-- KAAF------ --DLFSS--- SGGGRRRSGA 41
BdNR1     MATTVDYNRQ ASTHPWPSNA Q--------- ---------- -------PNN KLTF------ --DLFSS--- NSGGNHREY- 42
AtNR2     MAASVDNRQY ARLEP----- ------GLNG VVRSYKPP-V PG-RSDSPKA HQNQTTN--- -QTVFLKPAK VH----DDDE 59
AtNR1     MATSVDNRHY ----P----- ------TMNG VAHAFKPPLV PSPRSFDRHR HQNQTLDVIL TETKIVKETE VITTVVDSYD 65
NnNR1     MEGSVENWQF ICREP----- ------CLSG PVHGFKTAF- -NNLSDSPVR SCNFS----- A-PELSRPNQ LAVM-----E 57
NnNR2     MAASVENRQF SRLEP----- ------GLSG VVHGFKAAS- -NRRSDSPAR VCNFPS---- ATPELSRPMK PAPM-----D 58
Consensus MAASVEPRQ- FGRLP-PGNA --------NG ---------- --RR-DSPVR GCGFP----- --PLFSPP-R --KG--XDGS

                   100                   120                   140                   160
SpNR      -EDSDDEDSD YEEYIAALKA SS-----GAG EIEPSAL-DA RDEATADAWV ERNSSLIRLT GKHPFNSEPP LRRLMHHGFI 121
OsNR3     --DDEEEEQ- -EDWR--ELY GSHL---QLE V-EPSV-RDA RDEGTADAWI ERNPLLIRLT GKHPLNCEAP LARLMHHGFI 136
OsNR2     --DDEEEEQ- -EDWR--ELY GSHL---QLE V-EPPV-RDA RDEGTADAWI ERNPSLIRLT GKHPLNCEPP LARLMHHGFI 136
SbNR2     PSDDDDDDD- -EDWR--SLY GSHL---QLE V-EPAV-HDA RDEGTADAWI ERNPSLVRLT GKHPFNCEPP LARLMHHGFI 132
HvNR2     EDDDDEDDEG HEDWR--EAY GSHL---QLE V-EPST-RDP RDEGTADAWI ERNPSLIRLT GKHPLNCEPP LARLMHHGFI 133
BdNR2     SSDEEEEEQ- -QDWR--ELY GSHL---QSE V-EPSV-RDP RDEGTADAWI DRNPSLVRLT GKHPLNCEPP LSRLMHHGFI 126
SbNR3     SSDDDDDEHQ DDDHHRHETY GSHYLRRRLG V-EPSVQHDP RDEGTADAWV ERSASLIRLT GKHPFNGEPP LRRLMEHGFV 142
OsNR1     ETDSDDEDSI PPDWR--SLY -----HPRLE VAEPAV-KDP RDEATSDAWV RRHPALVRLT GKHPFNSEPP LPRLMSHGFI 109
SbNR1     DSDSEDEDTI PPDWR--SLY -----LPRLE V-EPPV-HDP RDEATSDAWV RRHPALVRLT GKHPFNSEPP VPRLMAHGFI 112
HvNR1     DSDSDDEDSV PPDWR--SLY -----SPRLD V-EPSV-KDP RDEATSDAWV KRHPALVRLT GKHPFNSEPP LPRLMSHGFI 112
BdNR1     -SDSDDEDNI PPDWR--SLY -----RPRLE V-DPPV-RDP RDEATSDAWV RRHPALVRLT GKHPFNSEPP LPRLMSHGFI 112
AtNR2     DVSSEDEN-- -ETHNSNAVY YKEMIRKSNA ELEPSVL-DP RDEYTADSWI ERNPSMVRLT GKHPFNSEAP LNRLMHHGFI 135
AtNR1     DSSSDDED-- -ESHNRNVPY YKELVKKSNS DLEPSIL-DP RDESTADSWI QRNSSMLRLT GKHPFNAEAP LPRLMHHGFI 141
NnNR1     ESSSEDEG-- -DF------D WKDHLQTANR ELEPSIL-DP RDEGTPDSWI ERNPSMIRLT GKHPFNSEPQ LTQLMHHGFI 127
NnNR2     EESSEDEG-- -DS------Y LKEQLQKANS EVEPSIL-DS RDEATADSWI ERNPSMIRLT GKHPFNSESP LTRLMHCGFI 128
Consensus XSDSDDED-- -EDWR--XLY GSHL---XLE V-EPSV-RDP RDEGTADAWI ERNPSLXRLT GKHPFNSEPP LPRLMHHGFI

                   180                   200                   220                   240
SpNR      TPVPLHYVRN HGAVPRGKWE DWSVEVGGLV RRPRRLTMDE IAGEFSAREL PVTLACCGNR RKEQNMVKQS IGFNWGAAGV 201
OsNR3     TPAALHFVRN HGAVPRGDWS TWTVEVTGLV KRPMRLTVDE LVNGFPAVEV PVTLACSGNR RKEQNMVQQT VGFNFGAAAV 216
OsNR2     TPAALHYVRN HGAVPRGDWS TWTVDVTGLV KRPMRLTMDE LVNGFPAVEI PVTLVCAGNR RKEQNMVQQT VGFNWGAAGV 216
SbNR2     TPAPLHYVRN HGAVPRGDWA TWAVEVTGLV KRPARITMEE LARDFPAVEI PVTLACAGNR RKEQNMVRQT VGFNWGAAGV 212
HvNR2     TPAPLHYVRN HGAVPRGDWA TWTVEVTGLV RRPARLTMDE LANGFPAAEV PATLVCAGNR RKEQNMVQQT VGFNWGAAGV 213
BdNR2     TPAPLHYVRN HGAVPRADWA TWTVEVTGLV RRPARFTMDE LVHEFPAVEI PATLVCAGNR RKEQNMVQQT VGFNWGAAGV 206
SbNR3     TPAPLHYVRN HGPVPRGDWA AWTVEVAGLV RRPARLTMDE LARGFRALEL PVTLVCSSNR RKEQNMARQT LGFNWGPGAV 222
OsNR1     TPAPLHYVRN HGAVPKADWS TWAVEVTGLV KRPARLNMEQ LVTGFEAVEL PVTLVCAGNR RKEQNMVRQT VGFNWGPGAI 189
SbNR1     TPAPLHYVRN HGPVPRADWS TWTVEVTGLV RRPARLTMEQ LVTEFEAVEL PVTLVCAGNR RKEQNMVRQT VGFNWGPGAI 192
HvNR1     TPVPLHYVRN HGAVPKADWS TWTVEVTGLV KRPVKFTMEE LVTGFQAVEF PVTLVCAGNR RKEQNMVRQS SGFNWGPGAI 192
BdNR1     TPAPLHYVRN HGAVPKADWA TWTVEITGLV KRPMKLTMEQ LATEFEAVEF PVTLVCAGNR RKEQNMVRQS VGFNWGPGAV 192
AtNR2     TPVPLHYVRN HGHVPKAQWA EWTVEVTGFV KRPMKFTMDQ LVSEFAYREF AATLVCAGNR RKEQNMVKKS KGFNWGSAGV 215
AtNR1     TPVPLHYVRN HGAVPKANWS DWSIEITGLV KRPAKFTMEE LISEFPSREF PVTLVCAGNR RKEQNMVKQT IGFNWGSAGV 221
NnNR1     TPVQLHYVRN HGPVPQAKWA DWTVEVCGFM NRPAVFTMDQ LVTEFPSREF PVTLVCAGNR RKEQNLVKQT IGFNWGAAGV 207
NnNR2     TPIQLHYVRN HGPVPKAKWE DWTVEVCGLV KQPAVFTMDR LVSEFPSCEF PVTLVCAGNR RKEQNMVKQT IGFNWGAAGI 208
Consensus TPAPLHYVRN HGAVPRADWA TWTVEVTGLV KRPARLTMDE LVTEFPAVEF PVTLVCAGNR RKEQNMVRQT VGFNWGAAGV

                   260                   280                   300                   320
SpNR      STGVWRGARL CDILRRCGAM RRPPTGEAAG GGAPPKMNVC FEGDEDLPGG GG-----SKY GTSLPLEVAL DPANDIILAY 276
OsNR3     STSVWHGARL RDVLRRCGIM PSKG--GA-- ------LNVC FEGAEDLPGG GG-----SKY GTSITRQWAL DPSRDIMLAY 281
OsNR2     STSVWRGARL RDVLRRCGIM PSKG--GA-- ------LNVC FEGAEDLPGG GG-----SKY GTSITRQWAL DPSRDIMLAY 281
SbNR2     STSVWRGARL RDVLRRCGIM PRKGPGGA-- ------LNVC FEGAEDLPGG GGGGGGSTKY GTSVTREWAL DPSRDIMLAY 284
HvNR2     STSVWRGARL RDVLLRCGVM -SKKGQ-A-- ------LNVC FEGAEDLPGG GG-----SKY GTSVSREWAM DPSRDIILAY 278
BdNR2     STSVWRGARL RDVLRRCGVM GARQGGGA-- ------LNVC FEGAEDLPGG GG-----SKY GTSVTREWAL DPSRDIMLAY 273
SbNR3     STSVWRGARL RDVLRRCGGV VDDGDGGA-- ------LYVC FEGDEDLPGG GG-----SKY GTSITRERAL DPTMDVMLAY 289
OsNR1     STSVWRGVRL RDVLRWCGVM GASA--GA-- ------ANVC FEGAEDLPGG GG-----CKY GTSLRREVAM DPAHDVILAY 254
SbNR1     STSVWRGARL RDVLRRCGVM GAAD--GA-- ------ANVC FEGAEDLPGG GG-----SKY GTSLRRGVAM DPARDVILAY 257
HvNR1     STTVWRGVRL RDVLRRCGVM GAGA---A-- ------SNVC FEGAEDLPGG GG-----CKY GTSLRRSVAM DPARDVILAY 256
BdNR1     STSVWRGARL RDVLRRCGVM GRSGDSGA-- ------HNVC FEGAEDLPGG GG-----CKY GTSLRRAVAM DPARDVILAY 259
AtNR2     STSVWRGVPL CDVLRRCGIF SRKG--GA-- ------LNVC FEGSEDLPGG AGTAG--SKY GTSIKKEYAM DPSRDIILAY 283
AtNR1     STSLWKGIPL SEILRRCGIY SRRG--GA-- ------LNVC FEGAEDLPGG GG-----SKY GTSIKKEMAM DPARDIILAY 286
NnNR1     STSVWRGVRL CDVLKRCGIY SRRK--GA-- ------LNVC FEGAEDLPGS GG-----SKY GTSIKKEIAM DPSRDIIIAY 272
NnNR2     STSVWRGIRL CKVLKRCGIY SRRK--GA-- ------LNVC FEGAEDLPGG GG-----SKY GTSIKKEIAM DPSRDIIIAY 273
Consensus STSVWRGARL RDVLRRCGIM GRKG--GA-- ------LNVC FEGAEDLPGG GG-----SKY GTSITREXAM DPSRDIILAY

                   340                   360                   380                   400
SpNR      MHNGELLSPD HGFPVRMIIP GYIGGRMVKW LRRILVTTAE CDSYYHYHDN KVLPSFVDAE LAKADGWWYR QEYMINELNI 356
OsNR3     MQNGEPLLPD HGFPVRAIIP GCTGGRMVKW VKRIIVTTAE SDNYYHYKDN RVFPSHVDAE LANADAWWYK PEYIINELNV 361
OsNR2     MQNGEPLLPD HGFPVRAIIP GCIGGRMVKW VKRIIVTTAE SDNYYHYKDN RVLPSHVDAE LANADAWWYK PEYIINELNV 361
SbNR2     MQNGEPLLPD HGFPVRVIIP GCIGGRMVKW LKRIVVTPAE SDNYYHYKDN RVLPSHVDAE LANAEAWWYK PEYIINELNI 364
HvNR2     AQNGEPLLPD HGYPVRVLIP GCIGGRMVKW VRRIVVTTAE SDNYYHFKDN RVLPSHVDAE LANAEAWWYR PEYIINELNT 358
BdNR2     MQNGEPLLPD HGFPVRVIIP GCIGGRMVKW VKRIIVTTAE SDNYYHFKDN RVLPSHVDAE LANAEGWWYK PEHIINELNT 353
SbNR3     QQNGGPLLPD HGFPVRLIVP GCTAGRMVKW LRRIVVTRAE SDNYYHYRDN RFLPSHVDAK LADAQGWWYK PEYVINEMNT 369
OsNR1     MQNGEPLTPD HGFPVRVIVP GFIGGRMVKW LKRIVVASSE SESYYHYRDN RVLPSHVDAE LANAEAWWYK PEYMINELNI 334
SbNR1     MQNGEPLAPD HGFPVRVIVP GFIGGRMVKW LKRIVVASSE SESYYHYRDN RVLPSHVDAE LANAEAWWYK PEYMINELNI 337
HvNR1     MQNGEPLAPD HGFPVRVIVP GFIGGRMVKW LKRIVVACNE SESYYHYRDN RVLPSHVDAE LANAEAWWYK PECMINELNI 336
BdNR1     MQNGEPLAPD HGFPVRVIVP GFIGGRMVKW LKRIVVACNE SESYYHYRDN RVLPSHVDAE LANAEAWWYK PECMINELNI 339
AtNR2     MQNGEYLTPD HGFPVRIIIP GFIGGRMVKW LKRIIVTTKE SDNFYHFKDN RVLPSLVDAE LADEEGWWYK PEYIINELNI 363
AtNR1     MQNGELLTPD HGFPVRVIVP GFIGGRMVKW LKRIIVTPQE SDSYYHYKDN RVLPSLVDAE LANSEAWWYK PEYIINELNI 366
NnNR1     MQNGELLSPD HGFPIRMIIP GFIGGRMVKW LKRIIVTTEE SQNYYHYKDN RVLPSHVDAE LANAEAWWYK PEYIINELNI 352
NnNR2     MQNGELLSPD HGFPVRMIIP GFIGGRMVKW LKRIIVTTEE SQNYYHYKDN RVLPPHVDAE LANAEAWWYK PEYIINDLNI 353
Consensus MQNGEPLLPD HGFPVRVIIP GFIGGRMVKW LKRIIVTTAE SDNYYHYKDN RVLPSHVDAE LANAEAWWYK PEYIINELNI
```

```
                                          420                   440                   460                   480
SpNR       NSVITTPAHD EILPINSVTS QRFYTMKGYA YAGGGKKVTR VEVTLDGGET WLQCELDLPE RPNKYGKYWC WCFWSVDLEV 436
OsNR3      NSVITAPGHD EILPINGITT QRGYTMKGYA YSGGGKRITR VEVTLDGGET WLVCVLDLPE KPTKYGKHWC WCFWSVEVEV 441
OsNR2      NSVITTPGHD EILPINGITT QRGYTMKGYA YSGGGKRITR VEVTLDGGET WLVCVLDLPE KPTKYGKHWC WCFWSVEVEV 441
SbNR2      NSVITTPGHD EILPINAITT QRGYTMKGYA YSGGGKKVTR VEVTLDGGET WLVCDLDHPE KPNKYGKYWC WCFWSVEVEV 444
HvNR2      NSVITTPGHD EILPINAFTT QRAYTIKGYA YSGGGKKITR VEVTLDGGES WMLCTLDIPE KPNKYGRYWC WCFWSVEIEV 438
BdNR2      NSVITTPGHD EILPINAFTT QRAYTMKGYA YSGGGKKITR VEVTLDGGET WQVCTLDIPE KPNKYGRYWC WCFWSVEVEV 433
SbNR3      NSVITTPGHN DFLPINAITT QRTYTMKGFA YSGGGKKVTR VEVTLDGGQT WLLCVLDHPE KPTKYGRCWC WCFWSIDVEL 449
OsNR1      NSVITTPGHD EVLPINALTT QRPYTMKGYA YSGGGRKVTR VEVTLDGGET WQVCNLDHPE RPTKYGKYWC WCFWSVDVEV 414
SbNR1      NSVITTPGHD EVLPINALTT QRPYTIKGYA YSGGGRKVTR VEVTLDGGET WQVCSLDHPE RPTKYGKYWC WCFWSVDVEV 417
HvNR1      NSVITTPGHD EVLPINALTT QKPYTMKGYA YSGGGRKVTR VEVTLDGGET WQVCDLEHPE RPTKYGKYWC WCFWSVEVEV 416
BdNR1      NSVIATPGHD EVLPINALTT QKPYTMKGYA YSGGGRKVTR VEVTLDGGET WQVCALEHPE RPTKYGKYWC WCFWSVEVEV 419
AtNR2      NSVITTPCHE EILPINAFTT QRPYTLKGYA YSGGGKKVTR VEVTVDGGET WNVCALDHQE KPNKYGKFWC WCFWSLEVEV 443
AtNR1      NSVITTPGHA EILPINAFTT QKPYTLKGYA YSGGGKKVTR VEVTLDGGDT WSVCELDHQE KPNKYGKFWC WCFWSLDVEV 446
NnNR1      NSVITTPCHE EFLPINSTAN QRPYTLRGYA YSGGGKKVTR VEVTLDGGES WLVCALDHPE KPNKYGKYWC WCFWSLDVEM 432
NnNR2      NSVITTPCHQ EILPINSTTT QRSYTLRGYA YSGGGKKVTR VEVTLDGGDS WLVCALDHPE KPNRYGKYWC WCFWSVEVEV 433
Consensus  NSVITTPGHD EILPINAXTT QRPYTMKGYA YSGGGKKVTR VEVTLDGGET WLVCALDHPE KPNKYGKYWC WCFWSVEVEV

                                          500                   520                   540                   560
SpNR       LDLLGAKEIA VRAWDQALNT QPEKLIWNVL GMMNNCWFRV KVNVCRPHRG EIGLVFEHPT KPGNQTGGWM AREKHLEITE 516
OsNR3      LDLLGAKEIA VRAWDQSHNT QPEKLIWNLM GMMNNCWFKV KVNVCRPHKG EIGLVFEHPT QPGNQTGGWM ARQKHLETAE 521
OsNR2      LDLLGAKEIA VRAWDQSHNT QPEKLIWNLM GMMNNCWFKV KVNVCRPHKG EIGLVFEHPT QPGNQTGGWM ARQKHLETAE 521
SbNR2      LDLLGAKEIA VRAWDQSLNT QPEKLIWNLM GMMNNCWFKV KVNVCRPHRG EIGLVFEHPT QPGNQSGGWM ARQKHLETAE 524
HvNR2      LDLLGAKEVA VRAWDQTHNT QPEKLIWNLM GMMNNCWFKI KVNVCRPHKG EIGLVFEHPT QPGNQTGGWM ARQKHLETAE 518
BdNR2      LDLLGAKEVA VRAWDQTHNT QPEKLIWNLM GMMNNCWFKV KVNVCRPHKG EIGLVFEHPT QPGNQTGGWM ARQKHLETAE 513
SbNR3      ADLLACKEIA VRAWDQSLNT QPEFLTWNLL GMMTNCWFRV KVNVCRPSNG EIGLAFEHPV QPGNQPGGWM AQQKHIESAE 529
OsNR1      LELLAAKEIA VRAWDESLNT QPEKLIWNLM GMMNNCWFRV KTKTCRPHKG EIGLVFEHPT QPGNQAGGWM ARQKHLETS- 493
SbNR1      LDVLGAKEIA VRAWDEAMNT QPEKLIWNLM GMMNNCWFRV KINACRPHKG EIGMVFEHPA QPGNQPGGWM ARQKHLETS- 496
HvNR1      LELLGAKEMA VRAWDEALNT QPERLIWNLM GMMNNCWFRV KINVCRPHKG EIGLVFDHPT QPGNQSGGWM ARQKHIETS- 495
BdNR1      LELLGAKEMA VRAWDEAMNT QPEKLIWNLM GMMNNCWFRV KINVCRPHKG EIGLVFEHPT QPGNQPGGWM ARQKHMDTST 499
AtNR2      LDLLSAKEIA VRAWDETLNT QPEKMIWNLM GMMNNCWFRV KTNVCKPHKG EIGIVFEHPT LPGNESGGWM AKERHLEKSA 523
AtNR1      LDLLSAKDVA VRAWDESFNT QPDKLIWNLM GMMNNCWFRI RTNVCKPHRG EIGIVFEHPT RPGNQSGGWM AKERQLEISS 526
NnNR1      LDLLGANEIA VRAWDESLNT QPEELIWNVM GMMNNCWFRV KMNVCKRHKG EIGIVFEHPT QPANQPGGWM DRQKHLD-SA 511
NnNR2      MYLLGAKEIA VRAWDETLNT QPEKLIWNVM GMMNNCWFRV KMNVCKPHKG EIGIVFEHPT QPGNQPGGWM ARQKHLE-SA 512
Consensus  LDLLGAKEIA VRAWDESLNT QPEKLIWNLM GMMNNCWFRV KVNVCRPHKG EIGLVFEHPT QPGNQXGGWM ARQKHLETSE

                                          580                   600                   620                   640
SpNR       GQ------S- LKKSISSPFM NTAA---KHY SMSEVRRHST RDSAWIVVHG HVYDCTSFLK DHPGGADSIL INAGTDCTED 586
OsNR3      AAA----PGL K-RSTSTPFM NT-T-DGKQF TMSEVRKHSS QDSAWIVVHG HVYDCTAFLK DHPGGADSIL INAGTDCTEE 594
OsNR2      AAA----PGL K-RSTSTPFM NT-T-DGKQF TMSEVRKHSS QDSAWIVVHG HVYDCTAFLK DHPGGADSIL INAGTDCTEE 594
SbNR2      AAA----PGL K-RSTSTPFM STIS-DGKQF TMSEVRRHAS QESAWIVVHG HVYDCTKFLK DHPGGADSIL INAGTDCTEE 598
HvNR2      AAA----PGL K-RSTSTPFM NTAG-D-KQF TMSEVRKHGS KESAWIVVHG HVYDCTAFLK DHPGGADSIL INAGSDCTEE 591
BdNR2      AAA----PGL K-RSTSTPFM NTTG-D-KQF TMSEVRKHGS QESAWIVVHG HVYDCTAFLK DHPGGADSIL INAGTDCTEE 586
SbNR3      AAGAASPPAL PPRSTSAATS TTNT-ASNQF TMSEVRKHAS KDSAWIVVHG HVYDCTEYLK DHPGGADSIL INAGTDCTEE 608
OsNR1      -ESAV---ST LKRSTSTPFL NTATTQ---Y TMSEVRRHTT PESAWIIVHG HVYDCTGFLK DHPGGADSIM INAGTDCTEE 566
SbNR1      -ESAQ---GT LKKSTSTPFM NTATTQ---Y TMSEVRRHTS PDSAWIIVHG HIYDCTGFLK DHPGGADSIL INAGTDCTEE 569
HvNR1      -ETTQ---GT LKRSTSTPFM STASAQ---F TMSEVRRHAS KDSAWIVVHG HVYDCTAFLK DHPGGADSIL INAGSDCTEE 568
BdNR1      SETSQ---GT LKRSTSTPFM AVAAANSRYY AMSEVRRHAS RESAWIVVHG HVYDCTGFLK DHPGGADSIL INAGTDCTEE 576
AtNR2      DA-----PPS LKKSVSTPFM NTTA---KMY SMSEVKKHNS ADSCWIIVHG HIYDCTRFLM DHPGGSDSIL INAGTDCTEE 595
AtNR1      ES-----NNT LKKSVSSPFM NTAS---KMY SISEVRKHNT ADSAWIIVHG HIYDCTRFLK DHPGGTDSIL INAGTDCTEE 598
NnNR1      ES-----NPI MKKSVSSPFM NISS---KTF SMSEVKKHNS AESAWIVVHG KVYDCTPFLK DHPGGADSIL INAGGDCTEE 583
NnNR2      ES-----NQT MKKSVSSPFM NTSS---KTF SMPEVNKHNS AESAWIVVHG HVYDCTGFLK DHPGGVDSIL INAGSDCTEE 584
Consensus  AAA----PGX LKRSTSTPFM NTAX-D-KQF TMSEVRKHXS XDSAWIVVHG HVYDCTAFLK DHPGGADSIL INAGTDCTEE

                                          660                   680                   700                   720
SpNR       FDAIHSEKAK SMLETYRVGE LITTG--YLS DA-------S NHGANNLAL- --LSSIAEA- ---APSPRRA VALVPRE-KI 649
OsNR3      FDAIHSDKAK ALLDTYRIGE LITTGAGYSS D-------NS VHGASN-LSQ --LAPIREAI K-APAP---V ALSSPRD-KV 659
OsNR2      FDAIHSDKAK ALLDTYRIGE LITTGAGYSS D-------NS VHGASN-LSQ --LAPIREAI K-APAP---V ALSSPRD-KV 659
SbNR2      FDAIHSDKAK ALLDTYRIGE LITTGTGYSS D-------NS VHGGSAVLSN --LAPIREAV R-APAP---V ALSNPRE-KI 664
HvNR2      FDAIHSDKAK ALLDTYRIGE LITTGTGYNS D-------NS VHGGSS-LSH --LAPIREAT KVAGAP---I ALSSPRE-KV 657
BdNR2      FDAIHSDKAK ALLDTYRVGE LITTGTGYNS D-------NS VHGGSN-LSH --LAPIREAT K-ALAP---I ALSSPRE-KV 651
SbNR3      FDAIHSDKAK DLLGAYRIGD LLVTAAGTEQ A-------SH SH-----LEH ---API---- -GGPAPPVVV ALSNPREEKV 668
OsNR1      FDAIHSDKAR GLLEMYRIGE LIVTGSDYS- P-------QS SSADLTSIVE SPTAAAAPAV -----PVSTV ALSNPRE-KV 632
SbNR1      FDAIHSDKAR GLLDLYRVGE LVVTGSDYS- P-------QN SHADLRAIDE APAAAAPAAQ -----SVSTV ALSNPRE-KV 635
HvNR1      FDAIHSAKAR GLLEMYRVGE LIVTGNDYS- P-------QS SNADLAAIVE APAVVVPRL- -----PASAV ALANPRE-KV 633
BdNR1      FDAIHSAKAR GLLEMYRVGE LIATGADYSS P-------QS SHADLNAIAE PPPTVVPQQV IISSSSSSAV ALANPRE-KV 648
AtNR2      FEAIHSDKAK KMLEDYRIGE LITTG--YSS DSSS--PNNS VHGSSAVFSL --LAPIGEA- ----TPVRNL ALVNPRA-KV 663
AtNR1      FEAIHSDKAK KLLEDYRIGE LITTG--YDS ---S--PNVS VHGASNFGPL --LAPIKEL- ----TPQKNI ALVNPRE-KI 663
NnNR1      FDAIHSDKAR KLLEDYRIGE LITFG--YTS DSSSS-PNNS VHGASNL-TL --LAPIKEV- ---STVTINV ALI-PRE-KI 651
NnNR2      FDAIHSDKAK KLLEDYRLGQ LIATA--YNS DSSSSSPNNS VHGASDL-TH --LDPIKEV- ----TVTRNV ALI-PRE-KI 652
Consensus  FDAIHSDKAK ALLETYRIGE LITTG-GYSS D-------NS VHGASN-LSX --LAPIREA- ----AP---V ALSNPRE-KV

                                          740                   760                   780                   800
SpNR       QCRLVSKTSL SHDVRRFRFA LPAADQVLGL PVGKHIFLSA LVDGKPCMRA YTPTSTDEEI GHFELVVKVY FKDVHPKFPR 729
OsNR3      PCQLVDKKEL SRDVRLFRFA LPSSDQVLGL PVGKHIFVCA SIEGKLCMRA YTPTSMVDEV GHFDLLIKVY FKNEHPKFPD 739
OsNR2      PCQLVDKKEL SRDVRLFRFA LPSSDQVLGL PVGKHIFVCA SIEGKLCMRA YTPTSMVDEV GHFDLLIKVY FKNEHPKFPD 739
SbNR2      HCRLVGKKEL SRDVRLFRFA LPSSDQVLGL PIGKHIFVCA TIDGKLCMRA YTPTSMVDEI GHFDLLVKVY FKNEHPKFPA 744
HvNR2      PCRLVDKKEL SHDVRLFRFA LPSSDQVLGL PVGKHIFVCA TIDGKLCMRA YTPTSMVDEI GQFELLVKVY FRDEHPKFPN 737
BdNR2      PCRLVDKKEL SHDVRLFRFA LPSSDQVLGL PVGKHIFVCA NIEGKLCMRA YTPTSMVDEI GQFELLVKIY FKDEHPKFPN 731
SbNR3      PCRLVAKTVL SRDVRLFRFA LPSSGQVLGL PVGKHIFVCA SIDGKLCMRA YTPTSSVDEV GHFDLLVKVY FKNENPKFPD 748
OsNR1      KCRLMDKKSL SYNVRLFRFA LPSPDQKLGL PVGKHVYVCA SIGGKLCMRA YTPTSSVDEV GYIELLIKIY FKGEDPKFPD 712
SbNR1      RCRLVDKKSL SYNVRLFRFA LPSPDQKLGL PVGRHVYVCA SIDGKLCMRA YTPTSPVDEV GHIELLIKIY FKDEDPKYPN 715
HvNR1      RCRLVDKKSM SHNVRLFRFA LPSPHQKLGL PVGKHVYVCA STGGKLCMRA YTPTSSVEEV GHVELLIKIY FKDEDPKFPA 713
BdNR1      RCKLVGKKSV SRNVRLFRLA LPSPDQKLGL PVGKHVYVCA TTGGKLCMRA YTPTSSPEES GHVELLIKIY FKGEDPKFPG 728
AtNR2      PVQLVEKTSI SHDVRKFRFA LPVEDMVLGL PVGKHIFLCA TINDKLCLRA YTPSSTVDVV GYFELVVKIY FGGVHPRFPN 743
AtNR1      PVRLIEKTSI SHDVRKFRFA LPSEDQQLGL PVGKHVFVCA NINDKLCLRA YTPTSAIDAV GHIDLVVKVY FKDVHPRFPN 743
NnNR1      PCKLICKTSI SHDVRLFRFA LPSKDQVLGL PVGKHIFLCA TIDNKLCIRA YTPTSSVDDV GYFDLVVKVY FKGMNPKFPN 731
NnNR2      ACKLVSKISI SHDVRLFRFT LPSNDQVLGL PVGKHIFLCA TIDGKLCMRA YTPTSSVDEV GYFDLVIKVY FKGINPKFPN 732
Consensus  PCRLVDKKSL SHDVRLFRFA LPSSDQVLGL PVGKHIFVCA XIDGKLCMRA YTPTSSVDEV GHFELLVKVY FKDEHPKFPN
```

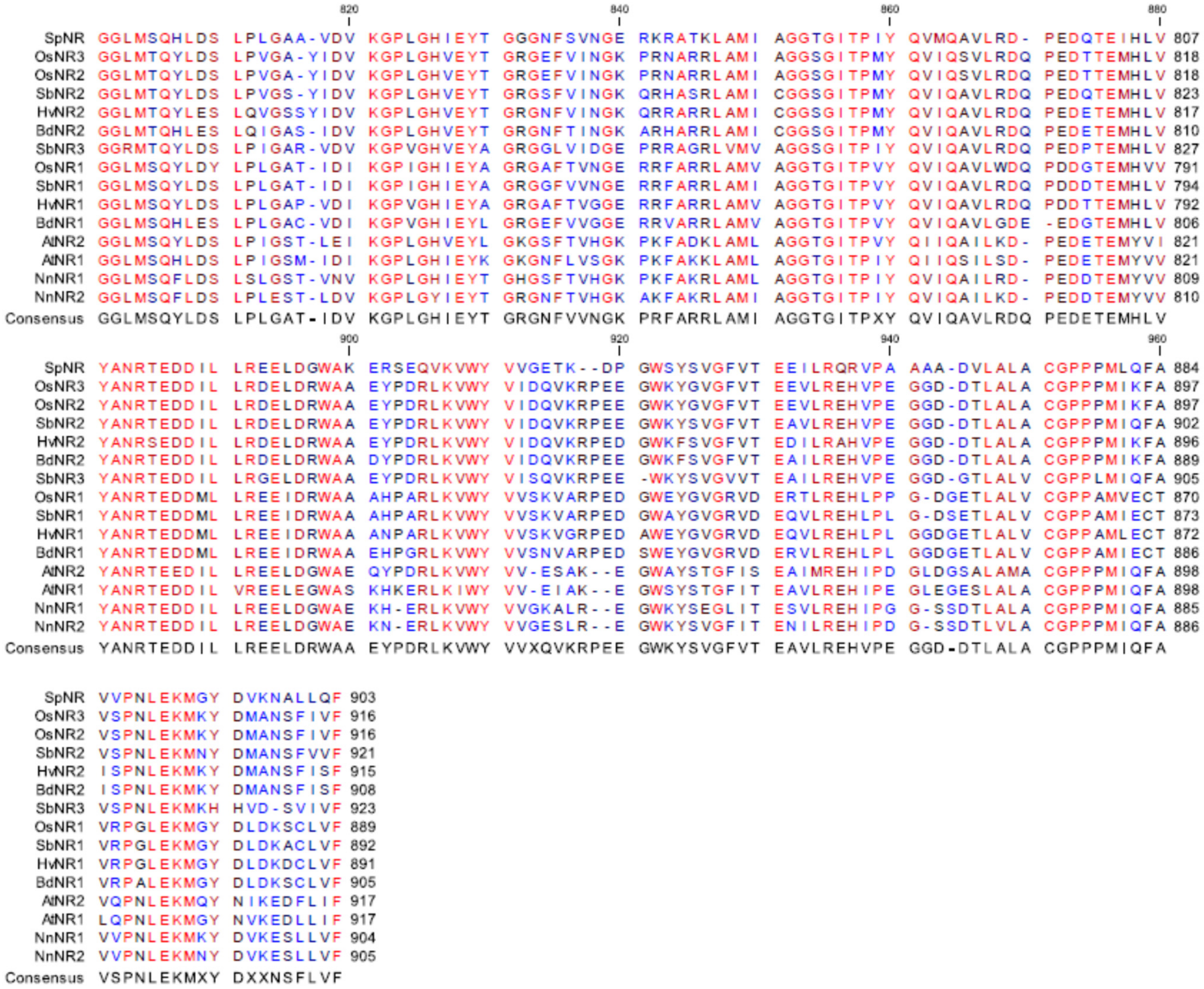

B

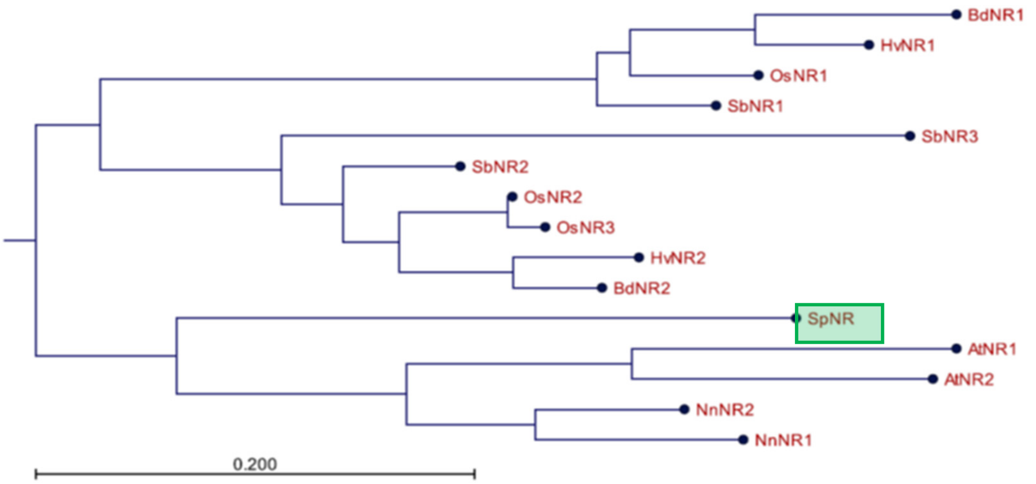

Figure S5. Sequence alignment of NR protein from *S. polyrhiza* with other representative species (**A**) and the resulting proteins phylogenetic tree (**B**). AtNR1 – *A. thaliana,* NP_177899.1; AtNR2 - *A. thaliana,* NP_174901.1; BdNR1 – *B. distachyon,* XP_003570548.1; BdNR2 - *B. distachyon,* XP_003574607.1; HvNR1 – *H. vulgare,* KAE8814139; HvNR2 - *H. vulgare,* KAE8794287; NnNR1 - *N. nucifera,* XP_010246478; NnNR2 - *N. nucifera,* XP_010245911; OsNR1 - *O. sativa,* XP_015622710.1; OsNR2 - *O. sativa,* XP_015650300.1; OsNR3 - *O. sativa,* XP_015650643.1; SbNR1 – *S. bicolor,* XP_002454625.1; SbNR2 – *S. bicolor,* XP_002444490.1; SbNR3 – *S. bicolor,* XP_002454083.1, SpNR - *S. polyrhiza* (sequence translated from Acc. OL421561)

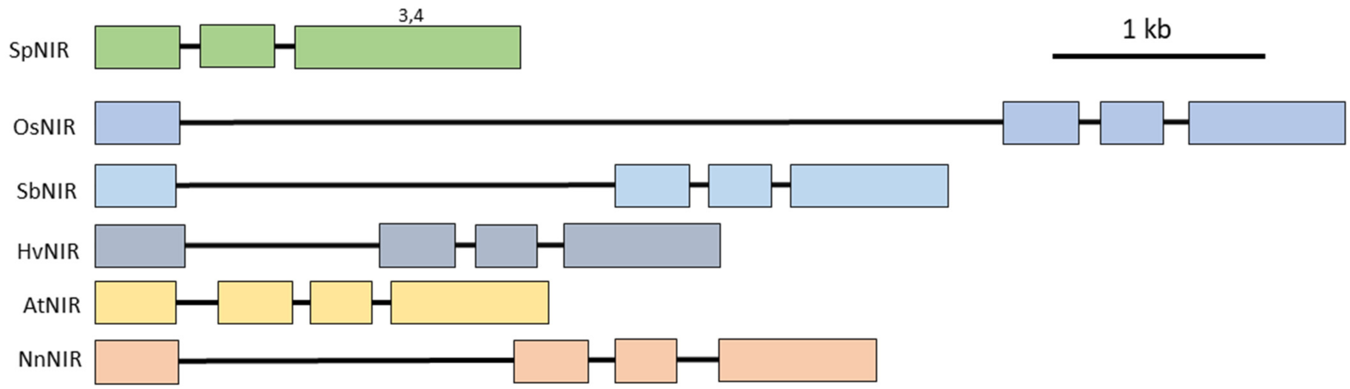

Figure S6. Comparison of the exon-intron structures between *NIR* genes of *S. polyrhiza* and other representative plant species. *AtNIR* – *A. thaliana*, At2g15620; *NnNIR - N. nucifera,* LOC104601782; *OsNIR - O. sativa,* LOC4326014; *SbNIR - S. bicolor,* LOC8075200, *SpNIR – S. polyrhiza* CP019094, the 18th chromosome, position from 2760613 to 2758617. Coding sequences are colored boxes. Introns are black lines. The bar corresponds to 1kb of DNA sequence.

A

```
                                       20                             40                             60                             80
SpNIR      -MSSSAVH-- LSMPSWQAVR RQRR----RG PAVLATATPT AAPAAPQPPA PPEDGA-EVS RLEPRVEQRE GGYWVLKEKF 72
OsNIR      MASSASLQRF L--PPYPHAA ASRC----RP PGVRARPVQS STVSAPSSST PAADEAVSAE RLEPRVEQRE GRYWVLKEKY 74
SbNIR      MASSASLQRF LPPSSHAATA SSSR----RR TG-RARAAVS MPPSSP---- -PATGEVPAE RLEPRVEERE GGYWVLKEKY 70
HvNIR      MASSASLQSF LP-PSAHAAT SSSRLRPSRA RPVQCAAV-- SAPSSSSSSA SPSASAVPSE RLEPRVEQRE GGYWVLKEKY 77
BdNIR      MASSASLQRF LPPSAHSTPS SSQRGRRARR PVLQCSAVSA PSSSSPAPAA ----GAVAAE RLEPRVEQRE GGYWVLKEKY 76
AtNIR      -MTSFSLTF- -TSPLLPSSS TKPK----R- -SVLVAAAQT TAPAE----- --STASVDAD RLEPRVELKD G-FFILKEKF 63
NnNIR      -MSSSFRFL APSVPSSSSM RRTR----RG ARAPAAATPS QAEA------ PSVAKEVDGS RLEPRVEERE G-YWVLKEKF 68
Consensus  MASSASLQRF LPXPSHXAXX SSXR----RX PGVXAAAVXS SAPSSP---A PPXXGAVXAE RLEPRVEQRE GGYWVLKEKY

                                      100                            120                            140                            160
SpNIR      RPGINPQEKV KLEKEPMALF MEGGIRDLAA TPLEDIEASK LSKDDIDVRL KWLGLFHRRK HQYGRFMMRL KLPNGVTTSK 152
OsNIR      RTGLNPQEKV KLGKEPMSLF MEGGIKELAK MPMEEIEADK LSKEDIDVRL KWLGLFHRRK HQYGRFMMRL KLPNGVTTSE 154
SbNIR      RTGLNPQEKV KLEKEPMGLF MEDGIKDLAK IPMEEIDAAK LTKDDVDVRL KWLGLFHRRK HQYGRFMMRL KLPNGVTTSE 150
HvNIR      RTSLNPQEKV KLGKEPMALF TEGGINDLAK LPMEQIDADK LTKEDVDVRL KWLGLFHRRK QQYGRFMMRL KLPNGVTTSE 157
BdNIR      RTGLNPQEKV KLGKEPMALF TESGIRDLAK IPMAEIDADK LAKEDVDVRL KWLGLFHRRK QQYGRFMMRL KLPNGVTTSE 156
AtNIR      RKGINPQEKV KIEREPMKLF MENGIEELAK KSMEELDSEK SSKDDIDVRL KWLGLFHRRK HQYGKFMMRL KLPNGVTTSA 143
NnNIR      RQGINPQEKV KLEREPMKLF MEGGINELAK TPLQEIEKSK LTKDDIDVRL KWLGLFHRRK HQYGRFMMRL KLPNGVTTSE 148
Consensus  RTGLNPQEKV KLEKEPMALF MEGGIXDLAK XPMEEIDADK LXKDDIDVRL KWLGLFHRRK HQYGRFMMRL KLPNGVTTSE

                                      180                            200                            220                            240
SpNIR      QIRYLAGVIE AYGEDGCADV TTRQNWQIRG VRLPDVPAIL DGLAGVGLSS LQSGMDNVRN PVGNPLAGFD PHEIVDTRPY 232
OsNIR      QTRYLASVIE AYGKEGCADV TTRQNWQIRG VTLPDVPAIL DGLNAVGLTS LQSGMDNVRN PVGNPLAGID PDEIVDTRSY 234
SbNIR      QTRYLASVIE AYGADGCADV TTRQNWQIRG VTLPDVPAIL DGLRAVGLTS LQSGMDNVRN PVGNPLAGVD PHEIVDTRPY 230
HvNIR      QTRYLASVID KYGEEGCADV TTRQNWQIRG VTLPDVPEIL DGLRSVGLTS LQSGMDNVRN PVGSPLAGID PLEIVDTRPY 237
BdNIR      QTRYLASVID KYGEEGCADV TTRQNWQIRG VTLPDVPDIL DGLHSVGLTS LQSGMDNVRN PVGNPLAGID PDEIVDTRPY 236
AtNIR      QTRYLASVIR KYGEDGCADV TTRQNWQIRG VVLPDVPEIL KGLASVGLTS LQSGMDNVRN PVGNPIAGID PEEIVDTRPY 223
NnNIR      QTRCLASVIR KYGKEGCGDV TTRQNWQIRG VELPDVPEIM EDLVRVGLTS LQSGMDNVRN PVGNPLAGID PHEIVDTRPY 228
Consensus  QTRYLASVIE KYGEEGCADV TTRQNWQIRG VTLPDVPXIL DGLXSVGLTS LQSGMDNVRN PVGNPLAGID PHEIVDTRPY

                                      260                            280                            300                            320
SpNIR      ANLLSQFITG NDRGNPAVTN LPRKWNACVV GSHDLYEHPH INDLAYMPAT KEGRFGFNLL VGGFISPKRW AEAVPLDAWV 312
OsNIR      TNLLSSYITS NFQGNPTITN LPRKWNVCVI GSHDLYEHPH INDLAYMPAV KGGKFGFNLL VGGFISPKRW EEALPLDAWV 314
SbNIR      TNLLSSYITN NSQGNTTITN LPRKWNVCVI GSHDLYEHPH INDLAYMPAV KDGKFGFNLL VGGFISPKRW AEALPLDAWV 310
HvNIR      TNLLSSYITN NSEGNLAITN LPRKWNVCVI GTHDLYEHPH INDLAYMPAE KDGKFGFNLL VGGFISPKRW GEALPLDAWV 317
BdNIR      TNLLSSYITN NSQGNLAVTN LPRKWNVCVI GSHDLYEHPH INDLAYMPAE KDGKFGFNLL VGGFISPKRW GEALPLDAWV 316
AtNIR      TNLLSQFITA NSQGNPDFTN LPRKWNVCVV GTHDLYEHPH INDLAYMPAN KDGRFGFNLL VGGFFSPKRC EEAIPLDAWV 303
NnNIR      TNLLSHFITG NSGGNPAVTN LPRKWNVCVV GSHDLFEHPH INDLAYMPAT KDGRFGFNLL VGGFFSPKRC AEAVPLDAWV 308
Consensus  TNLLSSYITN NSQGNPAXTN LPRKWNVCVI GSHDLYEHPH INDLAYMPAX KDGKFGFNLL VGGFISPKRW AEALPLDAWV

                                      340                            360                            380                            400
SpNIR      PGEDVVPVCR AVLEAYRDLG TRGNRQKTRM MWLIDELGIE AFRAEVERRM PQGVLPRGAS PAEELVKKDW LRRDYLGVHP 392
OsNIR      PGDDIIPVCK AVLEAYRDLG TRGNRQKTRM MWLIDELGME AFRSEVEKRM PNGVLERAAP --EDLIDKKW QRRDYLGVHP 392
SbNIR      AGDDVVPVCK AILEAYRDLG FRGNRQKTRM MWLIDELGME VFRSEVEKRM PNGVLERAA- -AEDLVDKTW ERRDYLGVHP 388
HvNIR      PGDDIIPVCK AVLEAFRDLG TRGNRQKTRM MWLIDELGME AFRSEIEKRM PNGVLERAAP --EDLIDKKW ERRDYLGVHP 395
BdNIR      PGDDIIPVCK AILEAYRDLG TRGNRQKTRM MWLIDELGME VFRSEIEKRM PGGVLERAA- -AEDMIDKAW ERRDYLGVHP 394
AtNIR      PADDVLPLCK AVLEAYRDLG TRGNRQKTRM MWLIDELGVE GFRTEVEKRM PNGKLERGS- -SEDLVNKQW ERRDYFGVNP 381
NnNIR      PADDVVPVCK AVLEAYRDLG TRGNRQKTRM MWLIDELGVE GFRAEVVKRM PGQELERSS- -EEDLVQKQW ERRDYLGVHP 386
Consensus  PGDDVXPVCK AVLEAYRDLG TRGNRQKTRM MWLIDELGME AFRSEVEKRM PNGVLERAA- -AEDLVDKXW ERRDYLGVHP

                                      420                            440                            460                            480
SpNIR      QKQQGLCFVG LHVPVGRVQA SDMFELARLA DEYGTGELRL TVEQNVVIPN VPEGRVAALL EEPLLQERFL PEPSALMRGL 472
OsNIR      QKQEGMSYVG LHVPVGRVQA ADMFELARLA DEYGSGELRL TVEQNIVIPN VKNEKVEALL SEPLLQ-KFS PQPSLLLKGL 471
SbNIR      QKQEGLSYVG LHVPVGRLQA ADMFELARLA DEYGTGELRL TVEQNIVLPN VSNERVDALL AEPLLRQRFS PEPSLLLKGL 468
HvNIR      QKQEGLSFVG LHVPVGRLQA ADMFELARLA DEYGSGELRL TVEQNIVLPN VKNEKVDALL AEPLL-HKFS AHPSLLMKGL 474
BdNIR      QKQQGLSFVG LHVPVGRVQA ADMYSLARLA DEYGSGELRL TVEQNIVLPN VPNDKIPSLL AEPLLQ-KFS AQPSLLMKGL 473
AtNIR      QKQEGLSFVG LHVPVGRLQA DDMDELARLA DTYGSGELRL TVEQNIIIPN VETSKTEALL QEPFLKNRFS PEPSILMKGL 461
NnNIR      QKQEGFSFVG LHVPVGRVQG EEMDELARLA DEYGSGELRL TVEQNVVIPN VENSKLEALL KEPLLRNRFS PEPSLLMKGL 466
Consensus  QKQEGLSFVG LHVPVGRVQA ADMFELARLA DEYGSGELRL TVEQNIVIPN VXNEKVEALL AEPLLQNRFS PEPSLLMKGL

                                      500                            520                            540                            560
SpNIR      VACTGNQFCG QAIIETKARA VRVAEETERR VALPAGKSVR MHWTGCPNSC AQVQVADIGF MGCMARDAAG KVCEAVDIYL 552
OsNIR      VACTGNQFCG QAIIETKQRA LLVTSQVEKL VSVP--RAVR MHWTGCPNSC GQVQVADIGF MGCLTKDSAG KIVEAADIFV 549
SbNIR      VACTGNQFCG QAIIETKARA LQVTREVEKR VSVP--RPVR MHWTGCPNSC GQVQVADIGF MGCLTKDSDG KIVEAADIFV 546
HvNIR      VACTGNQFCG QAIIETKARA LQVTRDVEAR VSVP--KAVR MHWTGCPNSC AQVQVADIGF MGCLTKNSSG KIVEAADIFV 552
BdNIR      VACTGNQFCG QAIIETKARA LRVTRDVERR VSVP--RAVR MHWTGCPNSC AQVQVADIGF MGCLTKNGSG KIVEAADIFV 551
AtNIR      VACTGSQFCG QAIIETKLRA LKVTEEVERL VSVP--RPIR MHWTGCPNTC GQVQVADIGF MGCLTRGEEG KPVEGADVYV 539
NnNIR      VACTGNQFCG QAIIETKARA LKVTEEVERR VAVT--RPVR MHWTGCPNSC GQVQVADIGF MGCMTRDENG KACEGVDVFL 544
Consensus  VACTGNQFCG QAIIETKARA LXVTXEVERR VSVP--RXVR MHWTGCPNSC GQVQVADIGF MGCLTKDSXG KIVEAADIFV

                                      580                            600
SpNIR      GGRVGSDSHL GSVYKKGVPC KDLVPVLVDI LVERFGAVPR QREDDDEEEE ER 604
OsNIR      GGRVGSDSHL AGAYKKSVPC DELAPIVADI LVERFGAVRR EREEDEE--- -- 596
SbNIR      GGRVGSDSHL ADVYKKSVPC KDLVPIVADL LVERFGAVPR EREEDEE--- -- 593
HvNIR      GGRIGSDSHL TGVYKKAVPC EDLVPIVADL LVERFGAVPR EREEDEE--- -- 599
BdNIR      GGRVGSDSHL TGVWKKAVPC EDLVPVVADL LVERFGAVPR EREEDEE--- -- 598
AtNIR      GGRIGSDSHI GEIYKKGVRV TELVPLVAEI LIKEFGAVPR EREENED--- -- 586
NnNIR      GGRIGSDSHL GDVYKKSVPC KDLVPLVVDI LINHFGAVPR DREEEGED-- -- 592
Consensus  GGRVGSDSHL GGVYKKSVPC KDLVPIVADI LVERFGAVPR EREEDEE--- --
```

B

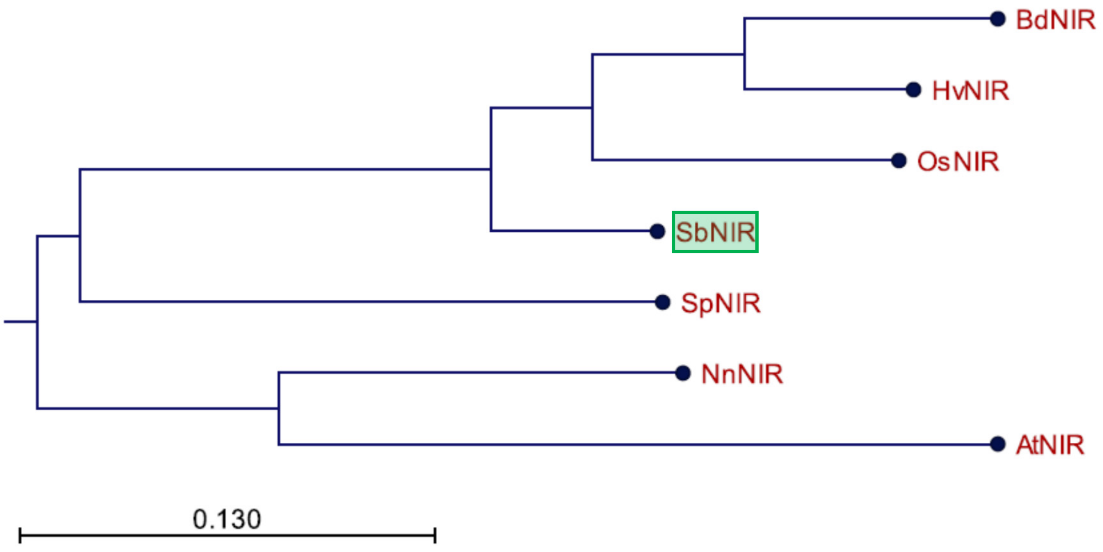

Figure S7. Sequence alignment of NIR protein from *S. polyrhiza* with other representative species (**A**) and the resulted phylogenetic tree (**B**).; AtNIR – *A. thaliana,* NP_179164; BdNIR - *B. distachyon*, XP_003570568; HvNIR - *H. vulgare*, BAW00383; NnNIR - *N. nucifera,* XP_010263547; OsNIR - *O. sativa,* XP_015641702.1; Sb NIR - *S. bicolor*, XP_002454602; SpNIR - *S. polyrhiza* (sequence translated from Acc. OL421562)

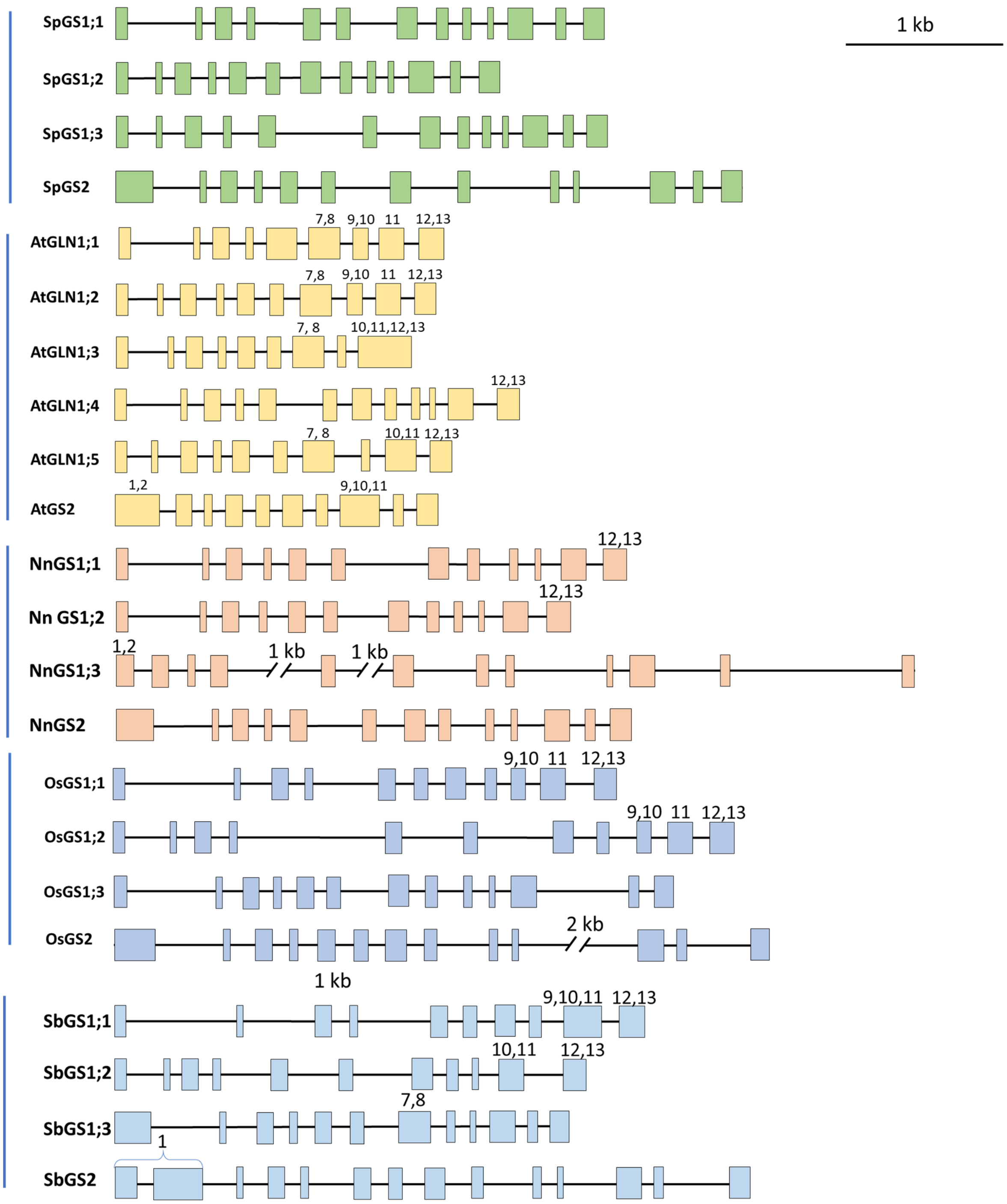

Figure S8. Comparison of the exon-intron structures between *GS* genes of *S. polyrhiza* and other representative plant species. *AtGln1;1* – *A. thaliana,* At5g37600); *AtGln1;2* - *A. thaliana,* At1G66200; *AtGln1;3* - *A. thaliana,* At3g17820; *AtGln1;4* - *A. thaliana,,* At5g16570; *AtGln1;5* - *A. thaliana,* At1g48470; *AtGS2* - *A. thaliana,,* At5g35630; *NnGS1;1* - *N. nucifera,* LOC104607411; *NnGS1;2* - *N. nucifera,* LOC104607404; *NnGS1;3* - *N. nucifera,* LOC104592454; *NnGS2* - *N. nucifera,* LOC104596403, *OsGS1;1* - *O. sativa,* LOC4330649, *OsGS1;2* - *O. sativa,* LOC4332108, *OsGS1;3* - *O. sativa,* LOC4333896, *OsGS2* - *O. sativa,* LOC4337272, *SbGS1;1* - *S. bicolor,* LOC110434374, *SbGS1;2* - *S. bicolor,* LOC8056868, *SbGS1;3* - *S. bicolor,* LOC110431761, *SbGS2* - *S. bicolor,* LOC110436381, *SpGS1;1* – *S. polyrhiza* CP019094, the 7th chromosome, position from 6306673 to 6309664, *SpGS1;2* – *S. polyrhiza* CP019094, the 18th chromosome, position from 1351512 to 1353850, *SpGS1;3* – *S. polyrhiza* CP019094, the 14th chromosome, position from 1601445 to 1604468, *SpGS2* – *S. polyrhiza* CP019094, the 1st chromosome, position from 4942769 to 4946754. Coding sequences are represented by colored boxes, introns are black lines. The bar corresponds to 1kb of DNA sequence.

A

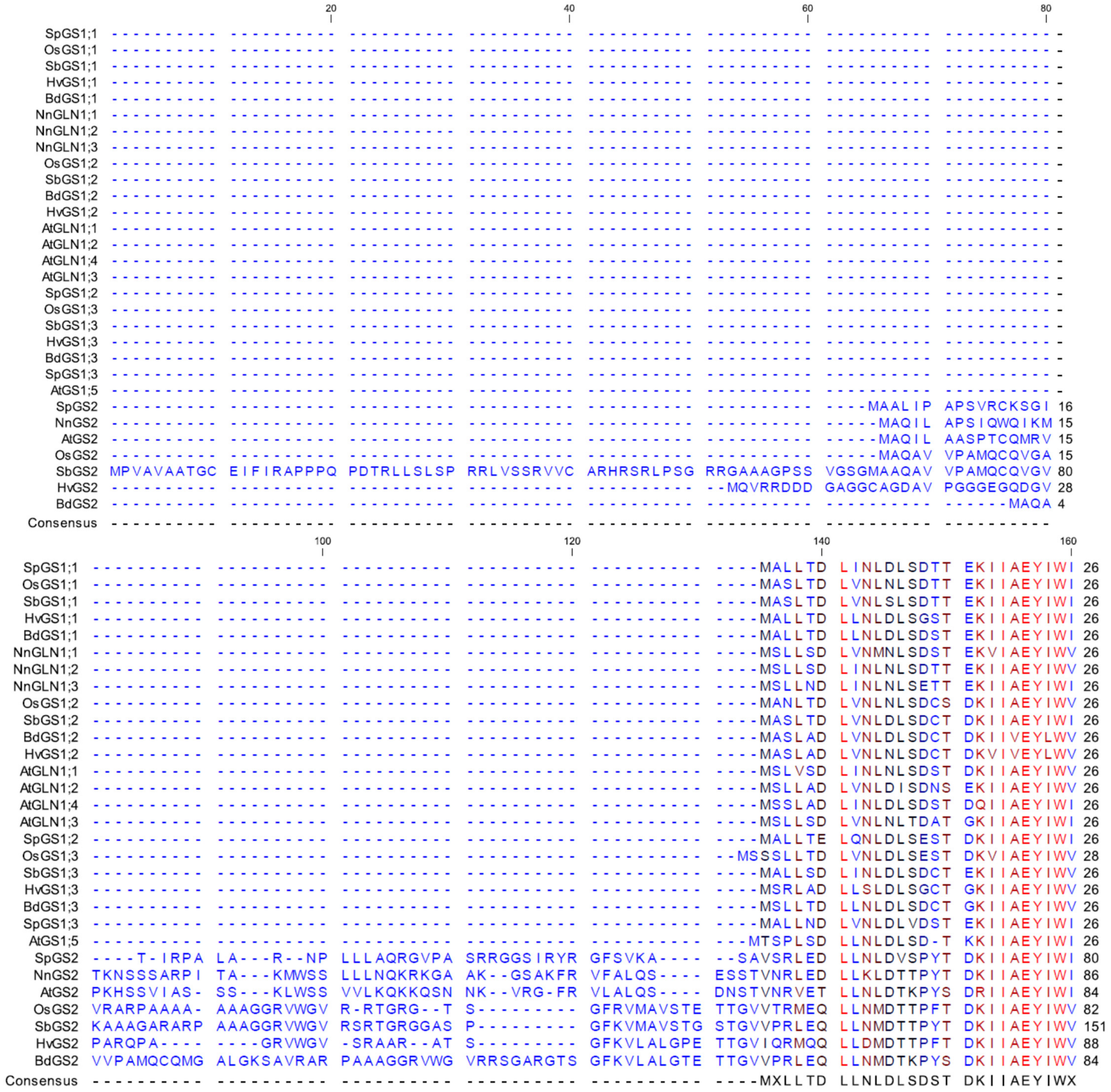
20 40 60 80
SpGS1;1 ---------- ---------- ---------- ---------- ---------- ---------- ---------- ---------- -
OsGS1;1 ---------- ---------- ---------- ---------- ---------- ---------- ---------- ---------- -
SbGS1;1 ---------- ---------- ---------- ---------- ---------- ---------- ---------- ---------- -
HvGS1;1 ---------- ---------- ---------- ---------- ---------- ---------- ---------- ---------- -
BdGS1;1 ---------- ---------- ---------- ---------- ---------- ---------- ---------- ---------- -
NnGLN1;1 ---------- ---------- ---------- ---------- ---------- ---------- ---------- ---------- -
NnGLN1;2 ---------- ---------- ---------- ---------- ---------- ---------- ---------- ---------- -
NnGLN1;3 ---------- ---------- ---------- ---------- ---------- ---------- ---------- ---------- -
OsGS1;2 ---------- ---------- ---------- ---------- ---------- ---------- ---------- ---------- -
SbGS1;2 ---------- ---------- ---------- ---------- ---------- ---------- ---------- ---------- -
BdGS1;2 ---------- ---------- ---------- ---------- ---------- ---------- ---------- ---------- -
HvGS1;2 ---------- ---------- ---------- ---------- ---------- ---------- ---------- ---------- -
AtGLN1;1 ---------- ---------- ---------- ---------- ---------- ---------- ---------- ---------- -
AtGLN1;2 ---------- ---------- ---------- ---------- ---------- ---------- ---------- ---------- -
AtGLN1;4 ---------- ---------- ---------- ---------- ---------- ---------- ---------- ---------- -
AtGLN1;3 ---------- ---------- ---------- ---------- ---------- ---------- ---------- ---------- -
SpGS1;2 ---------- ---------- ---------- ---------- ---------- ---------- ---------- ---------- -
OsGS1;3 ---------- ---------- ---------- ---------- ---------- ---------- ---------- ---------- -
SbGS1;3 ---------- ---------- ---------- ---------- ---------- ---------- ---------- ---------- -
HvGS1;3 ---------- ---------- ---------- ---------- ---------- ---------- ---------- ---------- -
BdGS1;3 ---------- ---------- ---------- ---------- ---------- ---------- ---------- ---------- -
SpGS1;3 ---------- ---------- ---------- ---------- ---------- ---------- ---------- ---------- -
AtGS1;5 ---------- ---------- ---------- ---------- ---------- ---------- ---------- ---------- -
SpGS2 ---------- ---------- ---------- ---------- ---------- ---------- ----MAALIP APSVRCKSGI 16
NnGS2 ---------- ---------- ---------- ---------- ---------- ---------- -----MAQIL APSIQWQIKM 15
AtGS2 ---------- ---------- ---------- ---------- ---------- ---------- -----MAQIL AASPTCQMRV 15
OsGS2 ---------- ---------- ---------- ---------- ---------- ---------- -----MAQAV VPAMQCQVGA 15
SbGS2 MPVAVAATGC EIFIRAPPPQ PDTRLLSLSP RRLVSSRVVC ARHRSRLPSG RRGAAAGPSS VGSGMAAQAV VPAMQCQVGV 80
HvGS2 ---------- ---------- ---------- ---------- ---------- --MQVRRDDD GAGGCAGDAV PGGGEGQDGV 28
BdGS2 ---------- ---------- ---------- ---------- ---------- ---------- ---------- ------MAQA 4
Consensus ---------- ---------- ---------- ---------- ---------- ---------- ---------- ----------
100 120 140 160
SpGS1;1 ---------- ---------- ---------- ---------- ---------- ----MALLTD LINLDLSDTT EKIIAEYIWI 26
OsGS1;1 ---------- ---------- ---------- ---------- ---------- ----MASLTD LVNLNLSDTT EKIIAEYIWI 26
SbGS1;1 ---------- ---------- ---------- ---------- ---------- ----MASLTD LVNLSLSDTT EKIIAEYIWI 26
HvGS1;1 ---------- ---------- ---------- ---------- ---------- ----MALLTD LLNLDLSGST EKIIAEYIWI 26
BdGS1;1 ---------- ---------- ---------- ---------- ---------- ----MALLTD LLNLDLSDST EKIIAEYIWI 26
NnGLN1;1 ---------- ---------- ---------- ---------- ---------- ----MSLLSD LVNMNLSDST EKVIAEYIWV 26
NnGLN1;2 ---------- ---------- ---------- ---------- ---------- ----MSLLSD LINLNLSDTT EKIIAEYIWV 26
NnGLN1;3 ---------- ---------- ---------- ---------- ---------- ----MSLLND LINLNLSETT EKIIAEYIWI 26
OsGS1;2 ---------- ---------- ---------- ---------- ---------- ----MANLTD LVNLNLSDCS DKIIAEYIWV 26
SbGS1;2 ---------- ---------- ---------- ---------- ---------- ----MASLTD LVNLDLSDCT DKIIAEYIWI 26
BdGS1;2 ---------- ---------- ---------- ---------- ---------- ----MASLAD LVNLDLSDCT DKIIVEYLWV 26
HvGS1;2 ---------- ---------- ---------- ---------- ---------- ----MASLAD LVNLNLSDCT DKVIVEYLWV 26
AtGLN1;1 ---------- ---------- ---------- ---------- ---------- ----MSLVSD LINLNLSDST DKIIAEYIWV 26
AtGLN1;2 ---------- ---------- ---------- ---------- ---------- ----MSLLAD LVNLDISDNS EKIIAEYIWV 26
AtGLN1;4 ---------- ---------- ---------- ---------- ---------- ----MSSLAD LINLDLSDST DQIIAEYIWV 26
AtGLN1;3 ---------- ---------- ---------- ---------- ---------- ----MSLLSD LVNLNLTDAT GKIIAEYIWI 26
SpGS1;2 ---------- ---------- ---------- ---------- ---------- ----MALLTE LQNLDLSEST DKIIAEYIWI 26
OsGS1;3 ---------- ---------- ---------- ---------- ---------- --MSSSLLTD LVNLDLSEST DKVIAEYIWV 28
SbGS1;3 ---------- ---------- ---------- ---------- ---------- ----MALLSD LINLDLSDCT EKIIAEYIWV 26
HvGS1;3 ---------- ---------- ---------- ---------- ---------- ----MSRLAD LLSLDLSGCT GKIIAEYIWV 26
BdGS1;3 ---------- ---------- ---------- ---------- ---------- ----MSLLTD LLNLDLSDCT GKIIAEYIWV 26
SpGS1;3 ---------- ---------- ---------- ---------- ---------- ----MALLND LVNLDLVDST EKIIAEYIWI 26
AtGS1;5 ---------- ---------- ---------- ---------- ---------- ---MTSPLSD LLNLDLSD-T KKIIAEYIWI 26
SpGS2 ----T-IRPA LA---R--NP LLLAQRGVPA SRRGGSIRYR GFSVKA---- --SAVSRLED LLNLDVSPYT DKIIAEYIWI 80
NnGS2 TKNSSSARPI TA---KMWSS LLLNQKRKGA AK--GSAKFR VFALQS---- ESSTVNRLED LLKLDTTPYT DKIIAEYIWI 86
AtGS2 PKHSSVIAS- SS---KLWSS VVLKQKKQSN NK--VRG-FR VLALQS---- DNSTVNRVET LLNLDTKPYS DRIIAEYIWI 84
OsGS2 VRARPAAAA- AAAGGRVWGV R-RTGRG--T S--------- GFRVMAVSTE TTGVVTRMEQ LLNMDTTPFT DKIIAEYIWV 82
SbGS2 KAAAGARARP AAAGGRVWGV RSRTGRGGAS P--------- GFKVMAVSTG STGVVPRLEQ LLNMDTTPYT DKIIAEYIWV 151
HvGS2 PARQPA---- ----GRVWGV -SRAAR--AT S--------- GFKVLALGPE TTGVIQRMQQ LLDMDTTPFT DKIIAEYIWV 88
BdGS2 VVPAMQCQMG ALGKSAVRAR PAAAGGRVWG VRRSGARGTS GFKVLALGTE TTGVVPRLEQ LLNMDTKPYS DKIIAEYIWV 84
Consensus ---------- ---------- ---------- ---------- ---------- ----MXLLTD LLNLDLSDST DKIIAEYIWX

```
                                   180                  200                  220                  240
SpGS1;1    GGSGLDMRSK ARTIAGPVDD PSKLPKWNYD GSSTGQAPGE DSEVILYPQA IFKDPFRRGN NILVMCDCYT PAGEPIPTNK 106
OsGS1;1    GGSGMDLRSK ARTLSGPVTD PSKLPKWNYD GSSTGQAPGE DSEVILYPQA IFKDPFRKGN NILVMCDCYT PAGEPIPTNK 106
SbGS1;1    GGSGMDLRSK ARTLSGPVTD PSKLPKWNYD GSSTGQAPGE DSEVILYPQA IFKDPFRRGN NILVMCDCYT PAGEPIPTNK 106
HvGS1;1    GGSGMDLRSK ARTLPGPVTD PSKLPKWNYD GSSTGQAPGE DSEVILYPQA IFKDPFRKGN NILVMCDCYT PAGEPIPTNK 106
BdGS1;1    GGSGMDLRSK ARTLSGPVTD PSKLPKWNYD GSSTGQAPGE DSEVILYPQA IFKDPFRRGN NILVMCDCYT PAGEPIPTNK 106
NnGLN1;1   GGSGLDMRSK ARTLSGPVSD PSKLPKWNYD GSSTGQAPGE DSEVILYPQA IFRDPFRRGN NILVMCDTYT PSGEPIPTNK 106
NnGLN1;2   GGSGMDMRSK AKTLSGPVSD PSKLPKWNYD GSSTGQAPGE DSEVILYPQA IFRDPFRRGN NILVMCDTYT PAGEPIPTNK 106
NnGLN1;3   GGSGMDLRSK ARTLSGPVTD PQKLPKWNYD GSSTGQAPGE DSEVILYPQA IFRDPFRRGN NILVMCDAYT PAGEPIPTNK 106
OsGS1;2    GGSGIDLRSK ARTVKGPITD VSQLPKWNYD GSSTGQAPGE DSEVILYPQA IFKDPFRRGD NILVMCDCYT PQGEPIPTNK 106
SbGS1;2    GGSGIDLRSK ARTVKGPITD PSQLPKWNYD GSSTGQAPGE DSEVILYPQA IFKDPFRKGD NILVMCDCYT PQGEPIPTNK 106
BdGS1;2    GGSGIDIRSK ARTVNGPITD ASQLPKWNYD GSSTGQAPGE DSEVILYPQA IFKDPFRRGD NILVMCDCYT PQGVPIPTNK 106
HvGS1;2    GGSGIDIRSK ARTVNGPITD ASQLPKWNYD GSSTGQAPGE DSEVILYPQA IFKDPFRRGD NILVMCDCYT PQGVPIPTNK 106
AtGLN1;1   GGSGMDMRSK ARTLPGPVTD PSQLPKWNYD GSSTGQAPGE DSEVILYPQA IFKDPFRRGN NILVMCDAYT PAGEPIPTNK 106
AtGLN1;2   GGSGMDMRSK ARTLPGPVTD PSKLPKWNYD GSSTGQAPGQ DSEVILYPQA IFKDPFRRGN NILVMCDAYT PAGEPIPTNK 106
AtGLN1;4   GGSGLDMRSK ARTLPGPVTD PSQLPKWNYD GSSTGQAPGD DSEVIIYPQA IFKDPFRRGN NILVMCDAYT PAGEPIPTNK 106
AtGLN1;3   GGSGMDIRSK ARTLPGPVTD PSKLPKWNYD GSSTGQAAGE DSEVILYPQA IFKDPFRKGN NILVMCDAYT PAGDPIPTNK 106
SpGS1;2    GGSGLDMRSK ARTLSGPVDD PSKLPKWNYD GSSTGQAPGE DSEVILYPQA IFKDPFRRGN NILVMCDSYT PAGEPIPTNK 106
OsGS1;3    GGTGMDVRSK ARTLSGPVDD PSKLPKWNFD GSSTGQATGD DSEVILHPQA IFRDPFRKGK NILVMCDCYA PNGEPIPTNN 108
SbGS1;3    GGSGMDVRSK ARTLSGPVDD PSKLPKWNFD GSSTGQAPGD DSEVILCPRA IFRDPFRKGK NILVMCDCYE PNGKPIPSNK 106
HvGS1;3    GGTGMDVRSK ARTLPGPVDD PSKLPKWNFD GSSTGQATGD DSEVILRPQA IFRDPFRKGN NILVICDCYA PTGEPIPSNK 106
BdGS1;3    GGTGMDMRSK ARTLPGPVDD PSKLPKWNFD GSSTGQATGD DSEVILCPQA IFRDPFRKGK NILVMCDCYA PTGEPIPSNK 106
SpGS1;3    GGSGMDMRSK ARTLPGSVSD PKELPKWNYD GSSTGQAPGE NSEVILHPQA IFKDPFRRGN NILVMCDAHT PAGEPIPTNK 106
AtGS1;5    GGSGMDIRSK ARTLPGPVSN PTKLPKWNYD GSSTDQAAGD DSEVILYPQA IFKDPFRKGN NILVMCDAYR PAGDPIPTNN 106
SpGS2      GGSGIDVRSK SRTISKAVEH PSELPKWNYD GSSTGQAPGE DSEVILYPQA IFKDPFRGGN NILVMCDSYT PNGEPIPTNK 160
NnGS2      GGSGIDLRSK SRTISKPVEH PSELPKWNYD GSSTGQAPGE DSEVILYPQA IFKDPFRGGN NILVICDSYT PAGEPIPTNK 166
AtGS2      GGSGIDLRSK SRTIEKPVED PSELPKWNYD GSSTGQAPGE DSEVILYPQA IFRDPFRGGN NILVICDTWT PAGEPIPTNK 164
OsGS2      GGTGIDLRSK SRTISKPVED PSELPKWNYD GSSTGQAPGE DSEVILYPQA IFKDPFRGGN NILVMCDTYT PAGEPIPTNK 162
SbGS2      GGSGIDIRSK SRTISKPVED PSELPKWNYD GSSTGQAPGE DSEVILYPQA IFKDPFRGGN NILVICDTYT PQGEPLPTNK 231
HvGS2      GGSGIDLRSK SRTISKPVED PSELPKWNYD GSSTGQAPGE DSEVILYPQA IFKDPFRGGN NILVICDTYT PQGEPIPTNK 168
BdGS2      GGSGIDIRSK SRTISKPVAD PSELPKWNYD GSSTGQAPGE DSEVILYPQA IFKDPFRGGN NILVVCDTYT PAGEPIPTNK 164
Consensus  GGSGMDLRSK ARTLSGPVTD PSKLPKWNYD GSSTGQAPGE DSEVILYPQA IFKDPFRRGN NILVMCDCYT PAGEPIPTNK

                                   260                  280                  300                  320
SpGS1;1    RFNAAKVFSN PDVIAEVPWY GIEQEYTLLQ KDIGWPLGWP VGGFPGPQGP YYCGTGADKA FGRDIVDAHY KACLYAGINI 186
OsGS1;1    RHNAAKIFSS PEVASEEPWY GIEQEYTLLQ KDINWPLGWP VGGFPGPQGP YYCGIGADKS FGRDIVDSHY KACLYAGINI 186
SbGS1;1    RHNAAKIFSN PEVAAEEPWY GIEQEYTLLQ KDTNWPLGWP LGGFPGPQGP YYCGIGADKS FGRDIVDAHY KACIYAGINI 186
HvGS1;1    RYNAAKIFSN PDVAKEEPWY GIEQEYTLLQ KDINWPLGWP VGGFPGPQGP YYCGIGADKS FGRDIVDSHY KACLFAGVNI 186
BdGS1;1    RHAAAKIFSN PAVSAEEPWY GIEQEYTLLQ KDINWPLGWP VGGFPGPQGP YYCSIGADKS FGRDIVDSHY KACLYAGINI 186
NnGLN1;1   RCNAAKIFNH PDVLAEELWF GIEQEYTLLQ KDVKWPVGWP VGGFPGPQGP YYCGVGADKA WGRDIVDSHY KACLYAGINI 186
NnGLN1;2   RCNAAKIFSH PDVLAEEPWY GIEQEYTLLQ KDVKWPLGWP VGGFPGPQGP YYCGVGADKA WGRDIVDSHY KACLYAGVNI 186
NnGLN1;3   RYNAAKIFSH PDVSAEEPWY GIEQEYTLLQ KDVHWPIGWP VGGFPGPQGP YYCGAGADKA FGRDIVDSHY KACLYAGINI 186
OsGS1;2    RHSAAKIFSH PDVVAEVPWY GIEQEYTLLQ KDVNWPLGWP VGGFPGPQGP YYCAAGAEKA FGRDIVDAHY KACIYAGINI 186
SbGS1;2    RYNAAKVFSH PDVAAEVPWY GIEQEYTLLQ KDVNWPLGWP VGGYPGPQGP YYCAAGADKA FGRDVVDAHY KACLYAGINI 186
BdGS1;2    RHNAAKIFNN PKVAAEVTWY GIEQEYTLLQ KDVNWPLGWP VGGYPGPQGP YYCAAGADKA FGRDIVDAHY KACLYAGINI 186
HvGS1;2    RHNAAKIFNS AKVAAEETWY GIEQEYTLLQ KDVNWPLGWP IGGYPGPQGP YYCAAGADKA FGRDIVDAHY KACLYAGINI 186
AtGLN1;1   RHAAAKVFSN PDVAAEVPWY GIEQEYTLLQ KDVKWPVGWP IGGYPGPQGP YYCGIGADKS FGRDVVDSHY KACLYAGINI 186
AtGLN1;2   RHAAAEIFAN PDVIAEVPWY GIEQEYTLLQ KDVNWPLGWP IGGFPGPQGP YYCSIGADKS FGRDIVDAHY KASLYAGINI 186
AtGLN1;4   RHAAAKIFED PSVVAEETWY GIEQEYTLLQ KDIKWPVGWP VGGFPGPQGP YYCGVGADKA FGRDIVDSHY KACLYAGINV 186
AtGLN1;3   RHNAAKIFSH PDVAKEEPWY GIEQEYTLMQ KDVNWPIGWP VGGYPGPQGP YYCGVGADKA IGRDIVDAHY KACLYAGIGI 186
SpGS1;2    RYKAAQIFSD PAVVAEVPWY GLEQEYTLLQ KDVKWPLGWP LGGFPAPQGP YYCGTGVDKA FGRDIVDAHY KACLYAGINI 186
OsGS1;3    RYNAARIFSH PDVKAEEPWY GIEQEYTLLQ KHINWPLGWP LGGYPGPQGP YYCAAGADKS YGRDIVDAHY KACLFAGINI 188
SbGS1;3    RHGAATIFSH PDVKAEEPWF GIEQEYTLLQ KDTNWPLGWP LGGYPGPQGP YYCAAGADKS YGREIVDAHY KACLYAGIDI 186
HvGS1;3    RYNAARIFGH PDVKSEEPWY GIEQEYTLLQ KDTNWPIGWP LGGYPGPQGP YYCAAGAEKS YGRDIVDAHY KACLYAGINI 186
BdGS1;3    RSSAANIFSH PDVKAEEPWY GIEQEYTLLQ KDINWPLGWP LGGYPGAQGP YYCATGAEKS YGRDIVDAHY KACLYAGVNI 186
SpGS1;3    RFLAEKIFSH PDVIAEEPWF GIEQEYTLLQ RDVHWPLGWP VGGFPRPQGP YYCGVGADKA FGRDIVDSHY KACLYAGINV 186
AtGS1;5    RHKAVKIFDH PNVKAEEPWF GIEQEYTLLK KDVKWPLGWP LGGFPGPQGP YYCAVGADKA FGRDIVDAHY KACLYSGLSI 186
SpGS2      RYRAAQIFSD PKVVAEVPWF GIEQEYTLLQ SNVKWPLGWP IGGYPGPQGP YYCSAGADKS FGRDISDAHY KACLYAGINI 240
NnGS2      RYRAAQIFSD PKVVQEVPWY GIEQEYTLLQ TNVKWPLGWP IGAYPGPQGP YYCGVGADKS FGRDISDAHY KACLYSGINI 246
AtGS2      RAKAAEIFSN KKVSGEVPWF GIEQEYTLLQ QNVKWPLGWP VGAFPGPQGP YYCGVGADKI WGRDISDAHY KACLYAGINI 244
OsGS2      RNRAAQVFSD PKVVSQVPWF GIEQEYTLLQ RDVNWPLGWP VGGYPGPQGP YYCAVGSDKS FGRDISDAHY KACLYAGINI 242
SbGS2      RHRAAQIFSD PKVVEQVPWF GIEQEYTLLQ KDVNWPLGWP VGGYPGPQGP YYCAVGADKS FGRDISDAHY KACLYAGINI 311
HvGS2      RHMAAQIFSD PKVTSQVPWF GIEQEYTLMQ RDVNWPLGWP VGGYPGPQGP YYCAVGSDKS FGRDISDAHY KACLYAGIEI 248
BdGS2      RHRAAQIFSD PKVASQVPWY GIEQEYTLLQ RDVNWPLGWP VGGYPGPQGP YYCAVGSDKS FGRDISDAHY KACIYAGIEI 244
Consensus  RHNAAKIFSH PDVAAEEPWY GIEQEYTLLQ KDVNWPLGWP VGGXPGPQGP YYCGVGADKS FGRDIVDAHY KACLYAGINI
```

		340		360		380		400	
SpGS1;1	SGINGEVMPG	QWEFQVGPVV	GISAGDQVWV	ARYILERITE	IAGVVVSFDP	KPIKGDWNGA	GAHTNYSTQS	MRNDGGYEVI	266
OsGS1;1	SGINGEVMPG	QWEFQVGPSV	GISAGDQVWV	ARYILERITE	IAGVVVSFDP	KPIPGDWNGA	GAHTNYSTKS	MRNDGGYEII	266
SbGS1;1	SGINGEVMPG	QWEFQVGPSV	GISSGDQVWV	ARYILERITE	IAGVVLTFDP	KPIPGDWNGA	GAHTNYSTKS	MRNEGGYEVI	266
HvGS1;1	SGINGEVMPG	QWEFQVGPTV	GISAGDQVWV	ARYILERITE	IAGVVVTFDP	KPIPGDWNGA	GAHTNYSTES	MRNDGGFKVI	266
BdGS1;1	SGINGEVMPG	QWEFQVGPSV	GISAGDQVWV	ARYLLERITE	IAGVVVSFDP	KPIPGDWNGA	GAHTNYSTES	MRKDGGFQVI	266
NnGLN1;1	SGINGEVMPG	QWEFQVGPSV	GISAGDELWI	ARYILERITE	IAGVVLSFDP	KPIQGDWNGA	GAHTNYSTKS	MRSEGGYEVI	266
NnGLN1;2	SGINGEVMPG	QWEFQVGPSI	GISAGDELWI	ARYILERITE	IAGVVLSFDP	KPIQGDWNGA	GAHTNFSTKS	TRNEGGYEVI	266
NnGLN1;3	SGINGEVMPG	QWEFQVGPAV	GISAGDELWV	ARYILERITE	IAGVVLSFDP	KPIKGDWNGA	GAHTNYSTKS	MRNDGGIDVI	266
OsGS1;2	SGINGEVMPG	QWEFQVGPSV	GIAAADQVWV	ARYILERVTE	VAGVVLSLDP	KPIPGDWNGA	GAHTNFSTKS	MREPGGYEVI	266
SbGS1;2	SGINGEVMPG	QWEFQVGPSV	GISAGDEIWV	ARYILERITE	IAGIVLSLDP	KPIQGDWNGA	GAHTNYSTKS	MREAGGYEVI	266
BdGS1;2	SGINGEVMPG	QWEFQVGPSV	GIAASDQLWV	ARYILERITE	VAGVVLSLDP	KPIPGDWNGA	GAHTNYSTKS	MREAGGFEVI	266
HvGS1;2	SGINGEVMPG	QWEFQVGPSV	GIAASDQLWV	ARYILERITE	VAGVVLSLDP	KPIPGDWNGA	GAHTNYSTKS	MRQAGGYEVI	266
AtGLN1;1	SGINGEVMPG	QWEFQVGPAV	GISAADEIWV	ARYILERITE	IAGVVVSFDP	KPIPGDWNGA	GAHCNYSTKS	MREEGGYEII	266
AtGLN1;2	SGINGEVMPG	QWEFQVGPSV	GISAADEIWI	ARYILERITE	IAGVVVSFDP	KPIPGDWNGA	GAHTNYSTKS	MREEGGYEII	266
AtGLN1;4	SGTNGEVMPG	QWEFQVGPTV	GIAAADQVWV	ARYILERITE	LAGVVLSLDP	KPIPGDWNGA	GAHTNYSTKS	MREDGGYEVI	266
AtGLN1;3	SGINGEVMPG	QWEFQVGPVE	GISSGDQVWV	ARYLLERITE	ISGVIVSFDP	KPVPGDWNGA	GAHCNYSTKT	MRNDGGLEVI	266
SpGS1;2	SGINGEVMPG	QWEFQVGPAV	GIAAADELWV	ARYILERITE	VAGVVLSLHP	KPIKGDWNGA	GCHTNYSTKT	MREEGGFEVI	266
OsGS1;3	SGINAEVMPG	QWEFQIGPVV	GVSAGDHVWV	ARYILERITE	IAGVVVSFDP	KPIPGDWNGA	GAHTNYSTKS	MRSNGGYEVI	268
SbGS1;3	SGINGEVMPG	QWEFQVGPAV	GVSAGDQLWV	ARYILERITE	IAGVVVSFDP	KPIPGDWNGA	GAHTNYSTKS	MRSDGGYEVI	266
HvGS1;3	GGINAEVMPG	QWEFQVGPSV	GISAGDELWA	ARYILERITE	IAGVVVSFDP	KPIPGEWNGA	GAHTNYSTKS	MRSEGGYEVI	266
BdGS1;3	GGINAEVMPG	QWEFQVGPSV	GISAGDELWV	ARYILERITE	IAGVVVSFDP	KPVPGEWNGA	GAHTNYSTKS	MRSEGGYEVI	266
SpGS1;3	SGVNGEVMPG	QWEFQVGPAV	GISAGDQIWA	ARYILERITE	IAGVLLSFDP	KPIKGDWNGA	GAHTNYSTNS	MRSNGGMDEI	266
AtGS1;5	GGANGEVMPG	QWEFQISPTV	GIGAGDQLWV	ARYILERITE	ICGVIVSFDP	KPIQGDWNGA	AAHTNFSTKS	MRKDGGLDLI	266
SpGS2	SGTNGEVMPG	QWEYQVGPSV	GIDAGDHIWV	SRYILERITE	QAGVVLSLDP	KPIEGDWNGA	GCHTNYSTKT	MREDGGFEAI	320
NnGS2	SGTNGEVMPG	QWEYQVGPSV	GIEAGDHIWC	SRYILERITE	QAGVVLTLDP	KPIEGDWNGA	GCHTNYSTKS	MREEGGFEVI	326
AtGS2	SGTNGEVMPG	QWEFQVGPSV	GIDAGDHVWC	ARYLLERITE	QAGVVLTLDP	KPIEGDWNGA	GCHTNYSTKS	MREEGGFEVI	324
OsGS2	SGTNGEVMPG	QWEYQVGPSV	GIEAGDHIWI	SRYILERITE	QAGVVLTLDP	KPIQGDWNGA	GCHTNYSTKS	MREDGGFEVI	322
SbGS2	SGTNGEVMPG	QWEYQVGPSV	GIEAGDHIWI	SRYILERITE	QAGVVLTLDP	KPIQGDWNGA	GCHTNYSTKT	MREDGGFEDI	391
HvGS2	SGTNGEVMPG	QWEYQVGPSV	GIDAGDHIWA	SRYILERITE	QAGVVLTLDP	KPIQGDWNGA	GCHTNYSTLS	MREDGGFDVI	328
BdGS2	SGTNGEVMPG	QWEYQVGPSV	GIDAGDHIWA	SRYILERITE	QAGVVLTLDP	KPIQGDWNGA	GCHTNYSTKA	MREDGGFEVI	324
Consensus	SGINGEVMPG	QWEFQVGPSV	GISAGDQXWV	ARYILERITE	IAGVVLSFDP	KPIPGDWNGA	GAHTNYSTKS	MREDGGYEVI	

		420		440		460		480	
SpGS1;1	KKAIEKLGLR	HKEHIAAYGE	GNERRLTGRH	ETADINTFLW	GVANRGASIR	VGRETEQNGK	GYFEDRRPAS	NMDPYVVTAM	346
OsGS1;1	KSAIEKLKLR	HKEHISAYGE	GNERRLTGRH	ETADINTFSW	GVANRGASVR	VGRETEQNGK	GYFEDRRPAS	NMDPYIVTSM	346
SbGS1;1	KAAIEKLKLR	HKEHIAAYGE	GNERRLTGRH	ETADINTFSW	GVANRGASVR	VGRETEQNGK	GYFEDRRPAS	NMDPYVVTSM	346
HvGS1;1	VDAVEKLKLK	HKEHIAAYGE	GNERRLTGKH	ETADINTFSW	GVANRGASVR	VGRETEQNGK	GYFEDRRPAS	NMDPYVVTSM	346
BdGS1;1	VAAVEKLKLR	HKEHIAAYGE	GNERRLTGKH	ETADIHTFSW	GVANRGASVR	VGRETEQNGK	GYFEDRRPAS	NMDPYVVTAM	346
NnGLN1;1	KKAIEKLGLR	HKEHISAYGE	GNERRLTGRH	ETADINTFVW	GVANRGASIR	VGRDTERAGK	GYFEDRRPAS	NMDPYVVTSM	346
NnGLN1;2	KKAIKKLGLR	HKEHIAAYGE	GNERRLTGRH	ETADINTFAW	GVANRGASIR	VGRDTERAGK	GYFEDRRPAS	NMDPYVVTSM	346
NnGLN1;3	KKAIEKLGLR	HKEHIAGYGE	GNERRLTGRH	ETADINTFSW	GVANRGASIR	VGRDTEKAGK	GYFEDRRPAS	NMDPYVVTSM	346
OsGS1;2	KKAIDKLALR	HKEHIAAYGE	GNERRLTGRH	ETADINTFKW	GVANRGASIR	VGRDTEKEGK	GYFEDRRPAS	NMDPYVVTGM	346
SbGS1;2	KKAIEKLGKR	HTEHIAAYGE	GNERRLTGRH	ETADINTFKW	GVANRGASIR	VGRDTEREGK	GYFEDRRPAS	NMDPYVVTGM	346
BdGS1;2	KKAIEKLGKR	HTEHIAAYGE	GNERRLTGHH	ETADINTFKW	GVANRGASIR	VGRDTEKDGK	GYFEDRRPAS	NMDPYVVTSM	346
HvGS1;2	KKAIEKLGKR	HMQHIAAYGE	GNERRLTGHH	ETADINTFKW	GVADRGASIR	VGRDTEKDGK	GYFEDRRPAS	NMDPYVVTSM	346
AtGLN1;1	KKAIDKLGLR	HKEHIAAYGE	GNERRLTGHH	ETADINTFLW	GVANRGASIR	VGRDTEKEGK	GYFEDRRPAS	NMDPYIVTSM	346
AtGLN1;2	KKAIEKLGLR	HKEHISAYGE	GNERRLTGHH	ETADINTFLW	GVANRGASIR	VGRDTEKEGK	GYFEDRRPAS	NMDPYVVTSM	346
AtGLN1;4	KKAIEKLGLR	HKEHIAAYGE	GNERRLTGKH	ETADINTFLW	GVANRGASIR	VGRDTEQAGK	GYFEDRRPAS	NMDPYTVTSM	346
AtGLN1;3	KKAIGKLQLK	HKEHIAAYGE	GNERRLTGKH	ETADINTFSW	GVANRGASVR	VGRDTEKEGK	GYFEDRRPAS	NMDPYVVTSM	346
SpGS1;2	KKAIDKLGLR	HKEHIQAYGE	DNEERLTGRH	ETADINTFKW	GVANRGASIR	VGRDTEKEGK	GYFEDRRPAS	NMDPYVVTSM	346
OsGS1;3	KKAIKKLGMR	HREHIAAYGD	GNERRLTGRH	ETADINNFVW	GVANRGASVR	VGRDTEKDGK	GYFEDRRPAS	NMDPYLVTAM	348
SbGS1;3	KKAISKLGLR	HREHIAAYGD	GNERRLTGRH	ETADINTFIW	GVANRGASVR	VGRDTEKEGK	GYFEDRRPAS	NMDPYVVTSL	346
HvGS1;3	KRAIKKLEAR	HTEHIAAYGE	GNERRLTGRH	ETADINTFVW	GVANRGASVR	VGRDTEKEGR	GYFEDRRPAS	NMDPYVVTSM	346
BdGS1;3	KKAIEKLEAR	HGEHIAAYGE	GNERRLTGRN	ETADINTFVW	GVANRGASVR	VGRDTEKEGK	GYFEDRRPGS	NMDPYVVTSK	346
SpGS1;3	RRAIEKLGLR	HMEHITAYGD	GNERRLTGRH	ETADIRTFSW	GIADRGASVR	VGRETEMAGN	GYFEDRRPAS	NMDPYVVTSM	346
AtGS1;5	KEAIKKLEVK	HKQHIAAYGE	GNERRLTGKH	ETADINTFSW	GVADRGASVR	VGRDTEKEGK	GYFEDRRPSS	NMDPYLVTSM	346
SpGS2	KKAVGNLALR	HKEHISAYGE	GNERRLTGKH	ETANINTFSW	GVANRGCSVR	VGRETERQGK	GYLEDRRPAS	NMDPYVVTSL	400
NnGS2	KKAILNLSLR	HKEHISAYGE	GNERRLTGKH	ETANINTFSW	GVANRGCSIR	VGRDTEKQGK	GYLEDRRPAS	NMDPYVVTSL	406
AtGS2	KKAILNLSLR	HKEHISAYGE	GNERRLTGKH	ETASIDQFSW	GVANRGCSIR	VGRDTEAKGK	GYLEDRRPAS	NMDPYIVTSL	404
OsGS2	KKAILNLSLR	HDLHISAYGE	GNERRLTGLH	ETASIDNFSW	GVANRGCSIR	VGRDTEAKGK	GYLEDRRPAS	NMDPYVVTAL	402
SbGS2	KRAILNLSLR	HDLHISAYGE	GNERRLTGKH	ETASIETFSW	GVANRGCSVR	VGRDTEAKGK	GYLEDRRPAS	NMDPYIVTGL	471
HvGS2	KKAILNLSLR	HDLHIAAYGE	GNERRLTGLH	ETASISDFSW	GVANRGCSIR	VGRDTEAKGK	GYLEDRRPAS	NMDPYTVTAL	408
BdGS2	KKAILNLSLR	HDLHISAYGE	GNERRLTGLH	ETASISDFSW	GVANRGCSIR	VGRDTEANGK	GYLEDRRPAS	NMDPYTVTAL	404
Consensus	KKAIEKLGLR	HKEHIAAYGE	GNERRLTGRH	ETADINTFSW	GVANRGASIR	VGRDTEKEGK	GYFEDRRPAS	NMDPYVVTSM	

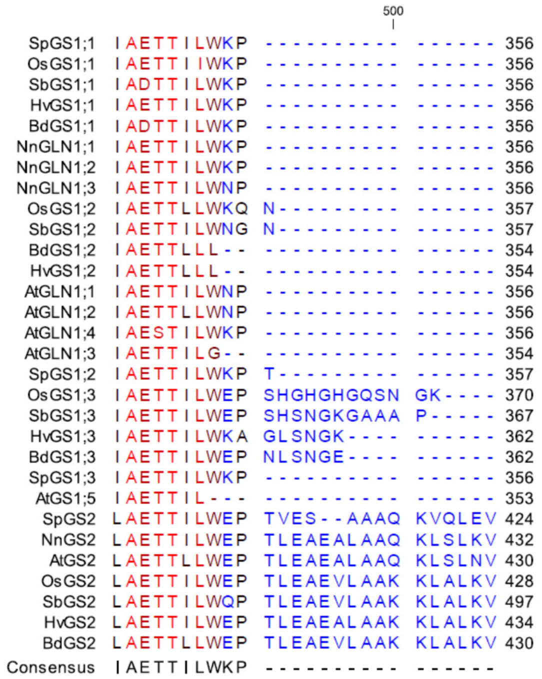

B

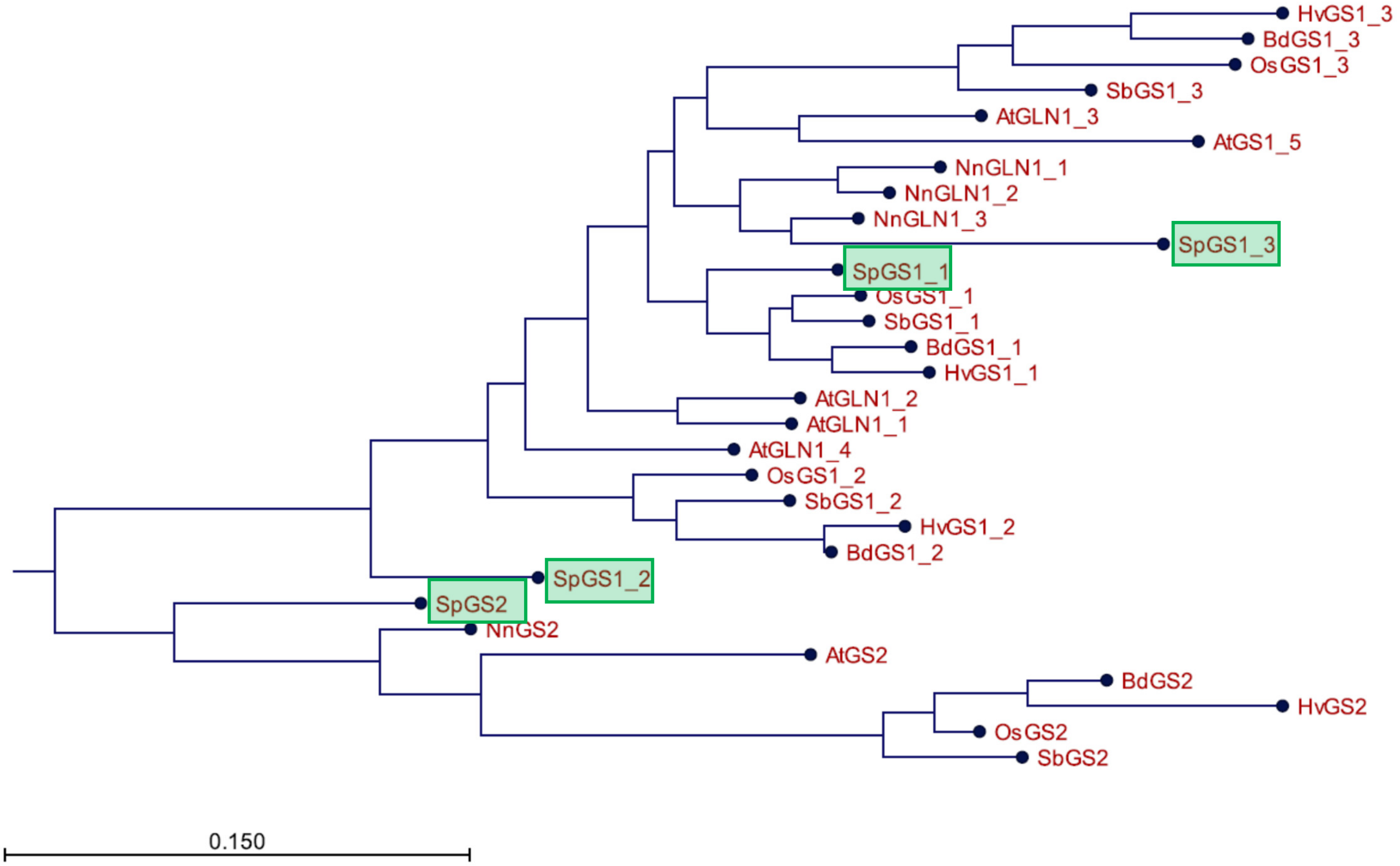

Figure S9. Sequence alignment of GS proteins from *S.polyrhiza* with other representative species **(A)** and the resulted phylogenetic tree **(B).** AtGln1;1 – *A. thaliana,* NP_198576.1, AtGln1;2 - *A. thaliana,* NP_176794.1, AtGln1;3 - *A. thaliana,* NP_188409.1; AtGln1;4 - *A. thaliana,* NP_001331815.1; AtGln1;5 - *A. thaliana,* NP_175280.1; AtGS2 - *A. thaliana,* NP_001031969.1; BdGS1;1 - *B. distachyon,* XP_010236151.1, BdGS1;2 - *B. distachyon*, XP_003558466.1, BdGS1;3 - *B. distachyon*, XP_003560727.2, BdGS2 - *B. distachyon*, XP_003580719.1, HvGS1;1 - *H. vulgare,* AFX60875.1; HvGS1;2 - *H. vulgare,* AFX60876.1, HvGS1;3 - *H. vulgare,* AFX60877.1, HvGS2 - *H. vulgare,* P13564.2, NnGS1;1 - *N. nucifera,* XP_010271383.1, NnGS1;2 - *N. nucifera,* XP_010271347.1; NnGS1;3 - *N. nucifera,* XP_010250142.1, NnGS2 - *N. nucifera,* XP_010255852.1, OsGS1;1 - *O. sativa,* XP_015626102.1, OsGS1;2 - *O. sativa,* XP_015631679.1, OsGS1;3 - *O. sativa,* XP_015628694.1, OsGS2 - *O. sativa,* XP_015635322.1.SbGS1;1 - *S. bicolor,* XP_021313946.1, SbGS1;2 - *S. bicolor,* XP_002465624.1, SbGS1;3 - *S. bicolor,* XP_021306978.1,

SbGS2 - *S. bicolor*, XP_021319069.1, SpGS1;1 - *S. polyrhiza* (sequence translated from Acc. ID MZ605906) , SpGS1;2 - *S. polyrhiza* (sequence translated from Acc. ID MZ605907); SpGS1;3 - *S. polyrhiza* (sequence translated from Acc. ID MZ605908), SpGS2 - *S. polyrhiza* (sequence translated from Acc. MZ605909)

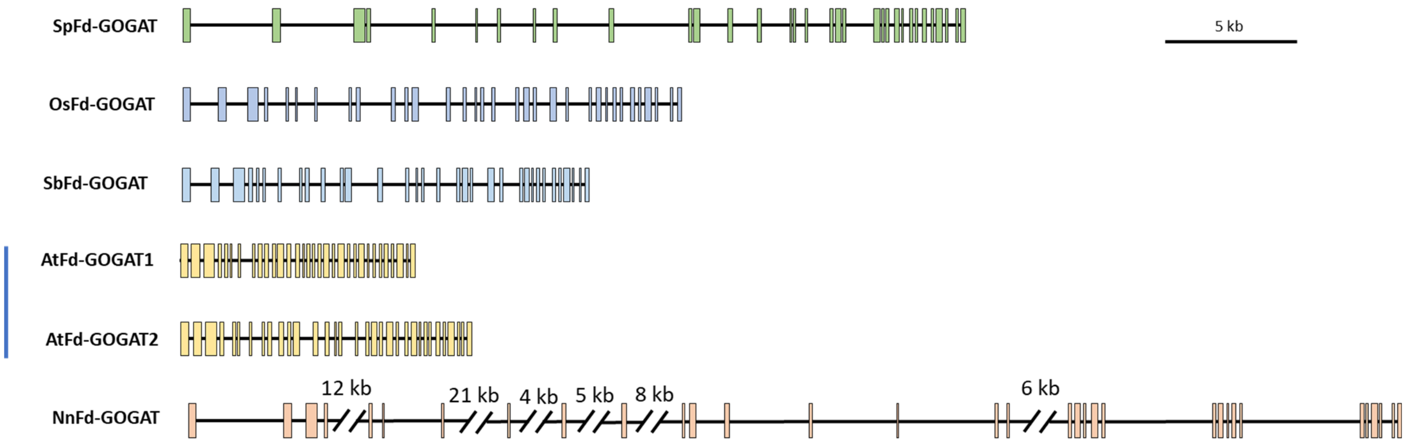

Figure S10. Diagrammatic representation of the structure of *Fd-GOGAT* genes.
At Fd-GOGAT1 - A. thaliana, At5g04140, *AtFd-GOFAT2 - A. thaliana*, At2g41220, *NnFd-GOGAT - N. nucifera*, LOC104611350, *OsFd-GOGAT - O. sativa*, LOC4344164, *SbFd-GOGAT - S. bicolor*, LOC8073135, *SpFd-GOGAT – S. polyrhiza* CP019094, the 1st chromosome, position from 4454784 to 4484474. Coding sequences are colored boxes. Introns are black lines. The bar is 5 kb.

A

```
                      20          40          60          80
SpFd-GOGAT  MAQSAAA--A AAVSAAPRAL LPSPTANSDS ---------A FFSRNRSLLL RQ------RS IVVHRRL-CG A---GRG-SR 58
OsFd-GOGAT  MATLPRAAAA AAPSPAA-AL LPLPRAAP-- LLAGR---AA ARSAARRLRA R---GTRAPP LAAARRGW-- ----GGV-S- 63
BdFd-GOGAT  ------MAAT LPRAVAPPSL LPLPRAAP-- ------LLLA GRAAARRLRA R---GARAPA LAAARRSWAV S--------- 54
SbFd-GOGAT  MATLPRAAP- --PTPAA--L LPLPRAAPPL LLAGR---AA AARRSR-LRA R---G----P SAAARRSWVV ASSSSSS-S- 62
AtFd-GOGAT1 -------MAM QSLSPVPKLL STTPSS---- ---------- VLSSDKNFFF VDFVGLYCKS KRTRRRLRGD SSSSSRS-SS 58
AtFd-GOGAT2 ---------- --------MA LQSPGATGAS SSVSRLLSSA KLSSTKTIFS VDFVRSYCIS KGTKRR---N ELSGFRGYSP 59
NnFdGOGAT   -------MAL QSIVPMPQLL YSNGFSSSTS LSATK---SS IFDANRGLLF ADFIGLCCKS KRTRQRIGIG AVRRGRG-S- 68
Consensus   ------AAAA XXPSPAP-XL LPLPRAAP-S L-A-R---XA AXSAXRRLRA RD--GLXCXS KAXARRXW-X A-S-GRG-S-

                     100         120         140         160
SpFd-GOGAT  LLPSMPRSVR VGAHQREGTC SVGNSKQTVS S--------- -----RARRS K--------- -VADLNDILS ERGACGVGFI 114
OsFd-GOGAT  -----PRAVL DLPRRREAA- --EKPAQKA- ---------- ---------- ---------- --ADLNEILS ERGACGVGFV 102
BdFd-GOGAT  -----TRAVL DVPRHRAPPP A-QKPVQEA- ---------- ---------- ---------- --ADLNDILA ERGACGVGFV 95
SbFd-GOGAT  -----SRAVL GGVARREAPP APQKPTQQA- ---------- ---------- ---------- --ADLNHILS ERGACGVGFV 104
AtFd-GOGAT1 SLSRLS-SVR AVIDLERVHG VSEKDLSSPS ALRPQVRFFT DINFTNTQRA KFHPLWGSFK QVANLEDILS ERGACGVGFI 137
AtFd-GOGAT2 LLKSSLRSPF SVKAILNSDR AAGDASSSFS DLKP------ ---------- ---------- QVAYLEDIIS ERGACGVGFI 113
NnFdGOGAT   -LGRSWSSVK AVLDVNRVDF A-----SKES D--------- -----TVRRA E--------N EVANLNDIIS ERGACGVGFI 120
Consensus   -L---XRSVL XVPXRREXXP A-XKPXQXAS ---------- ---------- ---------- -VADLNDILS ERGACGVGFI

                     180         200         220         240
SpFd-GOGAT  ANLKNEASHK IVEDALVALG CMEHRGGCGA DNDSGDGAGL MTSVPWDLYN NWADKQGLPF LDRYKTGVGM VFLPQSDEAM 194
OsFd-GOGAT  ANLKNEPSFN IVRDALVALG CMEHRGGCGA DNDSGDGSGL MSGIPWDLFN DWANKQGLAP LDRTNTGVGM VFLPQDENSM 182
BdFd-GOGAT  ANLKNEPSFN IVRDALTALG CMEHRGGCGA DNDSGDGAGL MSGIPWDLFN DWASKQGLPP FERTNTGVGM VFLPQNEESM 175
SbFd-GOGAT  ANLKNVPSFN IVRDALMALG CMEHRGGCGA DNDSGDGAGL MSAIPWDLFD DWANKQGLAP FDRRNTGVGM VFLPQDEKSM 184
AtFd-GOGAT1 ANLDNIPSHG VVKDALIALG CMEHRGGCGA DNDSGDGSGL MSSIPWDFFN VWAKEQSLAP FDKLHTGVGM IFLPQDDTFM 217
AtFd-GOGAT2 ANLENKATHK IVNDALIALG CMEHRGGCGS DNTSGDGSGL MTSIPWDLFN EWAEKQGIAS FDRTHTGVGM LFLPRDDNIR 193
NnFdGOGAT   ANLENNASHE IIKDALTALG CMEHRGGCGA DNDSGDGSGL MTSIPWELFN NWANKQGIAS LDKLHTGVGM VFLPKDDDSM 200
Consensus   ANLKNEPSHN IVRDALXALG CMEHRGGCGA DNDSGDGSGL MSSIPWDLFN DWANKQGLAP FDRTXTGVGM VFLPQDDXSM

                     260         280         300         320
SpFd-GOGAT  EEAKKVIVNT CLKEGLEIVG WRPVPVNESV VGYYAKETLP NIQQLFVKVV KEDNVDDLER ELYICRKLIE RAVQSEKWSN 274
OsFd-GOGAT  EEAKAVVAKV FTDEGLEVLG WRTVPFNVSV VGRYAKETMP NIQQIFVKVA KEDNADDIER ELYICRKLIE RATKSASWAD 262
BdFd-GOGAT  EEAKAAVAKV FTDEGLEVLG WRPVPFNLSV VGRFAKETMP NIQQIFVKVA KEDDADDIER ELYICRKLIE RAAKSASWAD 255
SbFd-GOGAT  EEAKAATEKV FVDEGLEVIG WRPVPFNVSV VGRNAKETMP NIQQIFVKVA KEDNADDIER ELYISRKLIE RATKSFSWAD 264
AtFd-GOGAT1 QEAKQVIENI FEKEGLQVLG WREVPVNVPI VGKNARETMP NIQQVFVKIA KEDSTDDIER ELYICRKLIE RAVATESWGT 297
AtFd-GOGAT2 KEAKKVITSI FEKEGLEVLG WRDVPVEASI VGHNAKQTMP NTEQVFVRIV KDDKVDDVER ELYICRKLIE RAVASESWAS 273
NnFdGOGAT   KEAKSVIENT FKQEGLDVLG WRPVPINVAV VGYYAKETMP NIQQVFVKIS NEENIDDIER ELYICRKLIE RVSKLEKWGD 280
Consensus   EEAKAVIEXV FXXEGLEVLG WRPVPXNVSV VGRXAKETMP NIQQXFVKVA KEDNADDIER ELYICRKLIE RAVKSESWAD

                     340         360         380         400
SpFd-GOGAT  DLYFCSFSNQ TVVYKGMLRS EVLGQFYLDL QSDLYKSSFA IYHRRYSTNT SPRWPLAQPM RLLGHNGEIN TIQGNLNWMQ 354
OsFd-GOGAT  ELYFCSLSSR TIVYKGMLRS EILGQFYLDL QNELYKSPFA IYHRRYSTNT SPRWPLAQPM RLLGHNGEIN TIQGNLNWMR 342
BdFd-GOGAT  ELYFCSLSSR TIIYKGMLRS EVLGQFYLDL QNELYKSPFA IYHRRFSTNT SPRWPLAQPM RLLGHNGEIN TIQGNLNWMR 335
SbFd-GOGAT  ELYFCSLSNR TIVYKGMLRS EVLGQFYLDL QNELYKSPFA IYHRRYSTNT SPRWPLAQPM RLLGHNGEIN TIQGNLNWMR 344
AtFd-GOGAT1 ELYFCSLSNQ TIVYKGMLRS EALGLFYLDL QNELYESPFA IYHRRYSTNT SPRWPLAQPM RFLGHNGEIN TIQGNLNWMQ 377
AtFd-GOGAT2 ELYFSSLSNQ TIVYKGMLRS EVLGLFYPDL QNDLYKSPFA IYHRRFSTNT SPRWHLAQPM RFLGHNGEIN TIQGNLNWMT 353
NnFdGOGAT   ELYFCSLSNQ TIVYKGMLRS EVLGQFYSDL QSDLYKSPFA IYHRRYSTNT SPRWPLAQPM RLLGHNGEIN TIQGNLNWMQ 360
Consensus   ELYFCSLSNQ TIVYKGMLRS EVLGQFYLDL QNELYKSPFA IYHRRYSTNT SPRWPLAQPM RLLGHNGEIN TIQGNLNWMX

                     420         440         460         480
SpFd-GOGAT  SRETTIKSPV WRGRENEIRP YGNPKGSDSA NLDSAAELLL RSGRSPAEAL MILVPEAYKK HPTLSIKYPE VIDFYDYYKG 434
OsFd-GOGAT  SREATLQSPV WRGREHEIRP FGDPKASDSA NLDSTAELLL RSGRSPAEAM MILVPEAYKN HPTLSIKYPE VIDFYDYYKG 422
BdFd-GOGAT  SREATIQSPV WRGRENEIRP FGDPKASDSA NLDNAAELLL RSGRSPAEAM MMLVPEAYKN HPTLSIKYPE VIDFYDYYKG 415
SbFd-GOGAT  SRETTLQSPV WRGREHEICP FGDPKASDSA NLDSTAELLL RSGRSPAEAL MILVPEAYKN HPTLSIKYPE VIDFYDYYKG 424
AtFd-GOGAT1 SREASLKAAV WNGRENEIRP FGNPRGSDSA NLDSAAEIMI RSGRTPEEAL MILVPEAYKN HPTLSVKYPE VVDFYDYYKG 457
AtFd-GOGAT2 SREASLRSPV WHGRENDIRP ISNPKASDSA NLDSAAELLI RSGRTPEESL MILVPEAYKN HPTLMIKYPE AVDFYDYYKG 433
NnFdGOGAT   SRETSLKSPV WRGREDEICP YGNPKASDSA NLDSAAELLL RSGRSPEEAL MILVPEAYKN HPTLMIKYPE VVDFYEYYKG 440
Consensus   SREATLXSPV WRGRENEIRP FGNPKASDSA NLDSAAELLL RSGRSPAEAL MILVPEAYKN HPTLSIKYPE VIDFYDYYKG

                     500         520         540         560
SpFd-GOGAT  QMEAWDGPAL LLFSDGKTVG ACLDRNGLRP ARYWRTVDDV VYVASEVGVL PMDESKITMK GRLGPGMMIT VDLQNGQVYE 514
OsFd-GOGAT  QMEAWDGPAL LLFSDGRTVG ACLDRNGLRP ARYWRTSDDF VYVASEVGVI PMDESKVVMK GRLGPGMMIT VDLQTGQVLE 502
BdFd-GOGAT  QMEAWDGPAL LLFSDGRTVG ACLDRNGLRP ARYWRTSDGF VYVASEVGVI PMDESKVVMK GRLGPGMMIT VDLQTGQVLE 495
SbFd-GOGAT  QMEAWDGPAL LLFSDGRTVG ATLDRNGLRP ARYWRTSDDF VYVASEVGVI PMDESKVVMK GRLGPGMMIT VDLQTGQVLE 504
AtFd-GOGAT1 QMEAWDGPAL LLFSDGKTVG ACLDRNGLRP ARYWRTSDNF VYVASEVGVV PVDEAKVTMK GRLGPGMMIA VDLVNGQVYE 537
AtFd-GOGAT2 QMEPWDGPAL VLFSDGKTVG ACLDRNGLRP ARYWRTSDNV VYVASEVGVL PMDESKVTMK GRLGPGMMIS VDLENGQVYE 513
NnFdGOGAT   QMEAWDGPAL LLFSDGKTVG ACLDRNGLRP ARYWRTVDNV VYVASEVGVL PMDESRVTMK GRLGPGMMIT ADLLTGQVYE 520
Consensus   QMEAWDGPAL LLFSDGKTVG ACLDRNGLRP ARYWRTSDXF VYVASEVGVX PMDESKVTMK GRLGPGMMIT VDLQTGQVYE

                     580         600         620         640
SpFd-GOGAT  NTDVKKKVAS AHPYGQWLNE NMRSMKPVNF LSSPVMDDEL VLRHQQAFGY SSEDVQMVIE TMASQGKEPT FXMGDDIPLA 594
OsFd-GOGAT  NTEVKKSVAS ANPYGSWLQQ STRSIKPVNF QSSVAMDNET VLRHQQAFGY SSEDVQMVIE TMASQGKEPT FCMGDDIPLA 582
BdFd-GOGAT  NTEVKKNVAS AKPYGTWLQQ STRSIKPVNF QSSPVMDNET VMRHQQAFGY SSEDVQMVIE TMASQGKEPT FCMGDDIPLA 575
SbFd-GOGAT  NTEVKKTVAL ASPYGTWLQE RTRSIKPVNF LSSTIMDNET VLRHQQAFGY SSEDVQMVIE SMASQGKEPT FCMGDDIPLA 584
AtFd-GOGAT1 NTEVKKRISS FNPYGKWIKE NSRFLKPVNF KSSTVMENEE ILRSQQAFGY SSEDVQMVIE SMASQGKEPT FCMGDDIPLA 617
AtFd-GOGAT2 NTEVKKRVAS YNPYGKWVSE NLRNLKPSNY LSSAILETDE TLRRQQAFGY SSEDVQMVIE SMAAQGKEPT FCMGDDTPVA 593
NnFdGOGAT   NTDVKKRVAL SNPYGKWLSE NMRTLKPVNF LSASVMDKEI ILRHQQAFGY SSEDVQMVIE TMAAQGKEPT FCMGDDIPLA 600
Consensus   NTEVKKRVAS ANPYGKWLQE NTRSXKPVNF LSSXVMDNET VLRHQQAFGY SSEDVQMVIE TMASQGKEPT FCMGDDIPLA

                     660         680         700         720
SpFd-GOGAT  XLSQXPHMIY DYFKQRFAQV TNPAIDPLRE GLVMSLEVNL GKRGNILEVG PQNAAQVILS SPVLNEGELE TLMKDTALTP 674
OsFd-GOGAT  VLSQKPHMLF DYFKQRFAQV TNPAIDPLRE GLVMSLEVNI GKRRNILEVG PENADQVTLS SPVLNEGELE SLLNDSKLKP 662
BdFd-GOGAT  VLSQKPHMLY DYFKQRFAQV TNPAIDPLRE GLVMSLEVNI GKRGNILEVG PENADQVALS SPVLNEGELD SLLKDTKLKP 655
SbFd-GOGAT  VLSQRPHLLY DYFKQRFAQV TNPAIDPLRE GLVMSLEVNI GKRGNILEVG PENADLVALS SPVLNEGELE TLLKDPKLKP 664
AtFd-GOGAT1 GLSQRPHMLY DYFKQRFAQV TNPAIDPLRE GLVMSLEVNI GKRGNILELG PENASQVILS NPVLNEGALE ELMKDQYLKP 697
AtFd-GOGAT2 VLSQKPHMLY DYFKQRFAQV TNPAIDPLRE GLVMSLEVNI GKRGNILEVG PQNVSQVVLS GPVLNERELE GLLGDPLLKS 673
NnFdGOGAT   ALSQKPHMLF DYFKQRFAQV TNPAIDPLRE GLVMSLEVNI GKRGNILEVG PENASQVILS SPVLNEGELE LLMEDPYLKP 680
Consensus   VLSQKPHMLY DYFKQRFAQV TNPAIDPLRE GLVMSLEVNI GKRGNILEVG PENAXQVILS SPVLNEGELE XLLKDPKLKP
```

```
                                          740                                       760                                       780                                       800
SpFd-GOGAT   QVLPTFFDIG KGIDGSLQKA LEELCEAADE AVRNGSQLLI LSDRSEEIEP TRPAIPILLA VGAVHQHLIQ NGLRMSASIV 754
OsFd-GOGAT   KVLSTYFDIR KGLDGSLDKA IKVLCDEADA AVRNGSQLLV LSDRSEALEP TRPAIPILLA VGAIHQHLIQ NGLRMSASIV 742
BdFd-GOGAT   TVLSTYFSIR KGLDGSLDKA IKALCEEADA AVRSGSQLLV LSDRSEALEP TRPAIPILLA VGAIHQHLIQ NGLRMSASIV 735
SbFd-GOGAT   KVLSTYFDIR KGLDGSLDKT IQALCEEADA AVRSGSQLLV LSDRSEAPEP TRPAIPILLA VGAIHQHLIQ NGLRMSASIV 744
AtFd-GOGAT1  KVLSTYFDIR KGVEGSLQKA LYYLCEAADD AVRSGSQLLV LSDRSDRLEP TRPSIPIMLA VGAVHQHLIQ NGLRMSASIV 777
AtFd-GOGAT2  QILPTFFDIR RGIEGSLKKG LLKLCEAADE AVRNGSQVLV LSDRSDNPEP TRPAIPMLLA VGAVHQHLIQ NGLRMSASII 753
NnFdGOGAT    QVLPTFFDIR KGLDGSLEKT IKKLCEDADE AVRNGSQLLI LSDRSEELEP TRPAIPILLA VGSVHQHLIQ NGLRMSASIV 760
Consensus    XVLSTYFDIR KGLDGSLDKA IKXLCEXADX AVRNGSQLLV LSDRSEALEP TRPAIPILLA VGAVHQHLIQ NGLRMSASIV

                                          820                                       840                                       860                                       880
SpFd-GOGAT   ADTAQCFSTH QFACLIGYGA SAICPYLAFE TCRQWRLSTK TVNLMKNGKM PTVTMEQCQR NFSKAVKSGL LKILSKMGIS 834
OsFd-GOGAT   ADTAQCFSTH QFACLIGYGA SAICPYLALE TCRQWRLSNK TVNLMRNGKM PTVTIEQAQR NFIKAVKSGL LKILSKMGIS 822
BdFd-GOGAT   ADTAQCFSTH QFACLIGYGA SAICPYLALE TCRQWRLSNK TVNLMRNGKM PTVTIEQAQR NFIKAVKSGL LKILSKMGIS 815
SbFd-GOGAT   ADTAQCFSTH HFACLIGYGA SAVCPYLALE TCRQWRLSNK TVNLMRNGKM PTVTIEQAQR NFTKAVKLGL LKILSKMGIS 824
AtFd-GOGAT1  ADTAQCFSTH HFACLVGYGA SAVCPYLALE TCRQWRLSNK TVAFMRNGKI PTVTIEQAQK NYTKAVNAGL LKILSKMGIS 857
AtFd-GOGAT2  ADTAQCFSTH HFACLIGYGA SAICPHLALE TCRQWRLSNK TVNMMRNGKM PTVTMEQAQK NYRKAVNTGL LKVLSKMGIS 833
NnFdGOGAT    ADTAQCFSTH QFACLIGYGA SAVCPYLALE TCRQWRLSTK TVNLMRNGKM PTVTMEQAQR NFCKAVKSGL LKILSKMGIS 840
Consensus    ADTAQCFSTH QFACLIGYGA SAICPYLALE TCRQWRLSNK TVNLMRNGKM PTVTIEQAQR NFXKAVKSGL LKILSKMGIS

                                          900                                       920                                       940                                       960
SpFd-GOGAT   LLSSYCGAQI FEIYGLGHDI VDFAFCGSVS KIGGLNFDEL ARETLSFWVK AFSEDTAKRL ENFGFIQFRP GGEYHGNNPQ 914
OsFd-GOGAT   LLSSYCGAQI FEIYGLGQEV VDLAFCGSVS KIGGLTLDEL GRETLSFWVK AFSEDTAKRL ENFGFIQSRP GGEYHANNPE 902
BdFd-GOGAT   LLSSYCGAQI FEIYGLGQEV VDLAFCGSVS KIGGLTLDEL GRETLSFWVK AFSEDTAKRL ENFGFIQSRP GGEFHANNPE 895
SbFd-GOGAT   LLSSYCGAQI FEIYGLGQEV VDLAFCGSVS KIGGLTLDEL GRETLSFWVK AFSEDTAKRL ENFGFIQSRP GGEYHANNPE 904
AtFd-GOGAT1  LLSSYCGAQI FEIYGLGQDV VDLAFTGSVS KISGLTFDEL ARETLSFWVK AFSEDTTKRL ENFGFIQFRP GGEYHSNNPE 937
AtFd-GOGAT2  LFSSYCGAQI FEIYGLGNEV VEFSFRGSAS QIGGLTLDEL ARETLTFWVR AFSEDTAKRL ENFGFIQFRP GGEYHGNNPE 913
NnFdGOGAT    LLSSYCGAQI FEIYGLGKDI VDLAFCGSVS NIGGLTLDEL ARETLSFWVK AFSEDTAKRL ENFGFIQFRP GGEYHGNNPE 920
Consensus    LLSSYCGAQI FEIYGLGQEV VDLAFCGSVS KIGGLTLDEL ARETLSFWVK AFSEDTAKRL ENFGFIQFRP GGEYHXNNPE

                                          980                                     1,000                                     1,020                                     1,040
SpFd-GOGAT   MSKLLHKAVR QKSESAYAVY QQHLASRPVS VLRDLLEFKS DRAPIPVGKV EPASSIVQRF CTGGMSLGAI SRETHEAIAI 994
OsFd-GOGAT   MSKLLHKAVR EKSDNAYTVY QQHLASRPVN VLRDLLELKS DRAPIPIGKV EPATSIVERF CTGGMSLGAI SRETHEAIAI 982
BdFd-GOGAT   MSKLLHKAIR EKSDNAYTIY QQHLASRPVN VLRDLVELKS DRAPIPIGKV EPATSIVERF CTGGMSLGAI SRETHEAIAI 975
SbFd-GOGAT   MTKLLHKAIR EKRDNAYTVY QQHLASRPVN VLRDLLELKS DRAPIPIGKV EPATSIVERF CTGGMSLGAI SRETHEAIAI 984
AtFd-GOGAT1  MSKLLHKAVR EKSETAYAVY QQHLSNRPVN VLRDLLEFKS DRAPIPVGKV EPAVAIVQRF CTGGMSLGAI SRETHEAIAI 1017
AtFd-GOGAT2  MSKLLHKAVR EKSETAYAVY QQHLANRPIT VFRDLLEFKS DRNPIPVGKV EPASSIVERF CTGGMSLGAI SRETHETIAI 993
NnFdGOGAT    MSKLLHKAVR QKNESVYSIY QQHLANRPVN VLRDLLEFKS DRPPIPVGKV ESAASIVQRF CTGGMSLGAI SRETHEAIAI 1000
Consensus    MSKLLHKAVR EKSENAYXVY QQHLASRPVN VLRDLLEFKS DRAPIPVGKV EPATSIVERF CTGGMSLGAI SRETHEAIAI

                                        1,060                                     1,080                                     1,100                                     1,120
SpFd-GOGAT   AMNRLGGKSN SGEGGEDPIR WNPLTDVTDG YSPTLPHLKG LQNGDTATSA IKQVASGRFG VTPTFLVNAD QLEIKIAQGA 1074
OsFd-GOGAT   AMNRIGGKSN SGEGGEDPIR WSPLADVEDG YSPTLPHLKG LQNGDTATSA IKQVASGRFG VTPTFLVNAE QIEIKIAQGA 1062
BdFd-GOGAT   AMNRIGGKSN SGEGGEDPIR WSPLEDVVDG YSPTLPHLKG LQNGDTATSA IKQVASGRFG VTPTFLVNAE QIEIKIAQGA 1055
SbFd-GOGAT   AMNRIGGKSN SGEGGEDPIR WNPLTDVVDG YSPTLPHLKG LQNGDTATSA IKQVASGRFG VTPTFLVNAN QIEIKIAQGA 1064
AtFd-GOGAT1  AMNRIGGKSN SGEGGEDPIR WKPLTDVVDG YSPTLPHLKG LQNGDIATSA IKQVASGRFG VTPTFLVNAD QLEIKVAQGA 1097
AtFd-GOGAT2  AMNRLGGKSN SGEGGEDPIR WKPLTDVVDG YSSTLPHLKG LRNGDTATSA IKQVASGRFG VTPTFLVNAD QLEIKVAQGA 1073
NnFdGOGAT    AMNRLGGKSN SGEGGEDPIR WSPLTDVVDG YSPTLPHLKG LQNGDTATSA IKQVASGRFG VTPTFLVNAD QLEIKIAQGA 1080
Consensus    AMNRIGGKSN SGEGGEDPIR WSPLTDVVDG YSPTLPHLKG LQNGDTATSA IKQVASGRFG VTPTFLVNAD QLEIKIAQGA

                                        1,140                                     1,160                                     1,180                                     1,200
SpFd-GOGAT   KPGEGGQLPG KKVSAYIARL RNSKPGVPLI SPPPHHDIYS IEDLAQLIYD LHQVNPKAKV SVKLVAEAGI GTVASGVAKG 1154
OsFd-GOGAT   KPGEGGQLPG KKVSAYIARL RNSKPGVPLI SPPPHHDIYS IEDLAQLIYD LHQINPKAKV SVKLVAEAGI GTVASGVSKG 1142
BdFd-GOGAT   KPGEGGQLPG KKVSAYIARL RNSKPGVPLI SPPPHHDIYS IEDLAQLIFD LHQINPKAKV SVKLVAEAGI GTVASGVSKA 1135
SbFd-GOGAT   KPGEGGQLPG KKVSAYIARL RNSKPGVPLI SPPPHHDIYS IEDLAQLIYD LHQINPKAKV SVKLVSEAGI GTVASGVSKG 1144
AtFd-GOGAT1  KPGEGGQLPG KKVSAYIARL RSSKPGVPLI SPPPHHDIYS IEDLAQLIFD LHQINPNAKV SVKLVAEAGI GTVASGVAKG 1177
AtFd-GOGAT2  KPGEGGQLPG KKVSAYIARL RNSKPGVPLI SPPPHHDIYS IEDLAQLIFD LHQVNPKAKV SVKLVSETGI GTVASGVAKA 1153
NnFdGOGAT    KPGEGGQLPG KKVSAYIARL RNSKPGVPLI SPPPHHDIYS IEDLAQLIYD LHQVNPKAKV SVKLVAEAGI GTVASGVAKG 1160
Consensus    KPGEGGQLPG KKVSAYIARL RNSKPGVPLI SPPPHHDIYS IEDLAQLIYD LHQINPKAKV SVKLVAEAGI GTVASGVAKG

                                        1,220                                     1,240                                     1,260                                     1,280
SpFd-GOGAT   NADIIQISGH DGGTGASPVS SIKHAGGPWE LGLAETHQTL IANGLRERVI LRVDGGFKSG VDVLMAASMG ADEYGFGSVA 1234
OsFd-GOGAT   NADIIQISGH DGGTGASPIS SIKHAGGPWE LGLSETHQTL IQNGLRERVV LRVDGGFRSG LDVLMAAAMG ADEYGFGSVA 1222
BdFd-GOGAT   NADVIQISGH DGGTGASPIS SIKHAGGPWE LGLTETHQTL IQNGLRERVV LRVDGGFRSG LDVLLAAAMG ADEYGFGSVA 1215
SbFd-GOGAT   NADIIQISGH DGGTGASPIS SIKHAGGPWE LGLTETNQTL IQNGLRERVV LRVDGGFRSG RDVLMAAAMG ADEYGFGSVA 1224
AtFd-GOGAT1  NADIIQISGH DGGTGASPIS SIKHAGGPWE LGLTETHQTL IANGLRERVI LRVDGGFKSG VDVLMAAAMG ADEYGFGSLA 1257
AtFd-GOGAT2  NADIIQISGY DGGTGASPIS SIKHAGGPWE LGLAETQKTL IGNGLRERVI IRVDGGFKSG VDVLIAAAMG ADEYGFGTLA 1233
NnFdGOGAT    NADIIQISGH DGGTGASPIS SIKHAGGPWE LGLTETHQTL IENGLRERVI LRVDGGFKSG VDVLMAAAMG ADEYGFGSVA 1240
Consensus    NADIIQISGH DGGTGASPIS SIKHAGGPWE LGLTETHQTL IQNGLRERVI LRVDGGFKSG VDVLMAAAMG ADEYGFGSVA

                                        1,300                                     1,320                                     1,340                                     1,360
SpFd-GOGAT   MIATGCVMAR ICHTNNCPVG VASQREELRA RFPGVPGDLV NYFLYVAEEV RGILAQLGYE KMDDVIGRTD LLRPRHVSLM 1314
OsFd-GOGAT   MIATGCVMAR ICHTNNCPVG VASQREELRA RFPGVPGDLV NYFLFVAEEV RATLAQLGFE KLDDIIGRTD ILKAKHVSLA 1302
BdFd-GOGAT   MIATGCVMAR ICHTNNCPVG VASQREELRA RFPGVPGDLV NYFLFVAEEV RATLAQLGYE KLDDITGRTD LLKPKHISLV 1295
SbFd-GOGAT   MIATGCVMAR ICHTNNCPVG VASQREELRA RFPGVPGDLV NYFLFVAEEV RATLAQLGYE KLDDVIGRTD LLKPKHVSLV 1304
AtFd-GOGAT1  MIATGCVMAR ICHTNNCPVG VASQREELRA RFPGVPGDLV NYFLYVAEEV RGILAQLGYN SLDDIIGRTE LLRPRDISLV 1337
AtFd-GOGAT2  MIATGCIMAR ICHTNNCPVG VASQREELRA RFPGLPGDLV NFFLYIAEEV RGILAQLGYE KLDDIIGRTD LLKARDISLV 1313
NnFdGOGAT    MIATGCVMAR ICHTNNCPVG VASQREELRA RFPGVPGDLV NYFMYVAEEV RGILAQLGYE KMDDIIGRTD ILRPRNISLV 1320
Consensus    MIATGCVMAR ICHTNNCPVG VASQREELRA RFPGVPGDLV NYFLYVAEEV RGILAQLGYE KLDDIIGRTD LLKPRHISLV

                                        1,380                                     1,400                                     1,420                                     1,440
SpFd-GOGAT   KTQQIDLSYL LSNVGLPKWS STEIRNQPVH TNGPVLDDVL LSDPEITEAI EKEKVVNKTI KIYNVDRAVC GRIAGVIAKK 1394
OsFd-GOGAT   KTQHIDLKYL LSSAGLPKWS SSQIRSQDVH SNGPVLDETI LADPDISDAI ENEKEVSKTF QIYNVDRAVC GRVAGVIAKK 1382
BdFd-GOGAT   KTQHIDLGYL LMNSGLPKWS SSQIRSQDVH SNGPVLDETI LADPEVSDAI ENEKEVSKTF PIYNVDRAVC GRVAGAIAKK 1375
SbFd-GOGAT   KTQHIDLGYL LSNAGLPKWS SSQIRSQDVH TNGPVLDETI LADPEIADAI ENEKEVSKAF QIYNVDRAVC GRVAGVIAKK 1384
AtFd-GOGAT1  KTQHLDLSYL LSSVGTPSLS STEIRKQEVH TNGPVLDDDI LADPLVIDAI ENEKVVEKTV KICNVDRAAC GRVAGVIAKK 1417
AtFd-GOGAT2  KT-HLDLSYL LSSVGLPKRS STSIRKQEVH SNGPVLDDTL LQDPEIMDAI ENEKTVHKTM SIYNVDRSVC GRIAGVIAKK 1392
NnFdGOGAT    KTQHLDLSYI LSSVGLPKLS STKIRNQDVH TNGPVLDDVI LSDPEISDAI ENEKVVNKTI KIYNVDRAVC GRIAGVVAKK 1400
Consensus    KTQHIDLSYL LSSVGLPKWS STQIRSQDVH TNGPVLDDTI LADPEISDAI ENEKXVSKTF KIYNVDRAVC GRVAGVIAKK
```

```
                  1,460                1,480                1,500                1,520
SpFd-GOGAT  YGDTGFAGQL NITFMGSAGQ SFGCFLTPGM NIRLVGEAND YVGKGMAGGE LVLTPPENFG FCPEEATIVG NTCLYGATGG 1474
OsFd-GOGAT  YGDTGFAGQL NITFTGSAGQ SFGCFLTPGM NIRLVGEAND YVGKGMAGGE LVVVPVEKTG FVPEDAAIVG NTCLYGATGG 1462
BdFd-GOGAT  YGDTGFAGQL NITFTGSAGQ SFGCFLTPGM NVRLVGEAND YVGKGMAGGE LVVVPVDDTG FVPEEAAIVG NTCLYGATGG 1455
SbFd-GOGAT  YGDTGFAGQL NITFNGSAGQ SFGCFLTPGM NIRLVGEAND YVGKGMAGGE LVVVPVDKTG FVPEDATIVG NTCLYGATGG 1464
AtFd-GOGAT1 YGDTGFAGQV NLTFLGSAGQ SFGCFLIPGM NIRLIGESND YVGKGMAGGE IVVTPVEKIG FVPEEATIVG NTCLYGATGG 1497
AtFd-GOGAT2 YGDTGFAGQL NLTFTGSAGQ SFACFLTPGM NIRLVGEAND YVGKGMAGGE VVILPVESTG FRPEDATIVG NTCLYGATGG 1472
NnFdGOGAT   YGDTGFAGQL NITFTGSAGQ SFACFLTPGM NIRLIGEAND YVGKSMAGGE LVVTPVENTG FCPEDATIVG NTCLYGATGG 1480
Consensus   YGDTGFAGQL NITFTGSAGQ SFGCFLTPGM NIRLVGEAND YVGKGMAGGE LVVXPVEKTG FVPEDATIVG NTCLYGATGG

                  1,540                1,560                1,580                1,600
SpFd-GOGAT  QLFVRGKAGE RFAVRNSLTE AVVEGTGDHC CEYMTGGCVV VLGKVGRNVA AGMTGGLAYI LDEDDTLLPK VNREIVRMQR 1554
OsFd-GOGAT  QVFVRGKTGE RFAVRNSLGQ AVVEGTGDHC CEYMTGGCVV VLGKVGRNVA AGMTGGLAYI LDEDDTLVPK VNKEIVKMQR 1542
BdFd-GOGAT  QVFVRGKTGE RFAVRNSLGQ AVVEGTGDHC CEYMTGGCVV VLGKVGRNVA AGMTGGLAYI LDEDDTLVPK VNKEIVKMQR 1535
SbFd-GOGAT  QVFVRGKAGE RFAVRNSLCQ AVVEGTGDHC CEYMTGGCVV VLGKAGRNVA AGMTGGLAYI LDEDDTLLPK VNKEIVKMQR 1544
AtFd-GOGAT1 QIFARGKAGE RFAVRNSLAE AVVEGTGDHC CEYMTGGCVV VLGKVGRNVA AGMTGGLAYL LDEDDTLLPK INREIVKIQR 1577
AtFd-GOGAT2 LLFVRGKAGE RFAVRNSLAQ AVVEGTGDHC CEYMTGGCVV ILGKVGRNVA AGMTGGLAYI LDEDNTLLPK MNKEIVKIQR 1552
NnFdGOGAT   QVFVRGKAGE RFAVRNSLAQ AVVEGTGDHC CEYMTGGCVV VLGKVGRNVA AGMTGGLAYI LDEDDTLIPK VNKEIVKIQR 1560
Consensus   QVFVRGKAGE RFAVRNSLAQ AVVEGTGDHC CEYMTGGCVV VLGKVGRNVA AGMTGGLAYI LDEDDTLLPK VNKEIVKMQR

                  1,620                1,640                1,660
SpFd-GOGAT  VNAPVGQMQL KSLIEAHVEK TGSTKGATIL REWEAYLPLF WQIVPPSEED TPEASAEIQR V-PT-K---- EMQSA---- 1623
OsFd-GOGAT  VNAPAGQMQL KGLIEAYVEK TGSEKGATIL REWEAYLPLF WQLVPPSEED SPEACAEFER VLAKQA---T TVQSAK--- 1615
BdFd-GOGAT  VNAPAGQMQL KGLIEAYVEK TGSVKGAKIL SEWEAYLPLF WQLVPPSEED SPEACAEFER VLARQA---T AVQSAK--- 1608
SbFd-GOGAT  VNAPAGQMQL KSLIESYVEK TGSEKGATIL REWEAYLPLF WQLVPPSEED SPEACAEFER VLAKQAKQAT TQLSAK--- 1620
AtFd-GOGAT1 VTAPAGELQL KSLIEAHVEK TGSSKGATIL NEWEKYLPLF WQLVPPSEED TPEASAAYVR TSTGEV---- TFQSA---- 1648
AtFd-GOGAT2 VTSPVGQTQL KSLIQAHVEK TGSSKGAMIV EEWDKYLAMF WQLVPPSEED TPEANSD--H ILKTTTGDEE QVSSTLAEK 1629
NnFdGOGAT   VNAPAGQIQL KSLIEAHVEK TGSNKGSAIL KDWEAYLPLF WQLVPPSEED TPEACADFER ISPGQV---- TLQKA---- 1631
Consensus   VNAPAGQMQL KSLIEAHVEK TGSXKGATIL REWEAYLPLF WQLVPPSEED TPEACAEFER VLAXQA---T TVQSAK---
```

B

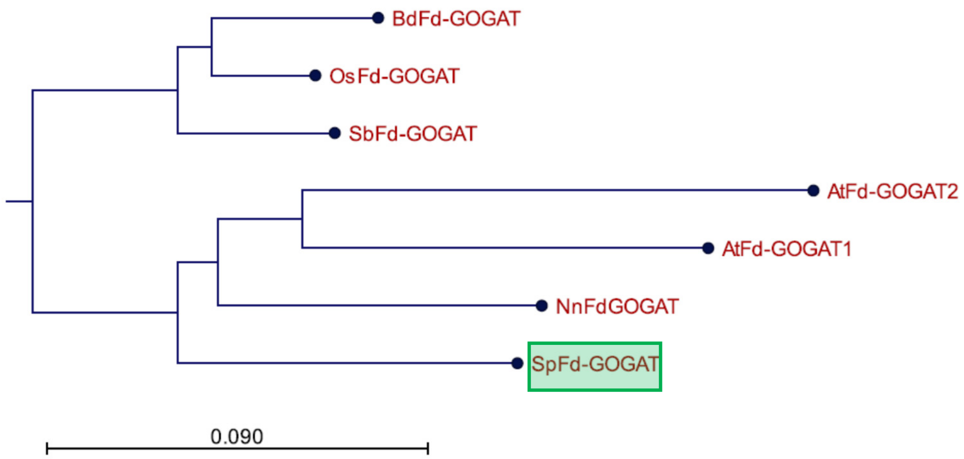

Figure S11. Sequence alignment of Fd-GOGAT protein from *S.polyrhiza* with other representative species **(A)** and the resulted phylogenetic tree **(B)**. AtFd-GOGAT1 - *A. thaliana*, NP_850763.1, AtFd-GOGAT2 - *A. thaliana*, NP_181655.1, BdFd-GOGAT - *B. distachyon*, XP_003559858.1, NnFd-GOGAT - *N. nucifera*, XP_010276670, OsFd-GOGAT - *O. sativa*, XP_015646712.1, SbFd-GOGAT - *S. bicolor*, XP_002463318.2, SpFdGOGAT - *S. polyrhiza* (sequence translated from Acc. ID MZ605910)

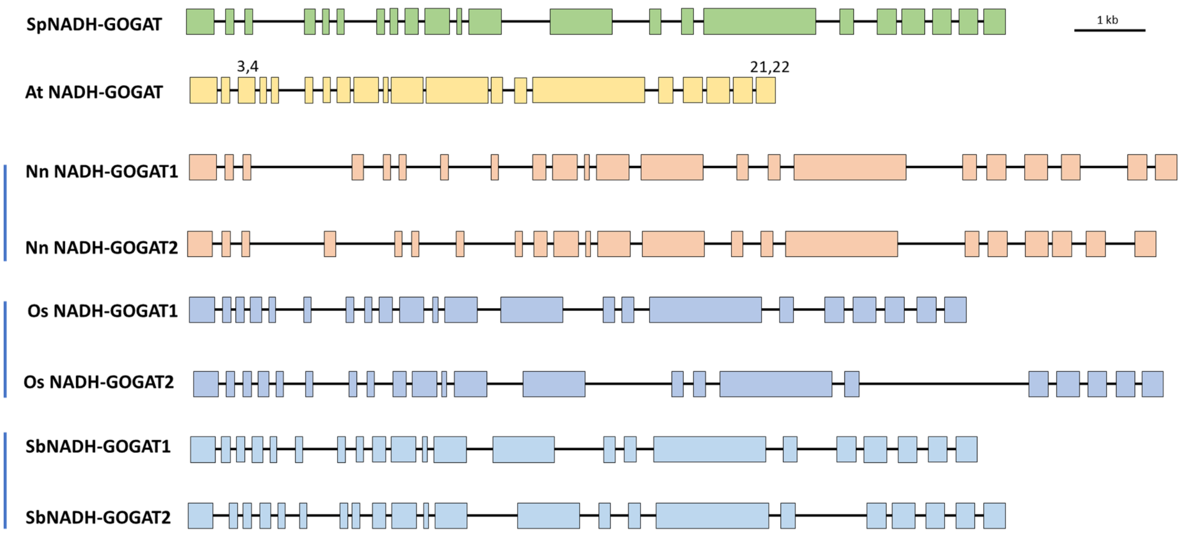

Figure S12. Diagrammatic representation of the structure of *NADH-GOGAT* genes. Coding sequences are colored boxes. Introns are black lines. The bar is 1 kb. *At NADH-GOGAT - A. thaliana,* At5g53460, *NnNADH-GOGAT1 - N. nucifera,* LOC104600364, *NnNADH-GOGAT2 - N. nucifera,* LOC104604004, *OsNADH-GOGAT1 - O. sativa,* LOC4324398, *OsNADH-GOGAT2 - O. sativa,* LOC4339561, *SbNADH-GOGAT1 - S. bicolor,* LOC8068026, *SbNADH-GOGAT - S. bicolor,* LOC8074568, *SpNADH-GOGAT – S. polyrhiza* CP019094, the 8th chromosome, position from 4978991 to 4990379.

A

```
                                           20                    40                    60                    80
SpNADH-GOGAT   MAALPGSSLQ ILSDSSLQIL SDSPVLPSVT PRKDVRALGR RNGSRARRCP AAERSGRDVV NVENKFFGER LRAK-----E 75
AtNADH-GOGAT   MSAASSSSVL HLRTNQQLLS LRSLKNSTSV ASQLAVTSG- ---VSRRRSC TARCSVKKPV IPESPFLGTR VRRSG---SE 73
NnNADH-GOGAT1  MSAVPGSA-- ------FQLQ TKSVVLPSRN SPSLSH-RGW NVAAPLSRGT SSCSAKTRRN AVENKFFGTR LRQLG---PE 68
NnNADH-GOGAT2  MAAIPGSS-- ------FQLR NKSVGLPSPG RPSLKNQRNF VPFSSRESKA SCCSARTRHN VVEKKFFGTG LRQSG---PE 69
OsNADH-GOGAT1  MSAAQGMA-- ------YKLR TDAAPTGAGR RARRSHSSVA APYRAARLVQ GGVSIEGG-L VGGCQL--TE ERVAARP--P 67
SbNADH-GOGAT1  MSTAQGTA-- ------LK-- --SAPMGPGR RARRGRP-VA APYRSGWQAY GGVSLEGGGF LGGVQR--TE ERVAPRA--P 63
BdNADH-GOGAT1  MPTAQG---- --------IG LKHAAPPGAG RRARRSQSAS APCRSTRQAH GGMSLEGGFV GGTQP---TE GRVAPRP--P 63
OsNADH-GOGAT2  MSAAQGLA-- ------LKLR AAPAAGGVRG EKRR-RAASA TAAAAARPRH GAMSLEGG-F LGGA--LPAE DRVAP----- 63
SbNADH-GOGAT2  MSAAQALA-- ------LKLR AAPAPGRPRG CRTTVRAAAA AHVGALQHRR CVSLAQGGRS LRGAALLAAQ QRPRPLPWAE 72
BdNADH-GOGAT2  MSAAQGLA-- ------LKLR AAPASGGRRA PRGRIFAA-C GPASRQRYGS GGVSLDGGFL RLPAAVARAA PRAPRASSSD 71
Consensus      MSAAQGSA-- ------LKLR AXSAPXXSRG RRRRXRAXGA APAXSARRXH GGVSLEGGXV LGXAKFXGTE LRVAP----E

                                          100                   120                   140                   160
SpNADH-GOGAT   RSHQWRSAGP GHYPKLSVVG PSM-SFSQVP EKNLGLYNPS FDKDACGVGF VAELSGDYSR KTV-TDAVEM LVRMAHRGAC 153
AtNADH-GOGAT   TLQFWRSDGP GRSAKLRTVV KS--SFSAVP EKPLGLYDPS YDKDSCGVGF VAELSGETTR KTV-TDSLEM LIRMTHRGAC 150
NnNADH-GOGAT1  RLHLWRSDGP GRSPKLRVVV RS--AFSQVP EKPLGLYDPS FDKDSCGVGF VAELSGETSR KTV-TDALEM LIRMSHRGAC 145
NnNADH-GOGAT2  RLHLWRSDGP GRSPKLRVVV RS--ALSKVP DKPLGLYDPS FDKDSCGVGF VAELSGETSR KTV-TDALEM LIRMSHRGAC 146
OsNADH-GOGAT1  RAAARD---- ---------A EPVRPLSTLP ESSIGLYDPS RERDSCGVGF VAELSGDYKR ATV-NDALEM LERMAHRGAC 133
SbNADH-GOGAT1  RAAARD---- ---------A EVVRPLSKLP NSSIGLYNPS FERDACGVGF VAELSGDYKR ETV-NDAIEM LERMAHRGAC 129
BdNADH-GOGAT1  RAAARD---- ---------A EPIRPLSKLP ESSIGLYNPA FERDSCGVGF IAELSGDENR ETV-NDAIQM LERMAHRGAC 129
OsNADH-GOGAT2  RASASRQAEA G--AGAGAAR PPPRSMSKIP ESSIGLYDPS MERDSCGVGF IAELSGEYSR KTV-DDAIEM LDRMAHRGAC 140
SbNADH-GOGAT2  RASARRHDGV GVVASASSAA AGVRSMSRIP KGNTGLYDPS MDRDSCGVGF IAELSAQPSR KTI-VDAIEM LERMSHRGAC 151
BdNADH-GOGAT2  FAAEEGAEAP ---------- LPPWSMSKIP ESSIGLYDPS FERDSCGVGF IAELSAEYSR KTVV-DAIEM LERMAHRGAC 140
Consensus      RAAAXRSDGP G---KL--VA EXVRSLSKVP ESSIGLYDPS FXRDSCGVGF VAELSGEYSR KTV-TDAIEM LERMAHRGAC

                                          180                   200                   220                   240
SpNADH-GOGAT   GCEKNTGDGA GILVALPHSF FAETTKDLGF ELPPAGEYAV GMFFLPTSES RREESKTVFA KVAESLGHVV LGWRSVPTNN 233
AtNADH-GOGAT   GCESNTGDGA GILVGLPHDF YAEAATELGF VLPSAGNYAV GMFFLPTVES RREESKNVFT KVAESLGHSV LGWRLVPTDN 230
NnNADH-GOGAT1  GCETNTGDGA GILVALPHGF FKEVAMDVGF ELPPPGEYAV GMFFLPTSET RREESKTVFT KVAESLGHVV LGWRSVPTDN 225
NnNADH-GOGAT2  GCETNTGDGA GILVALPHGF FTEVAKQVGF ELPPPGEYAV GMFFLPTSDT RREESKKVFT KVAESLGHVV LGWRSVPTDN 226
OsNADH-GOGAT1  GCEKNTGDGA GILVALPHNF FREVTKDAGF ELPQPGEYAV GMVFLPIDEK RRERSKAEFQ KVAESLGHVI LGWRRVPTDN 213
SbNADH-GOGAT1  GCEKNTGDGA GIMVALPHDF FKEVTKDAGF ELPPPGEYAV GMFFMPTDEK RREKGKAEFK KVAESLGHVI LGWRPVPTDN 209
BdNADH-GOGAT1  GCEKNTGDGA GILVALPHKF FREVTKDAGF ELPPPGEYAV GMVFLPTDEK RRDRSKAEFK KVAESLGHSI LGWRQVPTDN 209
OsNADH-GOGAT2  GCEKNTGDGA GILVALPHNF FREVTKDAGF ELPPPGEYAV GMFFMPTDDK RREKSKLLFR EKAELLGHTV LGWRRVPTDN 220
SbNADH-GOGAT2  GCEKNTGDGA GILVALPDAF FREVTKDAGF ELPPLGEYAV GMFFMPLDDE RREKSKLVFH EIAKSLGHVV LGWRRVPTDN 231
BdNADH-GOGAT2  GCEKNTGDGA GILVALPHEF FRKVVKDAGF ELPSPGDYAV GMFFMPRDDQ LREKSKLVFR EIAEKLGHVV LGWRRVPTDN 220
Consensus      GCEKNTGDGA GILVALPHXF FREVTKDAGF ELPPPGEYAV GMFFLPTDEK RREXSKXVFT KVAESLGHVV LGWRRVPTDN

                                          260                   280                   300                   320
SpNADH-GOGAT   SGLGKSAVQT EPIIEQVFLT PSPRSNIDME QQMYILRRVS MVAIRAALNM QHGGVKDFYI CSLSSRTIVY KGQLRPDQLK 313
AtNADH-GOGAT   SGLGNSALQT EPIIAQVFLT PTTKSKADFE QQMYILRRVS MVAIRAALNL QHGAMKDFYI CSLSSRTIVY KGQLKPDQLK 310
NnNADH-GOGAT1  TGLGKSALQT EPVIEQVFLT PSPRSKSDFE QQMYILRRVS MVAIRAALNL QHGGVRDFYI CSLSSRTIVY KGQLKPDQLQ 305
NnNADH-GOGAT2  TGLGKSALQT EPVIEQVFLT PSSRSKAGFE QQMYILRRVS MVAIRAALNL QHGGVRDFYI CSLSSRTIVY KGQLKPDQLK 306
OsNADH-GOGAT1  SDLGESALQT EPVIEQVFLT KSSSSEADFE QQLYILRRLS ILSIRAALNL RRGGKRDFYM CSLSSRTIVY KGQLKPCQLK 293
SbNADH-GOGAT1  SDLGESALET EPVIEQVFIT KSSRSEAEFE QQLYILRRLS IISVRAALNI KRGGERDFYM CSLSSRTIVY KGQLKPCQLK 289
BdNADH-GOGAT1  SDLGESALDT EPAIEQVFLT KSSKSKADFE QQLFILRRLS IISIRAALNL RRGGERDFYM CSLSSRTVVY KGQLMPSQLK 289
OsNADH-GOGAT2  SGLGQSAVDT EPVIEQVFVT KSASSKADFE RQMYVLRRFS VMSIREVLGV KNGGTKDFYM CSLSSRTIVY KGQLKPSQLK 300
SbNADH-GOGAT2  SDLGKAALET EPMIEQVFVS KSIHSKADIE QQMYILRGLS IKSIHEALGL EHGGPNDFYM CSLSSRTVVY KGQLKPSQLK 311
BdNADH-GOGAT2  LDLGKSALDT EPVIEQVFVT KSQRSEAEFE QQMYILRRFS IVSIREALGA QKG-QKNFYM CSLSSRTIVY KGQLMPSQLK 299
Consensus      SXLGKSALQT EPVIEQVFLT KSSRSKADFE QQMYILRRXS IVSIRAALNL QHGGVRDFYM CSLSSRTIVY KGQLKPXQLK

                                          340                   360                   380                   400
SpNADH-GOGAT   DYYYADVGNE RFTSYMALIH SRFSTNTFPS WDRAQPMRVL GHNGEINTLR GNKNWMKARE GLLKCKQLSL SKNELKKLLP 393
AtNADH-GOGAT   DYYYADLGSE RFTSYMALVH SRFSTNTFPS WDRAQPMRVL GHNGEINTLR GNVNWMRARE GLLKCNELGL SKKELKKLLP 390
NnNADH-GOGAT1  DYYYADLGNE RFTSYMALIH SRFSTNTFPS WDRAQPMRVL GHNGEINTLR GNINWMKARE GLLRCKKLGL SKNEMKKLLP 385
NnNADH-GOGAT2  DYYYADLGDE RFTSYMALIH SRFSTNTFPS WDRAQPMRIL GHNGEINTLR GNINWMKARE GLLKCRELGL SKNEMKKLLP 386
OsNADH-GOGAT1  GYYYADLGHE NFTSYMALVH SRFSTNTFPS WDRAQPMRVL GHNGEINTLK GNKNWMKARE GLLECEKLGL TKDQFSKILP 373
SbNADH-GOGAT1  GYYYADLGHE NFTSYMALVH SRFSTNTFPS WDRAQPMRVL GHNGEINTLR GNKNWITARE GLLEAEKLGL SKEQLSRILP 369
BdNADH-GOGAT1  GYYYADIGHE NFTSYMALVH SRFSTNTFPS WDRAQPMRVL GHNGEINTLR GNKNWMKARE GLLKCEKLGL SEDEMSKILP 369
OsNADH-GOGAT2  GYFFADLGDE SFTSYMALIH SRFSTNTFPS WDRAQPMRVL GHNGEINTLR GNKNWMKARE GLLKCEGLGL TRDEMLKLLP 380
SbNADH-GOGAT2  GYFFADLGDQ RFTSYMALVH SRFSTNTFPS WDRAQPMRVL GHNGEINTLR GNWNWMTARE GLLKCKGLGL SRDEMSKLLP 391
BdNADH-GOGAT2  GYFFADLGDE SFTSYMALVH SRFCTNTFPS WDRAQPMRIL GHNGEINTLR GNKNWMKARE GLLKCKGFGL SRDEMSKLLP 379
Consensus      GYYYADLGDE RFTSYMALVH SRFSTNTFPS WDRAQPMRVL GHNGEINTLR GNKNWMKARE GLLKCXKLGL SKDEMSKLLP

                                          420                   440                   460                   480
SpNADH-GOGAT   IVDASSSDSG AFDGVLELLI RSGRSLPEAV MMMIPEAWQN DKNMDPERKA LYEYFSALME PWDGPALISF TDGRYLGATL 473
AtNADH-GOGAT   IVDVSSSDSG AFDGVLELLV RAGRSLPEAV MMMIPEAWQN DKNIDPSRKE FYEYLSALME PWDGPALISF TDGRYLGATL 470
NnNADH-GOGAT1  IVDASSSDSG AFDGVLELLV RAGRSLPEAI MMMIPEAWQN DKNMDPDRKA LYEYFSAVME PWDGPALISF TDGRYLGATL 465
NnNADH-GOGAT2  IVDASSSDSG AFDGVLELLV RAGRSLPEAV MMMIPEAWQN DQNMDPDRKA LYEYFSALME PWDGPALISF TDGRYLGATL 466
OsNADH-GOGAT1  IVDATSSDSG AFDGVLELLI RGGRSLPEAV MMMIPEAWQN DVNMEPEKKA LYEFLSALME PWDGPALISF TDGRYLGATL 453
SbNADH-GOGAT1  IVDATSSDSG AFDNVLELLV RGGRSLPEAV MMMIPEAWQN DGNMDPEKKA LYEFLSALME PWDGPALISF TDGRYLGATL 449
BdNADH-GOGAT1  IVDATSSDSG AFDGVLELLI RGGRSLPEAV MMMIPEAWQN DANMQPEKKA LYEFLSALME PWDGPALISF TDGRYLGATL 449
OsNADH-GOGAT2  IVDATSSDSG AIDNVLELLI QSGRSAPEAV MMMIPEAWQN DVNMDPERKA LYEFFSALME PWDGPALISF TDGRYLGATL 460
SbNADH-GOGAT2  IVDPTSSDSG AFDNVLELLI RSGRSLPEAV MMMIPEAWHN DVNMDPERKA LYEYFSALME PWDGPALVSF TDGHYLGATL 471
BdNADH-GOGAT2  IVDSTSSDSG AFDNVLELLV QSGRSVAEAV MMMIPEAWQN NVDVDPERKA LYEYFSALME PWDGPALVSF TDGRYLGATL 459
Consensus      IVDATSSDSG AFDGVLELLX RSGRSLPEAV MMMIPEAWQN DVNMDPERKA LYEYFSALME PWDGPALISF TDGRYLGATL

                                          500                   520                   540                   560
SpNADH-GOGAT   DRNGLRPGRF YITHSGRVIM ASEVGVVDIP PEDVSKKGRL NPGMMLLVDF EHHTVVDDEA LKKQYSQARP YGEWLKRQKM 553
AtNADH-GOGAT   DRNGLRPGRF YITHSGRVIM ASEVGVVDVP PEDVMRKGRL NPGMMLLVDF EKHIVVDDDA LKQQYSLARP YGEWLKRQKI 550
NnNADH-GOGAT1  DRNGLRPGRF YVTHSGRVIM ASEVGVVDIP PEDICRKGRL NPGMMLLVDF EKHIVVDDEA LKRQYSLARP YGEWLSRQKI 545
NnNADH-GOGAT2  DRNGLRPGRY YITHSGRVIM ASEVGVVDIP PEDVCKKGRL NPGMMLLVDF EKHIVVDDAA LKKQYSLARP YGEWL-RKKI 545
OsNADH-GOGAT1  DRNGLRPGRF YVTHSGRVVM GSEVGVVDVP SKDVLRKGRL NPGMMLLVDF ENHTVVDDEA LKAQYSKAHP YGEWLKRQKI 533
SbNADH-GOGAT1  DRNGLRPGRF YVTHSGRVIM GSEVGVVDVP PEDVLRKGRL NPGMMLLVDF ENHTVVDDEA LKTQYSKAHP YGEWLKRQKI 529
BdNADH-GOGAT1  DRNGLRPGRF YVTHSGRVVM GSEVGVVDIP PQDVLRKGRL NPGMMLLVDF ENHTVVDDEA LKAQYSKAHP YGEWLKRQKI 529
OsNADH-GOGAT2  DRNGLRPGRF YVTYSGRVIM ASEVGVVDVP PQDVSRKGRL NPGMMLLVDF ENHCVVNDDE LKKEYSKVRP YGEWLKRQRI 540
SbNADH-GOGAT2  DRNGLRPGRF YVTYSGRVIM ASEVGVVDVP NDDVMRKGRV KPGMILLVDF EKHCIVDDDE LKKQYSRARP YGEWLERQKI 551
BdNADH-GOGAT2  DRNGLRPGRF YETYSGRVIM ASEVGVVDVL PEDVMTKGRL NPGMMLLIDF EKHCVVDDDA LKKQYAKAHP YGEWLKTQKI 539
Consensus      DRNGLRPGRF YVTHSGRVIM ASEVGVVDVP PEDVXRKGRL NPGMMLLVDF EKHTVVDDEA LKKQYSKARP YGEWLKRQKI
```

```
                                  580                                     600                                     620                                     640
SpNADH-GOGAT   CLEDVLSSVP ETDRLSPPIS GVVQADSHDD SMENVGVRGL LQPLKAFGYT VEALEMLLVP MVKDATEALG SMGNDTPLAV 633
AtNADH-GOGAT   ELKDIIESVP EAERIAPSIS GVVPASNDDD SMESMGIHGL LSPLKAFGYT VEALEMLLLP MAKDGSEALG SMGNDTPLAV 630
NnNADH-GOGAT1  ELKDIVDSVH EADRVPPAIS GSVPASSHDE NMENMGIHGL VAPLKAFGYT VEALEMLLLP MAKDATEALG SMGNDTPLAV 625
NnNADH-GOGAT2  ELKDIVNSVH ESDRVPPAIS GAVPASSHDD NMENMGIHGL LAPLKSFGYT VEALEMLLLP MAKDGTEALG SMGNDTPLAV 625
OsNADH-GOGAT1  YLKDIVESVP ETERVAPGIS GSLTQKN--E KKEHAGVNGI VTPLKAFGYT VEALEMLLLP MAKDGVEALG SMGNDTPLAV 611
SbNADH-GOGAT1  HLKDIVESVP ETDRVAPSIS SSLPQKN--E NKDDVGINGI LTPLKAFGYT VEALEMLLLP MAKDGVEALG SMGNDTPLAV 607
BdNADH-GOGAT1  YLKDIVESVP ETDRVAPSIA GSIPQMN--D NKECKGINGI VTPLKAFGYT VEALEMLLLP MAKDGVEALG SMGNDAPLAV 607
OsNADH-GOGAT2  QLTDIIESVN EAERIAPSIS GALPITK--E NKADMGICGI LTPLKAFGYT REALEMLMLP MAKDGQEALG SMGNDTPLAV 618
SbNADH-GOGAT2  QLADIIESVP ETERGAPRID -MLPQKN--E NKEAFCIHGI LAPLKAFGYT VETLEMLLLP MAKNGVEALG SMGNDTPLAV 628
BdNADH-GOGAT2  ELRDIIESVS ATERIAPMIS GAL------- -REPVGVNGI LAPLKAFGYT LETLDMLLLP MAKDGAEALG SMGNDTPLAV 611
Consensus      ELKDIVESVP ETXRVAPSIS GSLPXXN--E NKENMGIXGI LXPLKAFGYT VEALEMLLLP MAKDGVEALG SMGNDTPLAV
```

```
                                  660                                     680                                     700                                     720
SpNADH-GOGAT   MSDRAKIPSE YFKQMFAQVT NPPIDPIREK IVTSMECMIG PEGDLTETTE EQCHRLALKG PLLSIGEMEA IKKMNFQGWR 713
AtNADH-GOGAT   MSNREKLCFE YFKQMFAQVT NPPIDPIREK IVTSMECMIG PEGDLTETTE EQCHRLSLKG PLLKIEEMEA IKKMNYRGWR 710
NnNADH-GOGAT1  MSNREKLTFE YFKQMFAQVT NPPIDPIREK IVTSMECMIG PEGDLTETTE QQCHRLSLKG PLLSIEEMEA IKKMNYRGWR 705
NnNADH-GOGAT2  MSNREKLPFE YFKQMFAQVT NPPIDPIREK IVTSMECMIG PEGDLTETTE EQCHRLSLKG PLLSMDEMEA IKKMNYRGWR 705
OsNADH-GOGAT1  MSNREKLTFE YFKQMFAQVT NPPIDPIREK IVTSMECMIG PEGDLLETTE KQCNRLALEG PLVSIDEMEA IKKMNYRGWR 691
SbNADH-GOGAT1  MSNREKLTFE YFKQMFAQVT NPPIDPIREK IVTSMECMIG PEGDLLETTE KQCNRLALKG PLVSIDEMEA IKKMDYRGWR 687
BdNADH-GOGAT1  MSNREKLTFE YFKQMFAQVT NPPIDPIREK IVTSMECMIG PEGDLLEITE KQCNRLAIKG PLVSVDEMES IKKMDYRGWR 687
OsNADH-GOGAT2  MSNREKLTFE YFKQMFAQVT NPPIDPIREK IVTSMECMIG PEGDLSETTE RQCHRLTLKS PLLNTNEMEA IKKMNYRGWR 698
SbNADH-GOGAT2  MSNREKMPFD YFKQMFAQVT NPPIDPIREK IVTSMECMIG PEGDLSETTE HQCHRLKLQG PLLHIDEMEA IKKMNFRGWR 708
BdNADH-GOGAT2  MSNREKLTAE YFKQMFAQVT NPPIDPIREK IVTSMECMVG PEGDLSETTE RQCHRLTLKS PLLHIDEIEA IKNMNYRGWR 691
Consensus      MSNREKLTFE YFKQMFAQVT NPPIDPIREK IVTSMECMIG PEGDLTETTE XQCHRLALKG PLLSIDEMEA IKKMNYRGWR
```

```
                                  740                                     760                                     780                                     800
SpNADH-GOGAT   SKVLDITYSK NRGRKGLEET LDRICSEARA AIREGYTLLV LSDRGFSSER VGVSSLLAVG AVHQHLVSKL ERTRIGLIVE 793
AtNADH-GOGAT   TKVLDITYAK ERGTKGLEET LDRICDEANE AIKEGYTLLV LSDRAFSATR VAVSSLMAVG AVHHHLVKTL ARTQVGLVVE 790
NnNADH-GOGAT1  SKVLDITYLK SRGRKGLEEM LDRICSEAHE AIKEGFTILV LSDRAFSPNR VAVSSLLAIG AVHHHLVEKL ERTRIGLIVE 785
NnNADH-GOGAT2  SKVLDITYPK SRGMKGLEET LDRICSEARD ALKEGYTTLV LSDRAFSPNR VAVSSLLAVG AVHHHLVSKL ERTRIGLIVE 785
OsNADH-GOGAT1  SKVLDITYPK KSGRKGLEET LDRICTEARG AIKKGYTVLV LSDRGFSSDR VAVSSLLAVG AVHQHLVANL ERTRVGLLVE 771
SbNADH-GOGAT1  SKVLDITYPK KSGRKGLEEA LDRICAEARE AISKGYTILV LSDRGFSSDR VATSSLLAVG AVHQHLVANH ERTRIGLLVE 767
BdNADH-GOGAT1  SKVLDITYPK KSGRKGLEDT LDRICAEARE AIRNGYKILV LSDRGFSSDR VAVSSLLAVG AVHQHLVANL ERTRVGLLVE 767
OsNADH-GOGAT2  SKVLDITYPK KNGRMGLKQT LDKICAQARE AIHEGYTILV LSDRGFSSER VAVSSLLAVG AVHQHLVSHL ERTRIGLLVE 778
SbNADH-GOGAT2  SKVLDITYPK VYGRQGLEQT LDKLCAQARE AIHQGYTILI LSDRGFSPDR VPVSSLLAVG AVHQHLVLNL DRTRIGLLVD 788
BdNADH-GOGAT2  SKVLDITYPK KYGKKGLEQT LDKVCAQARE AIQEGYKILI ISDRGFSPDH VAVSSLLAVG AVHQNLVSNH ERTHVGLLVE 771
Consensus      SKVLDITYPK KRGRKGLEET LDRICAEARE AIKEGYTILV LSDRGFSSDR VAVSSLLAVG AVHQHLVSNL ERTRIGLLVE
```

```
                                  820                                     840                                     860                                     880
SpNADH-GOGAT   SAEPREVHNF CTLVGYGADA ICPYLAIEAI WRLQIDGKIP PKASGELHSR DELVQRYFKA SNDGMMKVLA KMGISTLASY 873
AtNADH-GOGAT   SAEPREVHHF CTLVGFGADA ICPYLAVEAV YRLQVDGKIP PKSNGEFHSK EELVKKYYKA SNYGMMKVLA KMGISTLASY 870
NnNADH-GOGAT1  SAEPREVHHF CTLVGYGADA ICPYLAIETI WRLQIDGKIP PKASGEFHSK EELVKKYFKA SNYGMMKVLA KMGISTLASY 865
NnNADH-GOGAT2  SAEPREVHHF CTLVGFGADA ICPYLAIETI WRLQVDGKIP PKASGEFHSK EELVKKYFKA STYGMMKVLA KMGISTLASY 865
OsNADH-GOGAT1  SAEPREVHHF CTLVGFGADA VCPYLAIEAI WCLQNDGKIP PNGDGKPYSK EELVKKYFYA SNYGMMKVLA KMGISTLASY 851
SbNADH-GOGAT1  SAEPREVHHF CTLVGFGADA ICPYLAIEAI WCLQNDGKIP PNGDGQLYSK EELVRKYFYA SNYGMMKVLA KMGISTLASY 847
BdNADH-GOGAT1  SAEPREVHHF CTLVGFGADA ICPYLAIEAI WCLQTDGKIP PNGDGQPDSK EELVKKYFYA SIYGMMKVLA KMGISTLASY 847
OsNADH-GOGAT2  SAEPREVHHF STLIGFGADA ICPYLAIEAI WRLQIDGRIP P-NDGKPYTQ EQLIEKYFYA SNYGMMKVLA KMGISTLASY 857
SbNADH-GOGAT2  SAEPREVHHF CTLLGFGADA ICPYLAIEAI WRLQIDEKIP PKDDGQLYPK E-LIDKYFYA SNNGIMKVLA KMGISTLASY 867
BdNADH-GOGAT2  SAEPHEVHHF CTLIGYGADA ICPYLAIEVI CRLQIDGRIP CTDGEQPYTQ EQLAQKYFNA SNYGMMKVLA KMGISTLASY 851
Consensus      SAEPREVHHF CTLVGFGADA ICPYLAIEAI WRLQIDGKIP PKXDGXPYSK EELVKKYFYA SNYGMMKVLA KMGISTLASY
```

```
                                  900                                     920                                     940                                     960
SpNADH-GOGAT   KGAQIFEALG LSSEVIEKCF NGTPSRVEGA NFEILARDAL RLHESAFPSR ALPPGSAEAV ALPNPGDYHW RKGGEVHLND 953
AtNADH-GOGAT   KGAQIFEALG LSSEVIQKCF AGTPSRVEGA TFEMLARDGL QLHELAFPTR GYAPGSAEAS ALTNPGNYHW RKNGEIHLND 950
NnNADH-GOGAT1  KGAQIFEALG LSSEVIQKCF AGSPSRVEGA TFEMLARDAL RLHEMAFPTR ALPPGSAEAV ALPNPGDYHW RKGGEIHLND 945
NnNADH-GOGAT2  KGAQIFEALG LSSEVIQKCF KGTPSRVEGA TFEMLALDAL QLHEMAFPTR AMPPGSAEAV ALPNPGDYHW RKGGEIHLND 945
OsNADH-GOGAT1  KGAQIFEALG LSSEVIRKCF DGTPSRIEGA TFEMLARDAL RLHELAFPSR APPPGSADAK ALPNPGDYHW RKNGEVHLND 931
SbNADH-GOGAT1  KGAQIFEALG LSSEVIGKCF EGTPSRIEGA TFEMLARDAL RLHELAFPSR TPPAGSADAK ALPNPGDYHW RKNGEIHLND 927
BdNADH-GOGAT1  KGAQIFEALG LSSEVIHKCF EGTPSRIEGA TFEMLARDAL HLHELAFPSR LPPAGSADAK ALPNPGDYHW RKNGEIHLND 927
OsNADH-GOGAT2  KGAQIFEALG LASEVVSKCF EGTPSRVEGA TFEMLAQDAL RLHEIAFPSR TLPPGSADAN ALPNPGDYHW RKNGEVHLND 937
SbNADH-GOGAT2  KGAQIFEALG LASEVVSKCF EGTPSRVEGS TFEMLAQDAL HLLKLAFPSR TLPPGSAEAN SLPNPGDHHW RKNGELHLND 947
BdNADH-GOGAT2  KGAQIFEALG LASEVISKCF EGTPSKVEGA KFDMLANDAL RLHDLAFPSR SWPHGSAEAN ALPNPGNYHW RKNGEVHLND 931
Consensus      KGAQIFEALG LSSEVIXKCF EGTPSRVEGA TFEMLARDAL RLHELAFPSR ALPPGSAEAX ALPNPGDYHW RKNGEIHLND
```

```
                                  980                                    1,000                                   1,020                                   1,040
SpNADH-GOGAT   PLAIAKLQEA ARANSVAAYK EYSRRVQELN KECNLRGMLK FKDVPGKINL DEVEPASEIV KRFCTGAMSY GSISLEAHTT 1033
AtNADH-GOGAT   PLAIAKLQEA ARTNSVAAYK EYSKRINELN KQSNLRGLMK FKDADVKIPL DEVEPASEIV KRFCTGAMSY GSISLEAHTT 1030
NnNADH-GOGAT1  PLAMAKLQEA ARSNSVAAYR EYSKRIQELN KSCNLRGMLK FKEAKVKVPL DEVEPASEIV KRFCTGAMSY GSISLEAHTT 1025
NnNADH-GOGAT2  PLAIAKLQEA ARSNSVAAYR EYSKRIQELN KSCNLRGMLK FKEAEVKVPL DEVEPASEIV KRFCTGAMSY GSISLEAHTT 1025
OsNADH-GOGAT1  PLAMAKLQEA ARVNSRAAYK EYSRRIQELN KTCNLRGMLK FKDTADMISV DEVEPASEIV KRFVTGAMSY GSISLEAHTA 1011
SbNADH-GOGAT1  PLAMGKLQEA ARVNSRAAYK EYSKRIQELN KACNLRGMLK FKDISDKISL DEVEPASEIV KRFCTGAMSY GSISLEAHTA 1007
BdNADH-GOGAT1  PLAMAKLQEA AKVNSRAAYK EYSKRIQELN KACNLRGMLK FKDISNKISL DEVEPASEIV KRFCTGAMSY GSISLEAHTA 1007
OsNADH-GOGAT2  PFSIAKLQEA ARINSREAYK EYSRRIYELN KACTLRGMLK FREIPNQISL DEVEPAKEIV KRFCTGAMSY GSISLEAHTS 1017
SbNADH-GOGAT2  PFSIAKLQEA ARLDSREAYK EYSRHTEELS KSCTLRGMLK FREIPVRISL DEVEPASEIV KRFCTGAMSY GSISLEAHTT 1027
BdNADH-GOGAT2  PLSIAKLQEA ARINSKEAYK EYSRLIQDNN KACTLRGMLK FRETRDRISL DEVEPASEIM KRFCTGAMSY GSISLEAHTT 1011
Consensus      PLAIAKLQEA ARVNSRAAYK EYSXRIQELN KACNLRGMLK FKXIPVKISL DEVEPASEIV KRFCTGAMSY GSISLEAHTT
```

```
                                 1,060                                   1,080                                   1,100                                   1,120
SpNADH-GOGAT   LAIAMNKLGG KSNTGEGGEN PSRLVPLPDG SMNPKRSAIK QVASGRFGVT SYYLTNADEL QIKMAQGAKP GEGGELPGHK 1113
AtNADH-GOGAT   LAMAMNKLGG KSNTGEGGEL PSRMEPLADG SRNPKRSSIK QIASGRFGVS SYYLTNADEL QIKMAQGAKP GEGGELPGHK 1110
NnNADH-GOGAT1  LAIAMNTLGG KSNTGEGGEQ PSRMQPLPDG SMNPKRSAIK QVASGRFGVS SYYLTNADEL QIKMAQGAKP GEGGELPGHK 1105
NnNADH-GOGAT2  LAIAMNKLGG KSNTGEGGEQ PSRMQPLPDG SRNPKRSAIK QVASGRFGVS SYYLTNADEL QIKMAQGAKP GEGGELPGHK 1105
OsNADH-GOGAT1  LAMAMNKLGG KSNTGEGGEQ PSRMEPLANG SMNPKRSAIK QVASGRFGVS SYYLTNADEL QIKMAQGAKP GEGGELPGHK 1091
SbNADH-GOGAT1  LAIAMNKLGG KSNTGEGGEQ PSRMEPLPDG SMNPRRSAIK QVASGRFGVS SYYLTNADEL QIKMAQGAKP GEGGELPGHK 1087
BdNADH-GOGAT1  LAVAMNKLGG KSNTGEGGEQ PSRMEPLPDG SMNPKRSAIK QVASGRFGVS SYYLTNADEL QIKMAQGAKP GEGGELPGHK 1087
OsNADH-GOGAT2  LAEAMNTLGG KSNTGEGGEQ PCRMVPLPDG SKNPRISAIK QVASGRFGVS IYYLTNAVEV QIKMAQGAKP GEGGELPGHK 1097
SbNADH-GOGAT2  MARAQNIKRA KSNTGEGGEQ PSRMEPLPDG SMNPLSSAIK QVASGRFGVS IYYLTNAIEL QIKMAQGAKP GEGGELPGHK 1107
BdNADH-GOGAT2  LAEAMNILGG KSNTGEGGEL PSRMELLADG SMNPRISAIK QVASGRFGVS IYYLTNAIEI QIKMAQGAKP GEGGELPGHK 1091
Consensus      LAIAMNKLGG KSNTGEGGEQ PSRMEPLPDG SMNPKRSAIK QVASGRFGVS SYYLTNADEL QIKMAQGAKP GEGGELPGHK
```

```
                                                  1,140                                   1,160                                   1,180                                   1,200
SpNADH-GOGAT   VIGDIAVTRN STAGVGLISP PPHHDIYSIE DLAQLIHDLK NSNPGARISV KLVSEAGVGV IASGVVKGHA DHVLISGHDG 1193
AtNADH-GOGAT   VIGDIAITRN STAGVGLISP PPHHDIYSIE DLAQLIHDLK NANPGARISV KLVSEAGVGV IASGVVKGHA DHVLIAGHDG 1190
NnNADH-GOGAT1  VIGDIAVTRN STAGVGLISP PPHHDIYSIE DLAQLIHDLK NANPGARISV KLVSVAGVGV IASGVVKGHA EHVLISGHDG 1185
NnNADH-GOGAT2  VIGDIAVTRN STAGVGLISP PPHHDIYSIE DLAQLIHDLK NSNPGARISV KLVSEAGVGV IASGVVKGHA DHVLISGHDG 1185
OsNADH-GOGAT1  VIGDIAVTRH STAGVGLISP PPHHDIYSIE DLAQLIHDLK NSNPRARISV KLVSEAGVGV VASGVVKGHA DHVLISGHDG 1171
SbNADH-GOGAT1  VIGDIAVTRH STAGVGLISP PPHHDIYSIE DLAQLIHDLK NSNPGARISV KLVSEAGVGV VASGVVKGHA DHVLISGHDG 1167
BdNADH-GOGAT1  VIGDIAVTRH STAGVGLISP PPHHDIYSIE DLAQLIHDLK NSNPQARISV KLVSEAGVGV VASGVVKGHA DHVLISGHDG 1167
OsNADH-GOGAT2  VIGDIAVTRN STAGVGLISP PPHHDIYSIE DLAQLIHDLK NANPGARISV KLVSEAGVGI VASGVVKGHA DHVLISGHDG 1177
SbNADH-GOGAT2  VIGDIAVTRH STAGVGLISP PPHHDIYSIE DLAQLIYDLK SSNPGARISV KLVSEAGVGV VASGVVKGHA DHILISGHDG 1187
BdNADH-GOGAT2  VIGDIAVTRH STAGVGLISP PPHHDIYSIE DLSQLIHDLK NANPGARISV KLVSEAGVGV VASGVVKAHA DHVLISGHDG 1171
    Consensus  VIGDIAVTRX STAGVGLISP PPHHDIYSIE DLAQLIHDLK NSNPGARISV KLVSEAGVGV VASGVVKGHA DHVLISGHDG
                                                  1,220                                   1,240                                   1,260                                   1,280
SpNADH-GOGAT   GTGASRWTGI KNAGLPWELG LAETHQTLVA NDLRGRTVLQ TDGQLKTGKD VAIAALLGAE EFGFSTAPLI TLGCIMMRKC 1273
AtNADH-GOGAT   GTGASRWTGI KNAGLPWELG LAETHQTLVA NDLRGRTVLQ TDGQLKTGRD VAVAALLGAE EFGFSTAPLI TLGCIMMRKC 1270
NnNADH-GOGAT1  GTGASRWTGI KNAGLPWELG LAETHQTLVA NDLRGRTILQ TDGQLKTGRD VVIAALLGAE EFGFSTAPLI TLGCIMMRKC 1265
NnNADH-GOGAT2  GTGASRWTGI KNAGLPWELG LAETHQTLVA NDLRGRTTLQ TDGQLKTGRD VAIAALLGAE EFGFSTAPLI TMGCIMMRKC 1265
OsNADH-GOGAT1  GTGASRWTGI KNAGLPWELG LAETHQTLVA NGLRGRAILQ TDGQLKTGKD VAVACLLGAE EFGFSTAPLI TLGCIMMRKC 1251
SbNADH-GOGAT1  GTGASRWTGI KNAGLPWELG LAETHQTLVA NGLRGRAVLQ TDGQLKTGRD VAVACLLGAE EFGFSTAPLI TLGCIMMRKC 1247
BdNADH-GOGAT1  GTGASRWTGI KNAGLPWELG LAETHQTLVA NGLRGRAVLQ TDGQLKTGRD VAVACLLGAE EFGFSTAPLI TLGCIMMRKC 1247
OsNADH-GOGAT2  GTGASRWTGI KNAGLPWELG LAETHQTLVA NGLRGRAVLQ TDGQMKTGRD VAVACLLGAE EFGFSTAPLI TLGCIMMRKC 1257
SbNADH-GOGAT2  GTGASRWTGI KNAGLPWELG LAETHQTLVA NGLRARVVLQ TDGQLKIGRD VVIACLLGAE EFGFSTAPLI ALGCIMMRKC 1267
BdNADH-GOGAT2  GTGASRWTGI KNAGLPWELG LAETHQTLVA NGLRGRAVLQ TDGQLKTGRD VAIACLLGAE EFGFSTAPLI TLGCIMMRKC 1251
    Consensus  GTGASRWTGI KNAGLPWELG LAETHQTLVA NGLRGRAVLQ TDGQLKTGRD VAXACLLGAE EFGFSTAPLI TLGCIMMRKC
                                                  1,300                                   1,320                                   1,340                                   1,360
SpNADH-GOGAT   HKNTCPVGIA TQDPVLREKF AGEPEHVINF FFMLAEEVRE IMAQLGFRTL LEMVGRSDML EIDEQVVNSN EKLENIDLSL 1353
AtNADH-GOGAT   HKNTCPVGIA TQDPVLREKF AGEPEHVINF FFMLAEEVRE IMSGLGFRTV TEMIGRADML ELDREVVKNN DKLENIDLSL 1350
NnNADH-GOGAT1  HKNTCPVGIA TQDPVLREKF AGEPEHVINF FFMLAEEVRE IMSQLGFRTI NEMVGHSDML EVDKEVVSNN EKLENIDLSL 1345
NnNADH-GOGAT2  HKNTCPVGIA TQDPVLREKF AGEPEHVINF FFMLAEEVRE IMSNLGFRTV NEMVGRSDML EVDKEVIKNN GKLENIDLSL 1345
OsNADH-GOGAT1  HTNTCPVGIA TQDPVLREKF AGEPEHVINF FFMLAEELRE IMSQLGFRTI TEMVGRSDML EVDPEVVKSN EKLENIDLSL 1331
SbNADH-GOGAT1  HMNTCPVGIA TQDPVLREKF AGEPEHVINF FFMLAEELRE IMANLGFRTI TEMVGRSDML EVDPEVVKSN EKLENIDLSL 1327
BdNADH-GOGAT1  HTNTCPVGIA TQDPVLREKF AGEPEHVINF FFMLAEELRE IMAQLGLRSI NEMVGRSDML EVDPEVVKSN EKLENIDLSL 1327
OsNADH-GOGAT2  HTNTCPAGIA TQDPVLRAKF AGKPEHVINY FFMLAEEVRE IMAQLGFRTV NEMVGRSDML EIDPKVLEGN EKLENIDLSR 1337
SbNADH-GOGAT2  HTNTCPVGIA TQDPVLREKF SGKPEHLINF FFMLAEEVRE IMSQLGFRTI NEMVGRSDML EVDPDVLKGN EKLQNIDLSL 1347
BdNADH-GOGAT2  HTNTCPVGIA TQDPVLREKF SGKPEHVINY FFMVAEEVRE IMSRLGFRTV NEMVGQADML EVDPEVLKGN EKLENIDLSQ 1331
    Consensus  HTNTCPVGIA TQDPVLREKF AGEPEHVINF FFMLAEEVRE IMSQLGFRTI NEMVGRSDML EVDPEVVKSN EKLENIDLSL
                                                  1,380                                   1,400                                   1,420                                   1,440
SpNADH-GOGAT   LLKPAAEIRP EAAQYCIQKQ DHGLDMALDN KLIVLSKAAL EKGLPVYIDL PVQNVNRAVG TMLSHEVTKR YRMDGLPSDC 1433
AtNADH-GOGAT   LLRPAAEIRP GAAQYCVQKQ DHGLDMALDQ ELIALSKSAL EKSLPVYIET PICNVNRAVG TMLSHEVTKR YHLTGLPKDT 1430
NnNADH-GOGAT1  LLRPAADIRP EAAQYCIQKQ DHGLDMALDN KLIALSTPAL EKGLPVYIEV PIRNVNRAVG TMLSHEVTKR YHMAGLPADT 1425
NnNADH-GOGAT2  LLRPAADIRP EAAQYCIQKQ DHGLDMALDK KLISLTKSAL EKALPVYIEL PIRNVNRAVG TTLSHEVTKR YHIAGLPADT 1425
OsNADH-GOGAT1  ILKPAAEIRP GAAQYCVEKQ DHGLDMALDN KLIALSKAAL EKEVRVFIET PIQNTNRAVG TMLSHEVTKR YHMKGLPAGT 1411
SbNADH-GOGAT1  ILKPASEIRP GVAQYCVEKQ DHGLDMALDN KLIDLSRTAI EKQVRVFIET PIQNTNRAVG TMLSHEVTKR YHMSGLPAGT 1407
BdNADH-GOGAT1  ILKPAAEIRP GAAQYCVEKQ DHGLDMALDN KLIALSKAAL EKQVRVFIET PIKNTNRAVG TTLSHEVTKR YHMKGLDSGT 1407
OsNADH-GOGAT2  LLKPAAEISP GAVQYCVEKQ DHGLDMALDN KLIASSTAAL RKGVRVFIET PVRNINRAVG TMLSHEVTKR YHIHGLPSDT 1417
SbNADH-GOGAT2  ILKPAAEISP EAVQYCVEKQ DHGLDKALDN KLIASSRAAL EKRFRVFIET LIKNTDRAVG TMLSHEVTKL FRMPGLPPDT 1427
BdNADH-GOGAT2  ILKPASKISP GAAQYCVEKQ DHGLHRALDN KLIALSRVAL EKGSRVFIET SVRNTNRTVG AMLSHEVTKR YHIHGLPSDT 1411
    Consensus  XLKPAAEIRP GAAQYCVEKQ DHGLDMALDN KLIALSKAAL EKGXRVFIET PIRNTNRAVG TMLSHEVTKR YHMXGLPXDT
                                                  1,460                                   1,480                                   1,500                                   1,520
SpNADH-GOGAT   IHIKLSGSAG QSLGAFLCPG ITLELEGDSN DYVGKGLSGG KVIVYPPRKS NFDPKENIVI GNVALYGATK GEAYFNGMAA 1513
AtNADH-GOGAT   IHIKFTGSAG QSLGAFLCPG IMLELEGDSN DYVGKGLSGG KVVVYPPKGS SFDPKENIVI GNVALYGATS GEAYFNGMAA 1510
NnNADH-GOGAT1  IHIKLGGSAG QSLGAFLCPG ITLELEGDSN DYVGKGLSGG KIVVYPPRES QFDPKENIVI GNVALYGATS GEGYFNGMAA 1505
NnNADH-GOGAT2  IHIKLTGSAG QSFGAFLCPG IMLELEGDSN DYVGKGLSGG KIVVYPPRKS QFDPKENIVI GNVALYGATS GEAYFNGMAA 1505
OsNADH-GOGAT1  IHVKLTGSAG QSLGAFLCPG ITLELEGDSN DYVGKGLSGG KIVVYPPRDS TFIPEDNIVI GNVALYGATI GEAYFNGMAA 1491
SbNADH-GOGAT1  IHVKFTGSAG QSFGAFLCPG ITLELEGDSN DYVGKGLSGG KIVVYPPRNS SFVPEDNIVI GNVALYGATK GEAYFNGMAA 1487
BdNADH-GOGAT1  IHVKLTGSAG QSFGAFICPG ITLELEGDSN DYVGKGLSGG KIVVYPPRNS TFIPEDNIVI GNVALYGATK GEAYFNGMAA 1487
OsNADH-GOGAT2  IHIKLNGSAG QSFGAFLCPG ITLELEGDSN DYVGKGLSGG KIVVYPPRNS RFNPQDNIVI GNVALYGATK GEAYFNGMAA 1497
SbNADH-GOGAT2  IHVKLNGSAG QSFGAFLCPG VTLELEGDSN DYVGKGLSGG KIVVYPPRNS RFIPQDNIVI GNVALYGSTK GEAYFNGMAA 1507
BdNADH-GOGAT2  IHVKLNGSAG QSFGAFLCPG ITLKLEGDSN DYVGKGLSGG KIVVYPPRNS RFNPQDNIVI GNVALYGSTK GEAYFNGMAA 1491
    Consensus  IHXKLTGSAG QSFGAFLCPG ITLELEGDSN DYVGKGLSGG KIVVYPPRNS RFDPKDNIVI GNVALYGATK GEAYFNGMAA
                                                  1,540                                   1,560                                   1,580                                   1,600
SpNADH-GOGAT   ERFCVRNSGA KAVVEGVGDH GCEYMTGGLV VVLGKTGRNF AAGMSGGVAY VLDADSTFKT RCNLELVDLD KVEEEDDITT 1593
AtNADH-GOGAT   ERFSVRNSGA KAVVEGLGDH GCEYMTGGTV VVLGKTGRNF AAGMSGGIAY VLDVDGKFNT RCNLELVDLD KVEDEEDKMT 1590
NnNADH-GOGAT1  ERFCVRNSGA KAVVEGVGDH GCEYMTGGTV VVLGKTGRNF AAGMSGGIAY VLDVDGKFQS RCNLELVDLD KVEDEEDIMT 1585
NnNADH-GOGAT2  ERFCVRNSGA KAVVEGVGDH GCEYMTGGTV VVLGKTGRNF AAGMSGGIAY VLDVDEKFQS RCNLELVDLE KVEDEEDIMT 1585
OsNADH-GOGAT1  ERFCVRNSGA QAVVEGIGDH GCEYMTGGTV VILGKTGRNF AAGMSGGIAY VYDIDGKFSV RCNHELVDLY HVEEEEDITT 1571
SbNADH-GOGAT1  ERFCVRNSGA QAVVEGIGDH GCEYMTGGAV VILGKTGRNF AAGMSGGIAY VYDVDGKFSA RCNNELVDLY HVEEEDDIIT 1567
BdNADH-GOGAT1  ERFCVRNSGA ITVVEGIGDH GCEYMTGGTV VILGKTGRNF AAGMSGGIAY VYDVDGTFSA HCNHELVDLY HVEEEDDITT 1567
OsNADH-GOGAT2  ERFCVRNSGA QAVVEGIGDH GCEYMTGGTA VILGKTGRNF AAGMSGGIAY VYDVDGKFSS RCNYELVDLY AVVEEDDITT 1577
SbNADH-GOGAT2  ERFCVRNSGA QAVVEGIGDH GCEYMTGGTV VILGKTGRNF AAGMSGGIAY VYDVDGMFST RCNHELVDLY NVDEEDDITT 1587
BdNADH-GOGAT2  ERFCVRNSGA EAVVEGIGDH GCEYMTGGIV VILGKTGRNF GAGMSGGIAY VYDVDGKFSS RCNHELVELY RVEEEEDIKT 1571
    Consensus  ERFCVRNSGA XAVVEGIGDH GCEYMTGGTV VILGKTGRNF AAGMSGGIAY VYDVDGKFSS RCNXELVDLY KVEEEXDITT
                                                  1,620                                   1,640                                   1,660                                   1,680
SpNADH-GOGAT   LRVMIQQHQR HTRSELAKEV LANFDELLPK FVKVFPRDYK RVLQNMKAEE AAKEAQ---- ---QREE-AE LMKKDAFQEL 1665
AtNADH-GOGAT   LKMMIQQHQR HTNSQLAQEV LADFENLLPK FIKVFPRDYK RVLSAMKHEE VSKQAIERAS EEADETEEKE LEEKDAFAEL 1670
NnNADH-GOGAT1  LRMMIQQHQR HTNSELAREV LANFENLLPK FIKVVPRDYK RVLANLRAEQ AAKDAKERAA KEAEEQEEAE LMEKDAFEEL 1665
NnNADH-GOGAT2  LRMMIQQHQR HTNSELAREV LADFDNLLPK FIKVYPRDYK RVLANMKAEQ AAK----KVV REAQEQEEAE LMKKDAFEEL 1661
OsNADH-GOGAT1  LKMMIEQHRL NTGSVVARDI LSNFDTLLPK FVKVFPRDYK RVLDNMKAEK AA-------A KLAKEP---- ---------- 1630
SbNADH-GOGAT1  LKMMIEQHRR NTESVLARDI LSDFDNLLPK FVKVYPRDYK RVLENMKAEK AV-------A KHAKEP---- ---------- 1626
BdNADH-GOGAT1  LKMMIEQHRL HTGSVLAKDI LSKFSSLLPK FVKVFPRDYK RVLEEMKAQK AA-------A SHAKEP---- ---------- 1626
OsNADH-GOGAT2  LRMMIQQHRL HTQSDLARDI LLNFDTLLPK FIKVYPRDYK RVLDKLKEEK A--------A KEAEQKAREV V-DKKPVEVI 1648
SbNADH-GOGAT2  LRVMIEQHRL NTESVLAKDI LSNFEDLLPK FVKVFPRDYK RVLDNMKAEK V--------A KEAEQKMRKK GWDKKAGEMI 1659
BdNADH-GOGAT2  LRMMIEQHRL NTESHLAKYI LSSFEDQLPK FVKVFPRDYK RVLDNLKVEK VAKA------ --AEEKTRKM LMDKKAGEET 1643
    Consensus  LRMMIXQHRX HTXSVLARDI LSNFDNLLPK FVKVFPRDYK RVLDNMKAEK AAK------A KEAEEXE-XE LMDKDAFEEL
```

```
                                                   1,700                         1,720                         1,740                         1,760
SpNADH-GOGAT   KKLASASINE KAN-GRIEEP QPLKRPTRVA NAVKHRGFVN YEREGVSYRD PNARIQDWKE -VSEESKPGP LLKTQSARCM 1743
AtNADH-GOGAT   KNMAAASSKE EMSGNGVAAE A---RPSKVD NAVKNGGFIA YEREGVKYRD PNVRLNDWNE -VMEESKPGP LLTTQSARCM 1746
NnNADH-GOGAT1  KKLAAASLND KAS-QKVEKA VQLKRPTKVD NAIKNGGFIA YERESISYRD PSNRISDWKE -VMEEPKPGP LLNTQSARCM 1743
NnNADH-GOGAT2  KKLALASSND RDKVNKVEQV VASKRPTKVD NAVKNGGFIA YERESISYRD PTVRVNDWEE -VMEESKLGP LLKTQSARCM 1740
OsNADH-GOGAT1  KISNGVSVTT K----KVQPE QSTNRPTRVS NAKKYRGFIS YERESISYRD PNERVKDWKE -VAIESVPGP LLNTQSARCM 1705
SbNADH-GOGAT1  KVANGISVTT K----KVQPD QSASRPTRVA NAKKYRGFIT YERESISYRD PKERVKDWKE -VAIESTPGP LLNTQSARCM 1701
BdNADH-GOGAT1  KVPNGVSVTT K----KIQTE QSTSRPTRVA NAKKYRGFIS YEREGISYRD PNERVKDWKE -VAIESVPGP LLNTQSARCM 1701
OsNADH-GOGAT2  QAPNGISVKT E----KVMNE EPSSRPSRVS NAVKYRGFIK YEREGTSYRD PNERVKDWNE -VAIELVPGP LLKTQSARCM 1723
SbNADH-GOGAT2  KAPNGISVIT K----KVQNK KSSSRPTQVF NAERPRGFVE YEQQGISYRD ENERVKDWGE -VTNELVPGP LLNTQSARCM 1734
BdNADH-GOGAT2  KASNGSSVVT K----QMNDR KPSGQPTQVS NAIKEQGFVI FGREEVSCRD PNERTKDWDE LVKNELVPGP LLRTQSARCM 1719
Consensus      KXXNGASVXT K----KVQXE QSSSRPTRVX NAVKYRGFIA YEREGISYRD PNERVKDWKE -VAXESVPGP LLNTQSARCM

                                                   1,780                         1,800                         1,820                         1,840
SpNADH-GOGAT   DCGTPFCHQD NSG--CPLGN KIPEFNELVH QERWREALDR LLETNNFPEF TGRVCPAPCE GSCVLGIIEN PVSIKSIECS 1821
AtNADH-GOGAT   DCGTPFCHQE NSG--CPLGN KIPEFNELVY QNRWQEALNR LLETNNFPEF TGRVCPAPCE GSCVLGIIEN PVSIKSIECA 1824
NnNADH-GOGAT1  DCGTPFCHQE NSG--CPLGN KIPEFNELVY QNRWREALDR LLETNNFPEF TGRVCPAPCE GSCVLGIIEN PVSIKSIECT 1821
NnNADH-GOGAT2  DCGTPFCHQE NSG--CPLGN KIPEFNELVY QNRWREALDR LLETNNFPEF TGRVCPAPCE GSCVLGIIEN PVSIKSIECA 1818
OsNADH-GOGAT1  DCGTPFCHQE SSGAGCPLGN KIPEFNELVH QNRWREALDR LLETNNFPEF TGRVCPAPCE GSCVLGIIEN PVSIKSIECA 1785
SbNADH-GOGAT1  DCGTPFCHQE SSGAGCPLGN KIPEFNELVH QNRWREALDR LLETNNFPEF TGRVCPAPCE GSCVLGIIEN PVSIKSIECA 1781
BdNADH-GOGAT1  DCGTPFCHQE SSGAGCPLGN KIPEFNELVH QNRWREALDR LLETNNFPEF TGRVCPAPCE GSCVLGIIEN PVSIKSIECS 1781
OsNADH-GOGAT2  DCGTPFCHQE GSGAGCPLGN KIPEFNELVH QNRWHEALDR LLETNNFPEF TGRVCPAPCE GSCVLGIIDN PVSIKSIECA 1803
SbNADH-GOGAT2  SCSTPFCHQE NFGAGCPLGN KIPEFNELVY QNRWREALDR LLETNNFPEF TGRVCPAPCE GSCVLGIIEN PVSIKSIECA 1814
BdNADH-GOGAT2  GCGTPFCHQE SSGAGCPLGN KIPEFNELVH DNRWREALDR LLETNNFPEF TGRVCPAPCE GSCVLGIIEN PVSIKTIECA 1799
Consensus      DCGTPFCHQE NSGAGCPLGN KIPEFNELVH QNRWREALDR LLETNNFPEF TGRVCPAPCE GSCVLGIIEN PVSIKSIECA

                                                   1,860                         1,880                         1,900                         1,920
SpNADH-GOGAT   IIDKAFEEGW MVPRPPLK-- RTGKKIAIVG SGPAGLAAAD QLNKMGHSVT VFERADRIGG LMMYGVPNMK ADKVDIVQRR 1899
AtNADH-GOGAT   IIDKAFEEGW MVPRPPLK-- RTGKKVAIIG SGPAGLAAAD QLNKMGHLVT VYERSDRIGG LMMYGVPNMK TDKIDVVQRR 1902
NnNADH-GOGAT1  IIDKAFKEGW MVPRPPLR-- RTGKRVAIVG SGPAGLAAAD QLNKMGHLVT VLERADRIGG LMMYGVPNMK ADKVDVVQRR 1899
NnNADH-GOGAT2  IIDKAFNEGW MVPRPPPK-- RTGKRVAIVG SGPAGLAAAD QLNKMGHLVT VFERADRIGG LMMYGVPNMK ADKVDIVQRR 1896
OsNADH-GOGAT1  IIDKGFEEGW MVPRPPLQ-- RTGKKVAIIG SGPAGLAAAD QLNKMGHFVT VFERADRIGG LMMYGVPNMK TDKIEIVQRR 1863
SbNADH-GOGAT1  IIDKGFEEGW MVPRPPLQ-- RTGRKVAIVG SGPAGLAAAD QLNKMGHFVT VFERADRIGG LMMYGVPNMK TDKIGIVQRR 1859
BdNADH-GOGAT1  IIDKGFEEGW MVPRPPLQ-- RTGKKIAIVG SGPAGLAAAD QLNKMGHFVT VFERSDRIGG LMMYGVPNMK TDKIGVVQRR 1859
OsNADH-GOGAT2  IIDKGFEEGW MVPRPPLR-- RTGKRVAIVG SGPAGLAAAD QLNKMGHFVT VFERADRIGG LMMYGVPNMK ADKEGIVQRR 1881
SbNADH-GOGAT2  IIDKGFKEGW MVPRPPLQSQ RTGMTVAIVG SGPAGLAAAD QLNKMGHYVV VFERADRIGG LMMYGVPNMK ADKARIVKRR 1894
BdNADH-GOGAT2  IIDKGFEEGW MVPRPPVH-- RTGKEVAIVG SGPAGMAAAD QLNKMGHRVT VFERSDRIGG LMMYGVPNMK ADKTRIVQRR 1877
Consensus      IIDKGFEEGW MVPRPPLQ-- RTGKKVAIVG SGPAGLAAAD QLNKMGHFVT VFERADRIGG LMMYGVPNMK ADKIDIVQRR

                                                   1,940                         1,960                         1,980                         2,000
SpNADH-GOGAT   VNLMAEEGVN FVVNAHIGVD PSYSLDILRS ENDAIILACG ATKPRDLPVP GREFSGVHFA MEFLHANTKS LLDSNLDDGK 1979
AtNADH-GOGAT   VDLMTKEGIN FVVNANIGKD PSYSLDGLKE ENDAIVLAVG STKPRDLPVP GRDLSGVHFA MEFLHANTKS LLDSNHEDGN 1982
NnNADH-GOGAT1  VNLMAEEGVN FVVNANVGTD PLYSIDRLRA EHDAIVLALG ATKPRDLPVP GRELKGVHFA MDFLHANTKS LLDSNLQDGN 1979
NnNADH-GOGAT2  VNLMAEEGVN FVVNANVGTD PLYSIDRLRA ENDAIVLALG ATKPRDLPVP GRELKGVHFA MEFLHANTKS LLDSNLQDGN 1976
OsNADH-GOGAT1  VNLMAEEGIT FVVNANVGSD PLYSIERLRS ENDAVILACG ATKPRDLGIP GRELSGVHFA MEFLHANTKS LLDSNLEDGR 1943
SbNADH-GOGAT1  VNLMAEEGVT FVVNASVGSD PLYSIERLRS ENDAVILACG ATKPRDLTIP GRELSGVHFA MEFLHANTKS LLDSNLEDGK 1939
BdNADH-GOGAT1  VNLMAEEGIT FVVNAHVGSD PLYSIERLRS ENNAVILACG ATKPRDLSIP GRELSGVHFA MEFLHANTKS LLDSNLEDGR 1939
OsNADH-GOGAT2  VELMAKEGVQ FIVNAHVGSD PLYSVEKLRS ENDAIILACG ATKPKDLPIP GRELAGIHFA MEFLHANTKS LLDSNLEDGN 1961
SbNADH-GOGAT2  VDLMDKEGVK FIVNAHVGTD PRYSIERLRS ENDAVILACG ATRPRDLPIP GRELSDIHFA MDFLHSNTKS LLDTNLEDGN 1974
BdNADH-GOGAT2  VDLMTEEGIT FVVNAHVGRD PSYSIDQLRS KNNAVILACG ATKPRDLPIP GRQLSGIHVA MEFLHANTKS LLDSKLEDGN 1957
Consensus      VNLMAEEGVX FVVNAHVGSD PLYSIXRLRS ENDAXILACG ATKPRDLPIP GRELSGVHFA MEFLHANTKS LLDSNLEDGN

                                                   2,020                         2,040                         2,060                         2,080
SpNADH-GOGAT   YISAKGKKVV VIGGGDTGTD CIGTSIRHGC TSVVNLELLP EPPAKRAPGN PWPQWPRIFR VDYGHQEAAT KFGKDPRSYE 2059
AtNADH-GOGAT   YISAKGKKVV VIGGGDTGTD CIGTSIRHGC TNIVNLELLP QPPSTRAPGN PWPQWPRVFR IDYGHQEATT KFGKDPRTYE 2062
NnNADH-GOGAT1  IISAKGKKVV VIGGGDTGTD CIGTSIRHGC TNIINLELLP QPPQTRAPGN PWPQWPRIFR VDYGHQEAAT KFGKDPRSYE 2059
NnNADH-GOGAT2  YISAKGKKVV VIGGGDTGTD CIGTSIRHGC TSIVNLELLP QPPKTRAPGN PWPQWPRIFR VDYGHQEAAT KFGKDPRSYE 2056
OsNADH-GOGAT1  YISAKGKKVV VIGGGDTGTD CIGTSIRHGC TSIVNLELLT KPPSKRAADN PWPQWPRIFR VDYGHQEASS KFGNDPRTYE 2023
SbNADH-GOGAT1  YISARGKKVV VIGGGDTGTD CIGTSIRHGC TNLVNLELLS KPPSNRAADN PWPQWPRIFR VDYGHQEAAT KFGKDPRTYE 2019
BdNADH-GOGAT1  YISAQGKKVV VIGGGDTGTD CIGTSIRHGC SSIVNLELLT KPPSKRAADN PWPQWPRVFR VDYGHQEASN KFGKDPRTYE 2019
OsNADH-GOGAT2  YISAQGRKVV VIGGGDTGTD CIGTSIRHGC TNLVNLELLP EPPRKRAPDN PWPQWPRIFR VDYGHQEATS KFGKDPRSYK 2041
SbNADH-GOGAT2  YISARGKKVV VIGGGDTGTD CIGTAIRHGC SNLVNLELLP EPPRERAPDN PWPQWPRIFR RDYGHQEAAS KFGKDPRTYQ 2054
BdNADH-GOGAT2  YISAQGKKVV VIGGGDTGTD CIGTSIRHGC SKLVNLELLP EPPTKRAPDN NWPKFPRIFR LDYGHQEAVS KFGKDPRTYQ 2037
Consensus      YISAKGKKVV VIGGGDTGTD CIGTSIRHGC TNIVNLELLP EPPSKRAPDN PWPQWPRIFR VDYGHQEAAT KFGKDPRTYE

                                                   2,100                         2,120                         2,140                         2,160
SpNADH-GOGAT   VLTKRFIGDD NGVVKGLEVV RVRWAKDASG RFQFQEIAGS EEIIEADLVL LAMGFLGPEQ KIAEELGVER DNRSNFKADF 2139
AtNADH-GOGAT   VLTKRFIGDD NGNVKGLELV RVSWEKDETG RFQFKEIEGS EEIIEADLVF LAMGFLGPEP TLAEKLGLEC DNRSNFKAEY 2142
NnNADH-GOGAT1  VLTKRFLGDE NGAVKGLEVV RVRWEKDESG RFQFKEIEGS EEIIEADLVL LAMGFLGPES TIADRLGLEK DNRSNLKADY 2139
NnNADH-GOGAT2  VLTKRFVGDE NGTVKGLEVV RVQWEKDANG KFQFKEIEGS EEMIEADLVF LAMGFLGPES TIADRLGLER DNRSNLKAEY 2136
OsNADH-GOGAT1  VLTKRFIGDE NGNVKALEVV RVKWEK-VDG RFQFKEIEGS NETIEADLVL LAMGFLGPEA TIAEKLGLEK DNRSNFKAQF 2102
SbNADH-GOGAT1  VLTKRFIGDE NGKVKALEVV RVKWEK-VDG RFQLKEIEGS EEIIEADLVL LAMGFLGPEA TIAEKLGLEK DNRSNFKAQF 2098
BdNADH-GOGAT1  VLTKRFIGDE NGKLKALEVV RVKWEK-VDG RFQFKEIEGS QEIIEADLVL LAMGFLGPEA TIPEKLGLEK DNRSTFKAQF 2098
OsNADH-GOGAT2  VLTKRFIGDE NGNVKALEVI RVEWGK-VNG RFQFKEVEGS EEIIEADLVL LAMGFLGPEA TVANKLGLEQ DMRSNFKAQF 2120
SbNADH-GOGAT2  ILTKRFIGDE NGKVKALEVV RVEWSK-VDG RFQFKEIEGS QEIIEADLVL LAMGFLGPEA AIAKKLGLEQ DNRSNFKAQF 2133
BdNADH-GOGAT2  VMAKRFIGDE DGKVKAVEVV HVEWEK-VDG RLQFKEVEGS EETIEADLVL LAMGFLGPEV TLADKLGLEQ DERSNFKAQF 2116
Consensus      VLTKRFIGDE NGKVKALEVV RVXWEK-VDG RFQFKEIEGS EEIIEADLVL LAMGFLGPEA TIAEKLGLEK DNRSNFKAQF

                                                   2,180                         2,200                         2,220
SpNADH-GOGAT   GRFSTNVEGI FAAGDCRRGQ SLVVWAIAEG RQAAAQVDRY LKDDGSFGGD DMASKISCHG DLEDLVKNPG SKRRVAA- 2216
AtNADH-GOGAT   GRFSTTVEGV FAAGDCRRGQ SLVVWAISEG RQAADQVDKF LTKTDDD--- ---EDAKLQQ DLNQMKHNTI TN------ 2208
NnNADH-GOGAT1  GKFSTNVEGV FAAGDCRRGQ SLVVWAISEG RQAAAQVDTY LMR-DSDPGT -SDSQ----- ---DVQKQQD SSRLTLMT 2207
NnNADH-GOGAT2  GKFSTNMEGV FATGDCRRGQ SLVVWAISEG RQAASQVDKY LTR-EGKHST TSGSQC---- --DSAKQQQD KNQFTART 2207
OsNADH-GOGAT1  GNFATSVDGI FAAGDCRRGQ SLVVWAITEG RQAAAAVDKY LSR-NEQ--- DAAEDITPSG --AGFVQPVA A------- 2167
SbNADH-GOGAT1  GDFATSVDGV FAAGDCRRGQ SLVVWAITEG RQAAAAVDKY LSR-YDQ--- NAAGDITPSG --AGLVQPVA A------- 2163
BdNADH-GOGAT1  GHFATSVDGV FAAGDCRRGQ SLVVWAITEG REAAAAVDKY LSRDEQ---- NSTQGITPSG --AGLVQPIT A------- 2163
OsNADH-GOGAT2  GNFATNVEGV FAAGDCRRGQ SLVVWAITEG RQAAAAVDNY LSK-DDEGET NGTEDIAVSS --EGLVQPVV A------- 2188
SbNADH-GOGAT2  ENFATNVEGV FAAGDCRRGQ SLVVWAIAEG REAAAAVDKY LSR-E---KT NVDENVVAPG PCECLVQPVA A------- 2200
BdNADH-GOGAT2  GNFATNVEGV FAAGDCRRGQ SLVVWAITEG REAAAAVDKY LS---DETKT NDADDIAASS --IGLLQAVA AWRHKQN- 2188
Consensus      GNFATNVEGV FAAGDCRRGQ SLVVWAITEG RQAAAAVDKY LSR-XDX--T NSAEDITPSG --XGLVQPVA A-------
```

B

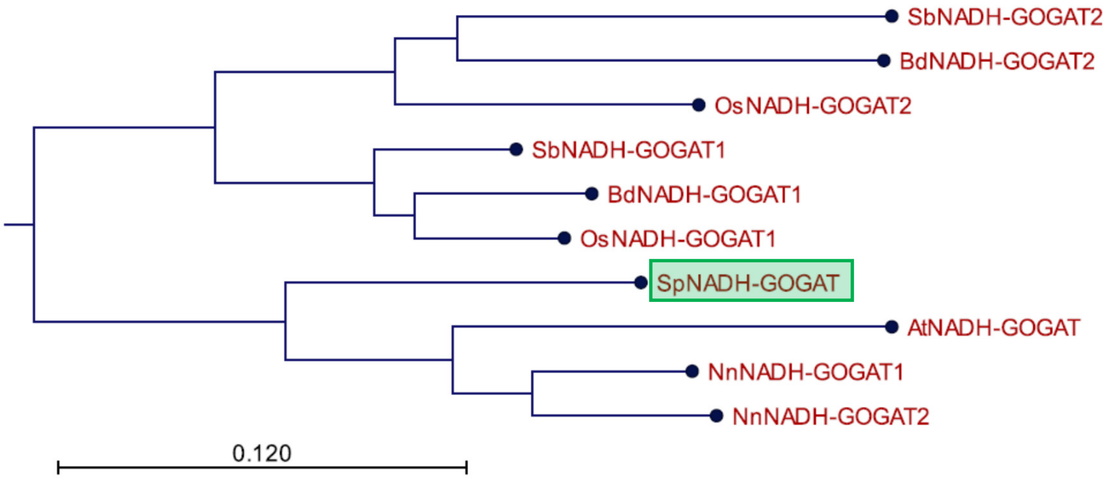

Figure S13. Sequence alignment NADH-GOGAT protein from *S.polyrhiza* with other representative species **(A)** and the resulted phylogenetic tree **(B)**. AtNADH-GOGAT - *A. thaliana,* NP_200158.2, BdNADH-GOGAT1 - *B. distachyon,* XP_003566997.1, BdNADH-GOGAT2 - *B. distachyon,* XP_024315185.1, NnNADH-GOGAT1 - *N. nucifera,* XP_010261570.1, NnNADH-GOGAT - *N. nucifera,* XP_010266511.1, OsNADH-GOGAT1 - *O. sativa,* XP_015649242.1, OsNADH-GOGAT2 - *O. sativa,* XP_015640407.1. SbNADH-GOGAT1 - *S. bicolor,* XP_002458326.1, SbNADH-GOGAT2 - *S. bicolor,* XP_021302649.1, SpNADH-GOGAT - *S. polyrhiza* (sequence translated from. Acc. OL421563)

SpNR

TATGCCGCGCCGTTTTTCATGCAGGAATGTATATACTTCAACGCATAAGTCCTAGTCAACTCTTGTTCCAGATTAACAAAAGTTT
TGTCCACTTATGGCCCCATAATTCCAGGAATAATATGTATGGATGATGCGCTTGCTTTCGTGGGGAGAAATGGTCTTCGGCATCC
TCTTGAA**GATTC**CCAGCGTTGTCCCTGGAGATA*TTCTCTCTCTCTCTCTCTCTCTCTCTCTCTCTCTCTCTCTCTCTCTCTCTCT*
*CTCTTTCTCTCTCTATCTCTCTCTCTATCTCTCTCT*AGCTCGGCAGGAGCCAAACTAGGACCCGACGCTGGAAACCGCGGCGCGA
GATAGAGCTGGGGAGCCAGGCCGGCGGGCGCGCCAAAGGGCGGGGACGAGCCTCAGACGACCGACTCTTGGCCCTCCGTCGATCC
ATAGAGCCGCCCTCCAACAACGCTCCCCTCCTGGTACGAGACTACCCCTGCAGAAGCTGTGAGCCCCGCTCGCCGCTTCCCCACA
GGAAGCTCCCGTCGGCGCGCCCCTTGGCCTAACGGGGGGCGGGCCGCGACGGCCGCCCAGGCCAAGCGGTGCCCCGCCGCATGAC
CGGTAAACCCAAGGCCGCCGCCGTCGAACGCCGCGGTGGGTGGGGGGAAGGGTCAAATGGGGCAGCGGGAATTTCCGTCATTCGT
CGTCATCCCCAGCCCACCCCTGCAACTTGAGAGGGGGGCGGCGGCGGGGTGGGGTGACCCC**TCGCCTTT**CCCTACCGCT**GGGAGC**
TGTTCGTGTTGGAAATCCGAGGGAACAGGACGTGCCGGCGGGCCCGCCGGCGATGCCCGGCACAGGCCGTGAGAGGAACACGTCG
TCCCCGGCTAGTAGTTTATGGCCGAGGTTCGTCTCCTCTCACCCGGCCTTCCTTCCACCTGGTTTATAAATCTGCAGC**TATCCTT**
TACACCTCACCGACG**AAGACTCG**CCCCCTCTGTTCATCCATCTGTCGGGAGGTGTGTCTGCTTTA**atg**

SpNiR

CAGTTTTAAGCTCTATTATGTTCAAGCCCCTATGAACCCTAGAATTAATGATCAGCCCCTTGCCTTTTCACTTTTTTAGATAGGC
ATCATAACCATGACTTCATTCCTCATTCGAATTGACAAGAATATTATTTTTAACATTATATTTACTTTTGGATAGTGGTGCTGAT
TTCCCTTGAGAAACAATACCAT*AGATAGAGAGAGA*CACAAAAATCTTGATTTAGCCATGATTAAAAAATTTTATTGTTCAGGGAG
AGTAATGGAGGAAAACAGACAGACAAATTCCAAATTTGGCATGTTTTGCAAGGACTTCCAGCAACAGGTGAAAAGGTGAGGGACT
CTTCTCTAAATCTAGTAGTTTACGAGTACCCCGATTTCTTTTTATAGTATAATAATACGATCTCCTTGAAAATAGAACCCCTTTG
ACTGGTCGTCCACGTGACGAGCTCTTGAGCTGCCGTGGAGCCAGCGGAGCGTTGACCAGCAAGAAGAATGTTCGCACAGGAAACA
TTTCGACGTGCAGCTGAAACTAATGGCGAGGATTTTTCTAACATTGTATTCGCACACAGTTTTTCTGTGGCGCCGATCGTCTTAT
CAAGAAACGAAAGCGGTGCCCGGGAGGCCTCGGTCCGACGGGCTGGCCCTGCTGTGCGGCTCACCGCCTGCGGTACGCGCCCCCC
CTCACGGCCGAGGGAGCCGCTGACCTGTGGCCCCACCCCACCATCCCCCCCCTCGCTGCCGCCGTCTTCTTCCGTCCCTA**CGACC**
CTTCTCTCGTCC**GTGAGTCC**CGCGTC**TGTCCTTT**AGAGAGAGCG**AAGAGCCC**AAGGCCAGATGCCCTCTCGTGCGACCGACTCTC
CGCCGCCGCTCGCTTGGGGGGCAACTCCGTCTCCCTCGTCG**TCACCTCT**CGAGGTGTT**AAGAACCCTGCGGCCTT**CCCACTGCC
CCGA**TGGAGTC**GCCACCGCCGCAGAGCAGCCCGCTGCCACCATCACCCGCCGACCTCGTACCGCC**atg**

SpGS1;1

ATTTAAATTATTTTATAATGTATACATTATTTATGGGCTGAAAAATATAGAGTTATTGCAGAAATTCAGACCCT
GGAAATTCTCTAAATTAACTGAAAATATAGGAAAAAATGTCAATAATTCTTGCCAGATAGATCTCCGGCCGCAA
GTATTATTGCCAACTTTTTTTCACATCAGTCCTCCAGGAATTCCATCCAGGCGGAAATTTCCCGAAATTATTTA
TTTCAATAGGCGGCGAAGAGAATAAGGGATATATTTTTATATCCTGTACTTTTAAAGTTTTTTAGTCCAAAGAA
AGCATTCTGGGGGGGCATATTACAATAATGTGGACGAATGACAGCTCTC**GATTC**GTCGTCAGATCGCATCCGAT
GACGACGTTTGTTCCCCAATCGGAATATGCCACGTCATCAAGCGCGCTCTAGATACCGCCGG**GAATC**TGTTCCC
ACTGGCAGAGTATCGTGAGATGGCCTAACTAAGTGGGGCCCACATGCAACACCAAATTACCTTAAAGCCCTCAC
CGGCAAGTCTAATCGCAGTCGACCCCCGTTGACCGGGCCCCACCCGAAAACTTTTTCCCCTTCATTAATACATC
TAATACTAAGTTAATTAATTGGAAACATAGTACTAACGTATTCGTATTTTACATATATTTAAAATTAATTAAAT
TTAAAAGTCAGCAGACTTGGAAGTCAAGCGTCTATAAGGGCAATATAGGCATTTCACCATACCACAACCTCCTC
CGAAAGGGACGC**GAATC**ACCGTAACAAATCGGTGACGTTACCGGCCATCCCGGAGTTCCGCGCTCCGCTAATAG
AAATTAATTTATTAAACTCTCTCCGTG**ACGGCTAT**TAAGATAGCA**GCGAGCCG**CTCCCCTGCGCCCAATCGCGT
CATCGCCTGGTGCATCTCCCCTGCTCTCGGATCTCGCCTCCTCTCTCCCCCTCCCCCTCCCTGGGGTTCTTCCC
GCT**GAATC**TTCCTTCCCCTCTCTCCCGTCTCTTCCGCC**atg**

SpGS1;2

CATCATATCGCCATAATTTTCCTTATAATAATCCCGCATTATTGTTTTTTAATGAGGGCCGTCGATGTGCCACG
CCAGGCCAAGTGGGCTAACAAAATAAGCCCGCGAAATTATAACAGCGA**GATTC**TTTTCCGCGGTTCC**GATTC**GA
TCAGGCGCGGAGGAAGGTAGCGGCCATTTATTGGCGACTGGGGGCTTCGGAAATCTGAA**GATTC**CGGGTGGCGT
TGACCAGCATGCTCTTGTAATAGCGCTTATCCATTAATGGGAAATAAATTATCCGACCGCCGGGATATTTTCGG
CCCAGGTGGCGGGCTTCGGTTATTACGACCCCTCCCCAGGTGAGTTACTCAGTGTGGTT**GAATC**TAGACACAGA
AAATCTAGATACGGGGGTGGGGCCCGGAGGCCACGTGGGGGCAGCTGGGTGGCGGGGAAGAGTGGACTCTCGGC

GGAGCTCGAACTTGATTATTCTGATTAAATGAGCGGATCTAGACCCGCAACCC**TCTGCCAG**TACGCAGCGG**AAG**
AGCCCGCCACATGCACTCGTGAGAGGAGAGGAATTTTGACCGGCATTGATGCATGCGTCACTTGTCACACTGAC
CCTCGAATGTAGTAAAGTTTTTGATCCGCGAGGGCAGTAGCGCCATTTAAGA**GAATC**GTCTCTCTCTCTAGTTG
CGGGAAAATCGGTCAGCGGGGGAGCAAGTGGGGCCTACAGCTCCTCCGTGGCGGCGCAAAGTTACCGTACCAAC
CAGTTTTCGGCGCCGATGTCAGGCTTCTGACTTTTGGGTGCGTC**CTCCCTCTT**CCTGTCGCAGC**AGGAGGAA**GG
GAGGGGAGGGTGGGGGCGGGCCACTGGAGATTTCTTCCTTGTTTTTTAAGGCTT**GAATC**CGTCTATATAAAGCG
CCCCCGCCGCTTCCCCACCAATCCATTCACCGCAGCGCTCTCCCTGCCCGGCGGATTGATCGCAGTTCCCTCTC
CCGCCCCGCCTCCTGCGTCTCCAGCTTCGGCCGCTGCC**atg**

SpGS1;3

CCAACCCTGGGTTGACCAGCGTCTTTCTAATGAGATCCTCCATTTTAGTAAGGCGTAAACTTGCATTATTTCAAACAAAGAGGGG
CTCAGTACATCCACGGTCGAGAACAGGGCAGTGGGCGGCGGCGGAGCGTTGGTAGATTTCTTCACTGTGCGGCGGCGGCGGTGGC
GTCGGCGACCCTACGAGGGCGGAGATGGGAATGGCTCAAACAGCGGCGGGCAGCAGTTCCAGAGGCCGCAGCAGCTGAAGAACCA
CAGGCTGCAGAGAATTCGAGGTCGAGGAGATGAGGGCAAAGAGAGAATTGACACTTGGAGGGAGGGGGAGTGAGAGACGAAGGGG
G**GATGCTTA**CCATGAAGCT**AAGAGCCC**GTCGGCTCCCGTGGCCTCTCAGCAGCGGCGGCGGCGGCAACGATGGCGGCGGCGGCAG
GGGCAGTGGGGGGGCAGCAGCAGGGGCGGCCTCTTGTGGCATGGGGGGGTCCATCGTTTCAGAGGAAGGAACAAGGGGGATGAAG
GAGAGATCTGTTCTTCTTCTAGGGTGGACTCTTCCTCCAGGGAGATCACCATCTGCCCTTCCAGCGCCGCCACCGAGCGGTCAAG
AGTTC*AGAGAGACGAGAGACGGAGAGAGAGAGAGAGAG*GTGGACTATTGGACCGAGCTGATATGGGTGGAGTCTACTTTGGTGTT
CAAGGCCAAATCCATATAACAGTGGGCCTGTCTCCAGTGGGTTTGGGCCTATCCCCCCCCCCCCCATTTGGCCGATTAAGCCCAA
TAAATATATATGCGATGGAAGAGGCCCATCTCTGTGATCATCATCACGTGAGGGCGTCGGCCCTCGAATGCCACCTATTTCGGCT
GCCACCTCCCACCGCCCAAGAGCGCGGTGTTAGGCCGCCACGTAGGAACTCCGAGCTCTTTTTCCGATGCTGATTTTGTCGTTTC
TGGTTGGTTACAGTCTCCTCCTCCCCAGGGGAGTAGCGCCTGCGTGCGAGTTCCCCCAGTCGGCC**atg**

SpGS2

AGATGAATAAAAATACCCTTTTTGTCAGAGCGGCAGCAGACGGATGTGTGTATGAGAGAGAGATGGCGCCCTTATCT**GAATC**CCT
TGGTCTTTGAGGGCTCCCAAGGGACCCGT**TTTCCTCC**ACTCAAAGCGG**AAGAACCC**TATCTGCTGGCGAAGAGCCGCCGAGGCGA
AGCTCGCCTCTCCCCTCACACAGCTTAATCCGCCGTGTCCTGGGCACGTGGACCATAAACAGCTGAGAACCTCTCTTTTCTAGAG
TATAAACGGTCCACCCG*CTCTCTCTCCCTCTCTCTCTCTCTCTC*GCTGTGTTTGCAAATCTTCTCTCCTCCTCCCCAACGACG
CCGCCGCCGCCGGAGGAGCTGGTGGGCGCCCGCATTACCTCCCTGCCTCGCCGTCGCCCGGCGTCTCTCGGTTAG*CTCTCCCTCC*
*CTCCCTCCCTCTCTCTCTCTCTCCCTATCTCTCT*ATCTCCTTCCCATGGGCTGCCCGAGGACTGTGGAATGACGACCTCATTGGG
GAGCAAGGCCGGCGTTGACCTTCGCCGCTTCGAAGGGTTCCTGTGCATTTCTCGTCATTCGATGTTTACATGTA**GATTC**ACGGGT
TTCGGTGGGGTGGGTCCTTCCGAAATCTGCATCAGACCGGCGCCTCATCGTTTCGGTTGGCTCGGGTTTTTTATTTACTCCGGGT
TGACCCTGCAAGATGCGGTTCTCGCCCTCGCTACGTGGCGTG**GATTC**ATTCCTGGCCCCCGGAGCGTTCATTCTTCAGAGGGGGG
ATCCCCTCCATTTTTGTCGTGCGGATCCTCTGTGTTCCCTTTCGAGTATCTCCCATGTCGATCCGAATGTCTCCGTTCGCCTTGC
CG**ATTGCGGTT**G**GATTC**TCC**GAGAGATT**GTTGC**TGCCTTTT**GTTTTTATGATCC**ACGAGGCG**AATTGCGAATTCTGTTTCCATCT
TCGGATCTTGATCAAACTAGTTGCAGAGCGGAGGAGAAGAGAGGGGACAGAAGGCGGAAGGAAAG**atg**

SpFd-GOGAT

GTTCTTTTTCCGAGGTAAGTCCAGTTATTTCAAATATTTTTATTTTTATCTTTCCATATTCTCCAATGGGGGAGTACATCTAGGA
TCATTTCGAAGAGAGAGAGAGCGCCGAAAAAGCCAAGAAGCGAGAGGCAGG*GAGAGGAGAGAGCGGAGAGGGACAGAGAGACAGA*
GAGGGAGGGGAGAGAAGAGAGAGAAAGGTGAGTGAGAGGAGGAGAGAGAGAGGGGGGGATAGAGAAAGAATGAGAGGAGAGAGAA
GAGAGAGAGAGGGTGGGATAGAGAAAGAGAGACTGAGAGGAGCGAGAAGAGAGAGAGAGGGTGGGATAGAGAAAGAGAGACTGAG
AGGAGCGAGAAGAGAGAGAGAGAGAGAGAGAGAGAGAGTGAGAGGAGAGAGAAGAGAGATGAAGAGAGGGGGAGAGAGAGAGAGAGA
*GAGAGAGAGAGAGAGAG*GGTCCGAGGATAGTGGTTGGCAGGGTGCGGTAGAGGACAAGGCAGCGGCTCCCACCAGTGCCTGTGTC
CTCCTCCCGGAGCTCTACTCCATTCCCCTCTCCGCACGACGACGACGACTACGAGCGCTGGAGGCGAGTGGCTCACATCTCTCCC
CTCCCCGCTCTCTCATTCTCGCTCTAACC**TCTCTCTAT**C*CTCTCTCTC*****TGCAGC*****CT**CTCTCTCTCTCTCTCTCTCT*ATCTGTTGG
CCAATCCTCCCGGCTTGGCAGCGCGGGCGATCAGGATTATCCTGCTCTT**TGGCGTTT**CCTCTCCAC**GCGAGCCT**CTCCATTTTCG
GATTTATATATTTGCGCATCTTCAAACTTCCTGCATATTTTATTGGCGTCGCGTATAAATTTTCGCTTCCCTGGTTTTGTTTTCC
TGCTCTGCCGACCAGAAAAAGTCTCCTCCTCTCCCACCTCGTCTCCTCCTCCTCCTCTTCCACTTCGGTTCCCTGGGGGATCCTG
CTCTGCCGGAGCGGTGGT**GAATC**AAGCGCGGCAGAGAGGAACGGGAGGGAGGGGAGGAAGGTGGG**atg**

SpNADF-GOGAT

TTTATAAAAAAATGGAGAATTTCTCCTCCGCATCGCCCCAAGTATCTTTTAAAAAATAGAAGCGTCACGAACCATCCGTCGCCAC
GCGTGAAAAGAATAATAATTATTCAATGATCATCCGACAACTGTTAAATAATATATTCCCCATGCAAGAATATTAAATAATGCCG
TCTTCTTCATGAGGCATCAAACTACGGGAGCAATACTTTCC**GATTC**AAAGAAATATCCCGGGGCCTCCGCCTTCACCGTGTAAAT
TTCCTCGTCTGCTTGCCCCAGCGCGCCACGT*AGAGAGAGAGAGAGAGAGAGAGAGAGAGAGAGAGAGAGAGAGAGAGAGAGAGAG*
*A*GGGGGAGGGAGCGGGGTGCCCCCCGACTGCGTGGTCAACGCCCCGCCGATACCAGGGGATTAGGGGAAAAAGCTCGCGGGGCTT

CGGCAATTCTACTTAAAGAATGATAAGTGGGGGTGGGCCCCACGAGATGCGATCGTCGCGTACTGCGGCGGGAGAGTGACGTTGG
GGATTGGGGCTGGGTTGGGAAATATCCCAAGCATCACGCTCTCTCAGGTTTTCTTTTATTTTTTAATTTAGAAAATAGTTTTATT
AGTTACAGAGTGTTGCCTGTTCAACCGGTGCGAGGGGACCGGGGTTGGGAGAGTCTGGCTCTGAG**GAATC**CCGAAGCTCGTCTAT
AAGTAGATATCCGCGGCGGGCGGCGGAGGAAGGGGAGTCGTCGTTGGTCGAGGAGGAGGGGAGAAGGAAGGGCTGTGGCTGAGTT
CCTCGGAGTTTACCCCGAGGGTGGAAGCTTCTGCTGCCCGTTCC**TGTTTTTTT**TTTTTTTAATTTTT**GGGAGTT**CTTCTCGTCAT
CTCTGTTCGA**TCTTCTTTTT**CGCCGCCGGC**GGTGGTTC**CGA**TCCCGCTT**TTGCGGTC**GAGAACCT**TGGCGGCCTGTTGCCGGATC
TGTCGTTTCTGGAAAGCCGGCCGAACATACGCCTTCCAGTTTGAGAGGGGGTCTAGGGTAGGGGG**atg**

Figure S14. Nucleotide sequences representing 1kb promoter regions upstream of the starting ATG codon of six Nitrogen assimilation genes of *S. polyrhiza* with marked locations of NRE-like *cis*-elements, GAGA and TCTC stretches and TATA-boxes.

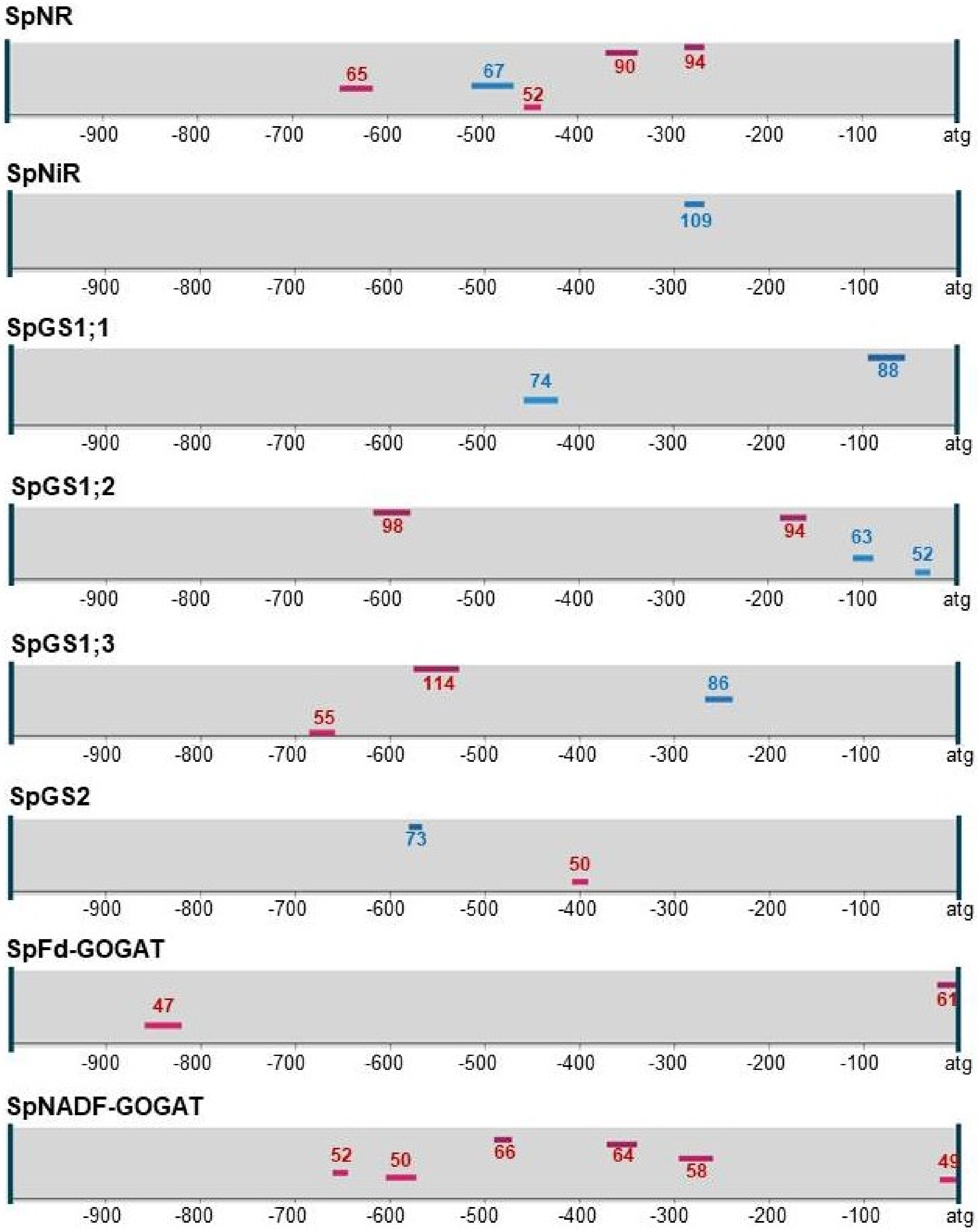

Figure S15. Patterns of G-quadruplex structures predicted for promoters of key *S. polyrhiza* genes involved in nitrogen assimilation, using the *pqsfinder* tool (https://pqsfinder.fi.muni.cz). Positions of G-quadruplex structures are indicated by horizontal lines (**red** for forward DNA strand, **blue** for reverse strand); Numbers next to bars indicate the relative strength of each G-quadruplex structure, and the negative (–) numbers indicate positions relative to the genes' translation start.
